# A novel lncRNA as a positive regulator of carotenoid biosynthesis in *Fusarium*

**DOI:** 10.1038/s41598-020-57529-2

**Published:** 2020-01-20

**Authors:** Obdulia Parra-Rivero, Javier Pardo-Medina, Gabriel Gutiérrez, M. Carmen Limón, Javier Avalos

**Affiliations:** Department of Genetics, Faculty of Biology, University of Seville, E-41012 Seville, Spain

**Keywords:** Fungal genetics, Long non-coding RNAs

## Abstract

The fungi *Fusarium oxysporum* and *Fusarium fujikuroi* produce carotenoids, lipophilic terpenoid pigments of biotechnological interest, with xanthophyll neurosporaxanthin as the main end product. Their carotenoid biosynthesis is activated by light and negatively regulated by the RING-finger protein CarS. Global transcriptomic analysis identified in both species a putative 1-kb lncRNA that we call *carP*, referred to as *Fo*-*carP* and *Ff*-*carP* in each species, upstream to the gene *carS* and transcribed from the same DNA strand. *Fo*-*carP* and *Ff*-*carP* are poorly transcribed, but their RNA levels increase in *carS* mutants. The deletion of *Fo*-*carP* or *Ff*-*carP* in the respective species results in albino phenotypes, with strong reductions in mRNA levels of structural genes for carotenoid biosynthesis and higher mRNA content of the *carS* gene, which could explain the low accumulation of carotenoids. Upon alignment, *Fo*-*carP* and *Ff*-*carP* show 75–80% identity, with short insertions or deletions resulting in a lack of coincident ORFs. Moreover, none of the ORFs found in their sequences have indications of possible coding functions. We conclude that *Fo-carP* and *Ff-carP* are regulatory lncRNAs necessary for the active expression of the carotenoid genes in *Fusarium* through an unknown molecular mechanism, probably related to the control of *carS* function or expression.

## Introduction

The genus *Fusarium* comprises a large group of phytopathogenic fungi widely distributed in nature. Some species are well-known research models for basic biological processes, such as *Fusarium oxysporum* in phytopathogenesis^1^ and *Fusarium fujikuroi* in secondary metabolism^2^. *F. oxysporum* comprises many species-specific plant pathogens, called *formae specialis*, and some of them have been widely used for the study of pathogenesis mechanisms and fungus-plant interactions^3,4^. *Fusarium fujikuroi* was first known for the production of gibberellins, growth-promoting plant hormones, and has later been investigated for the synthesis of other metabolites, such as bikaverin and fusarins^5,6^.

Some *Fusarium* species are also models for the investigation of carotenoid biosynthesis and its regulation^7^. Early studies in *Fusarium aquaeductuum* revealed the accumulation of xanthophyll neurosporaxanthin (NX), a carboxylic apocarotenoid previously discovered in *Neurospora crassa*. Subsequent studies in *Fusarium fujikuroi* led to the identification of all the genes of the NX pathway^7^, *carB*, *carRA*, *carT*, and *carD*, which respectively encode a desaturase, a bifunctional phytoene synthase/carotene cyclase, a torulene cleaving dioxygenase, and an aldehyde dehydrogenase (Supplementary Fig. S1). The *carB* and *carRA* genes are organized in a coregulated cluster with another dioxygenase gene, *carX*^8^. The CarX enzyme is capable of cleaving β-carotene, a side product of NX biosynthesis^9^, into retinal^10^. A fourth gene in the cluster is *carO*, which encodes a photoactive rhodopsin that uses retinal as chromophore^11^.

The biosynthesis of carotenoids in *Fusarium* is upregulated by light^12^ through transcriptional activation of the structural genes of the pathway, in which the White Collar photoreceptor WcoA plays a central role^13^. The transcriptional response is severely impaired in the absence of a functional WcoA protein, but there is an apparent accumulation of carotenoids in *wcoA* mutants under illumination^14^ that involves the participation of at least a second photoreceptor. This could be DASH cryptochrome CryD^13^, as indicated by the slower accumulation of carotenoids in Δ*cryD* mutants despite activation by WcoA^13^. CryD photoactivity has been demonstrated biochemically^15^, but only minor effects were found in the mRNA levels of *car* genes in Δ*cryD* mutants after illumination, suggesting its participation in a post-transcriptional regulatory mechanism. Another photoreceptor protein that participates in the regulation of carotenogenesis by light in *Fusarium* is VvdA, an orthologue of the Vivid protein of *N. crassa*, which supposedly counteracts WcoA activity^16^.

A class of mutants of *F. fujikuroi* exhibits deep orange pigmentation in the dark due to the synthesis of large amounts of NX and carotenoid precursors^17^. These mutants, also found in *F. oxysporum* and generically called *carS*, are affected in a single gene that encodes a protein of the RING-finger family^18,19^. The deep orange phenotype is due to the presence of large amounts of transcripts for the structural genes of the pathway, especially those of the *car* cluster, which indicates that CarS is a negative regulator of the expression of the *car* genes. This cluster is not the only regulatory target of CarS; the *carS* mutation produces noticeable changes in the mRNA levels of hundreds of genes, a notable proportion of them also regulated by light^20^. The mechanism of action of CarS remains unknown, but RING-finger domains belong to a family of E3-like ligase involved in the labelling of target proteins by ubiquitylation, a regulatory signal that frequently leads to their degradation. The alleged ortholog of CarS in *Mucor circinelloides*, CrgA, has been the subject of detailed investigation and its ubiquitylation activity has been demonstrated^21–23^. CarS and CrgA also contain a LON protease domain, whose function has not been determined.

Due to its regulatory effects on a large collection of genes, we assume that *carS* expression may be subject to complex regulation. The *carS* gene is preceded by a 4-kb upstream sequence without predicted ORFs in genome annotations. A former study of two *F. oxysporum* T-DNA insertion mutants, which exhibited deregulation of carotenoid biosynthesis, revealed sequence alterations in this 4-kb DNA segment upstream of the *carS* gene^18^, and a recent RNA-seq study suggested the occurrence of an unidentified transcript in this genomic region with features consistent with a long noncoding RNA (lncRNA). LncRNAs are polyadenilated RNAs of more than 200 bp in length, transcribed by RNA polymerase II but without protein coding functions. It has been discovered that an increasing number of lncRNAs are involved in important biological processes in higher eukaryotes, such as development or cancer. They modulate the expression of target genes through a variety of mechanisms^24–26^, associated with their sequences or their structure, involving transcriptional, post-transcriptional, and epigenetic levels. Thus, they can interact with transcription factors, chromatin remodelling factors, microRNAs, or mRNAs, and in some cases they can act as enhancer-like RNAs^27^. However, there is very little information on the functions of lncRNAs in lower eukaryotes. We have found that the transcript found in the genomic region upstream of *carS* plays a key positive role in the regulation of carotenoid biosynthesis in *F. oxysporum* and *F. fujikuroi*, probably through the control of the *carS* gene. This is the first functional study of a lncRNA in a species of *Fusarium*.

## Results

### Identification of a transcript upstream of the *carS* gene

**Figure 1:**
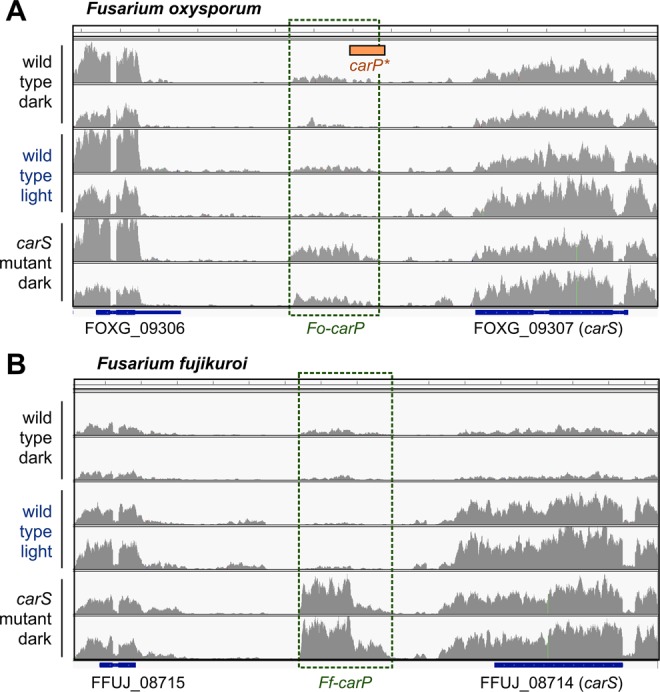
Effect of light (one-hour illumination) and *carS* mutation on transcript readings according to RNA-Seq data in the genomic region between FOXG_09306 and *carS* (FOXG_09307) genes in *F. oxysporum* (panel A) (*carS* mutant: SX1), and between Ffuj_08715 and *carS* (FFUJ_08714) genes in *F. fujikuroi* (panel B) (*carS* mutant: SG39). The location of a putative microRNA precursor sequence found in *F. oxysporum*, called carP*, is indicated with an orange box. In each condition, the profiles obtained with two independent biological samples are shown. The readings were represented with IGV program.

In a search for putative regulatory elements in the 4-kb upstream sequence of *carS* in *F. oxysporum*, a bioinformatic microRNA prediction tool identified a putative precursor microRNA sequence, which we will call here carP*. This alleged microRNA was not confirmed by a specific RNA-seq study for small RNAs (J. Pardo-Medina, unpublished), but a recent RNA-seq analysis on the effect of light and *carS* mutations in *F. fujikuroi* and *F. oxysporum*^20^ revealed a transcript in both species, which overlaps with the previous sequence of carP* in *F. oxysporum* (Fig. 1) and which we have called *carP*. The transcripts of *carP* have a length of 1195 bp in *F. oxysporum* and 1357 pb in *F. fujikuroi*, considering as initial and final nucleotides those present in at least 2 readings of the RNA-seq data in different strains and culture conditions (Supplementary Fig. S2A). To distinguish the versions of *carP* in both species, we call them *Fo*-*carP* and *Ff-carP*.

**Figure 2:**
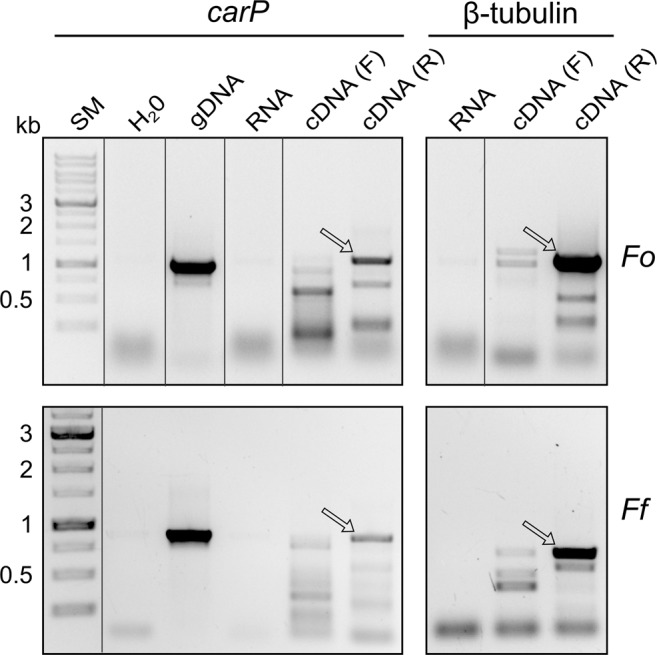
Determination of the orientation of the transcription of *carP* in *F. oxysporum* (*Fo*) and *F. fujikuroi* (*Ff*) through the amplification from specific single stranded DNA (ssDNA). SM: size markers. gDNA: amplification of the *carP* transcript using genomic DNA of the wild strain grown in the dark (positive control) as template; RNA: lack of amplification of the *carP* transcript using as template total RNA (negative PCR control and DNA contamination control in the ssDNA samples). cDNA (F): lack of amplification of *carP* from ssDNA obtained from a retrotranscription of total RNA with a mixture of forward *carP* primers. cDNA (R): amplification of *carP* from ssDNA obtained from a retrotranscription of total RNA with a mixture of reverse *carP* primers. Results are also shown for amplification of the β-tubulin gene as a control with a known transcriptional orientation. Lanes cropped from different positions of the original gels are separated by a black line. Full-length blots/gels are presented in Supplementary Fig. S13. The sizes of the expected PCR products are 1099 bp for *carP* and 1169 bp for β-tubulin gene in *F. oxysporum*, which correspond to 910 and 693 bp in *F. fujikuroi*. The primers used are described in Supplementary Table S5A. Genomic DNA and total RNA samples were obtained from wild strains grown for 3 days in darkness.

To determine the orientation of *carP* transcription, a PCR assay was carried out to identify strand-specific PCR products. For this purpose, we obtained cDNA samples using mixtures of primers for each DNA strand of the *carP* sequence and we used them as substrates for amplification with specific primers of *Fo*-*carP* and *Ff*-*carP*. As a control, the same protocol was used to determine the orientation of the β-tubulin gene. The results (Fig. 2) indicated that both genes encoding *carP* are transcribed from the same strand as the neighbouring *carS* gene.

### Sequence features of *carP*

**Figure 3:**
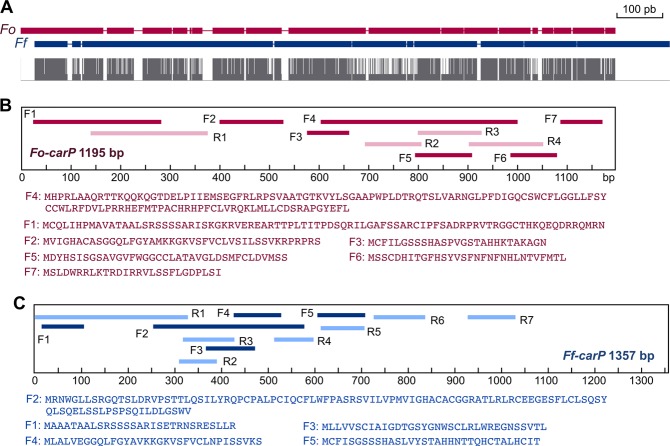
Sequence features of *Fo-carP* and *Ff-carP*. (**A**) Schematic representation of the clustal alignment between *Fo-carP* (red) and *Ff-carP* (blue). Gaps resulting from the alignment are indicated. The matching bases are shown below as long grey lines. (**B**,**C**) Open reading frames (ORFs) in *Fo-carP* (B, in red) and *Ff-carP* (C, in blue). The positions of each of the ORFs are represented in the boxed panels (F: forward, in dark color; R: reverse, in pale color). Residues corresponding to forward ORFs are shown below each boxed panel. The analysis of the ORFs was done through the ORFfinder tool.

A Clustal alignment between the *carP* sequences of *F. oxysporum* and *F. fujikuroi* revealed high conservation, with 929 matches along an overlapping stretch that covers 1165 bp in *Fo-carP* and 1238 bp in *Ff*-*carP* (Supplementary Fig. S2B), with a non-homogenous distribution of coincidences, and the occurrence of numerous gaps (Fig. 3A). To consider their possible coding functions, open reading frames (ORFs) in both chains with a threshold of 25 consecutive amino acids were determined. The results (Supplementary Fig. S3) showed 11 ORFs for the *Fo-carP* sequence, 7 of them in the forward direction (Fig. 3B), and 12 ORFs in *Ff-carP*, of which 5 in the forward direction (Fig. 3C). The longest ORFs encoded 131 residues in *Fo*-*carP* and 107 in *Ff*-*carP*, located in different regions of *carP*. ORF F4 of *Fo-carP* was previously annotated as hypothetical proteins FOTG_04033 in *F. oxysporum* f.sp. *vasinfectum*, and FOVG_10518 in *F. oxysporum* f.sp. *pisi*, but they were not annotated in other *Fusarium* genomes. Comparisons of the ORF sequences with those of the protein databases did not identify known conserved sequences or domains. E.g, the best match of the *Fo*-*carP* sequence gave an E value of 0.005. Most significantly, with the exception of some short segments (Supplementary Fig. S3), there are no coincident ORFs in the *carP* sequences between both species.

Taking into account the possible occurrence of intron sequences, we achieved BlastX ORF-independent searches with whole *carP* sequences against the NCBI nr database. Without considering the two annotated *carP* segments of *F. oxysporum*, the maximum E value was 0.3, found against a cyclase of *Streptomyces* sp. As alternative approaches, the coding capacity of *carP* was verified through other sequence characteristics. Analyses based on 3-base periodicity (Supplementary Fig. S4) or codon usage (Supplementary Fig. S5) did not provide evidence of coding functions. Other methods used to assess the protein-coding potential of *carP* were two versions of Coding Potential Calculator: CPC^28^ and CPC2^29^, based on intrinsic sequence features, and RNAcode, based on comparative sequence data^30^. None of these methods provided any support to the occurrence of putative coding sequences in *Fo-carP* or *Ff-carP* (Supplementary Tables S1 and S2).

**Figure 4:**
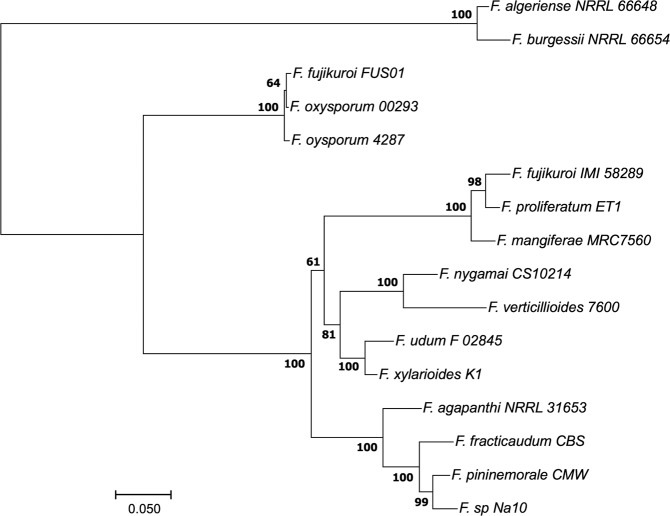
Maximum-likelihood tree of the *carP* sequences listed in Fig. S6. The tree derives from multiple alignment based on structures made with the locARNA program used for the RNAcode test. Branch support analysis was evaluated by 1000 ultrafast bootstrap replicates. The tree indicates that FUS01 is actually a *F. oxysporum* strain, wrongly classified as *F. fujikuroi*.

Taken together, all the data suggest that the *carP* transcript does not have a protein-coding function and, therefore, could play a role as regulatory RNA. The *carP* sequence is too long for reliable 2D predictions, but if its function depends on a conserved secondary conformation, the structure could be inferred from comparison of the orthologs of *carP* in different *Fusarium* species. We have used the RNAcode procedure, capable of detecting possible coding features based on multiple alignment, to obtain a consensus structure. The alignment was performed with the LocARNA-P method^31^, using 15 *carP* sequences (Supplementary Fig. S6 and Table S3), including *Fo*-*carP* and *Ff*-*carP*. The resulting maximum-likelihood tree (Fig. 4) showed that most of the *carP* sequences investigated are closer to *Ff-carP* than *Fo-carP*. The rapid evolution of *carP* makes it possible to build a reliable phylogenetic tree for such closely related species, which contrasts with the lower resolution of the trees based on gene sequences usually used for this purpose, as those of the *Tef1* gene (supplementary Fig. S7).

The finding of a consensus secondary structure (Supplementary Fig. S8) is consistent with a hypothetical structural role but does not prove it. Some lncRNAs act directly on mRNAs of the target genes through pairing, affecting positively or negatively their stability or their translation. A search with the Clustal Omega program on possible target mRNAs, such as those of the *carS* or *wcoA*/*wco1* regulatory genes, or those of the structural genes of carotenogenesis, revealed the absence of sequences of more than 20 bp with more than 50% identity, a threshold below which coincident segments are found in any random gene. This result makes unlikely a direct function of *carP* by pairing on mRNAs of the genes of interest.

### Occurrence of *carP* in other species

NCBI megablast with *Fo*-*carP* versus the nr database of all species, except fungi, did not give significant matches (E < 10). Similarly, no significant match was obtained with an NCBI megablast with *Fo*-*carP* or *Ff*-*carP* versus the nr database restricted to all fungi, except those of the genus *Fusarium*, and the same result was obtained with blastn of *Fo-carP* in the JGI Mycocosm database. Therefore, *carP* is specific to *Fusarium* among the fungi and is not found outside the fungal kingdom. Moreover, the *carP* sequence is widespread but not universal in *Fusarium* species: A blastn search with *Fo-carP* against the 249 *Fusarium* whole genome sequences (WGS) available at NCBI gave only positive hits in 166, using as selection criteria E < 0.001 and query cover >25% (Supplementary Table S4). Therefore, *carP* was not identified in 1/3 of the available *Fusarium* WGS. In contrast, orthologs for the *carRA* and *carB* carotenoid pathway genes, as well as for the *carS* regulatory gene, are found in all of them. However, when *carP* was present, it exhibited a high sequence conservation. Thus, when compared to at least 97% of the *Fo*-*carP* sequence, the percentages of *carP* identity in other *Fusarium* species varied from 68,6% in *Fusarium verticillioides* to 97,1% in *Fusarium solani*.

### Genomic organization of *carP* in relation to *carS* in *Fusarium*

**Figure 5:**
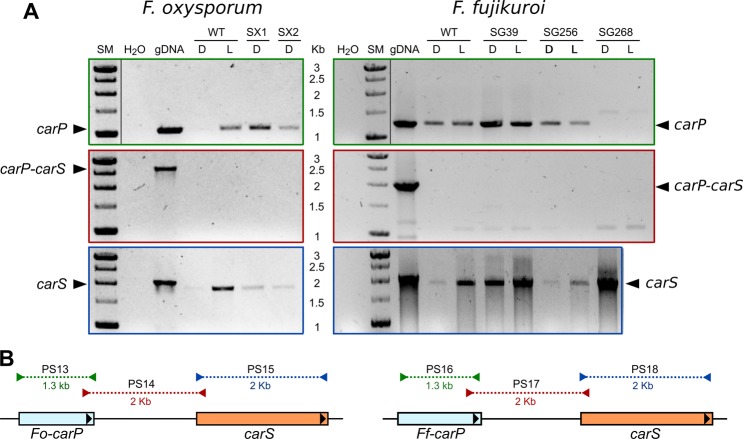
(**A**) PCR verification of *carP* and *carS* as independent transcripts in *F. oxysporum* (left panels) and *F. fujikuroi* (right panels). Amplifications were performed by PCR on cDNA samples in the indicated strains and conditions using primers from internal sequences of *carP* (upper panels), internal sequences of *carS* (lower panels) or connecting internal sequences of *carP* and *carS* (central panels). SM: size markers. H_2_O: test for lack of amplification without template DNA. gDNA: amplification from genomic DNA from the wild strain (positive control), WT: Wild strain. SX1, SX2, and SG39: *carS* mutants. SG256: SG39 complemented with wild *carS*. SG268: mutant lacking the *carP* sequence (Δ*carP*) as an additional negative control in *F. fujikuroi*. Lanes cropped from different positions of the original gels are separated by a black line. Full-length blots/gels are presented in Supplementary Fig. S13. (**B**) Schematic representation of sizes and locations of PCR products obtained with the indicated primers sets (PS) in *F. oxysporum* (left) and *F. fujikuroi* (right).

The proximity of *carP* to *carS* and its transcription on the same strand suggest a functional connection. We revised the information available for *Fusarium* species on prediction of transcripts in the *carS* region through the Browser FungiDB, using *Fusarium oxysporum* f. sp. *lycopersici* 4287 as a reference. The result showed considerable variations in 5′ and 3′-UTR *carS* extensions between different *Fusarium* species, as well as *carS* antisense transcripts from the 3′ extensions of the neighbouring gene in some species (Supplementary Fig. S9). In some cases, the 5′ end of the *carS* transcript extended to the 4-kb upstream region, and at least in the case of *F. verticillioides*, it overlapped with the *carP* sequence. This raises the question of whether *Fo-carP* and *Ff-carP* may be the result of eventual transcription of *carS* from a 5′ distant site, resulting in a long *carP*-containing 5′-UTR. To test this hypothesis, PCR experiments were carried out with combinations of primers from internal regions of *carP* and *carS* to verify their occurrence in single transcripts using cDNA obtained from mycelia of wild type and mutant strains of *F. oxysporum* and *F. fujikuroi*, grown in darkness or under light. PCR amplification between the *carS* and *carP* sequences was obtained from the genomic DNA, but not from the cDNA of any of the strains and species tested, while the amplifications were detected for the internal *carP* or *carS* sequences (Fig. 5). We conclude that *carP* and *carS* derive from separate transcription events, and that *carP* is an independent lncRNA.

### Expression of *carP*

The RNA-seq data displayed in Fig. 1 show that the *carS* mutation results in enhanced transcript levels for *carP* in both *Fusarium* species. However, due to the low content of *carP* RNA in wild strains, the effect of light was not clear. To learn about the regulation of *carP* transcription, the effects of light and *carS* mutation on *carP* RNA levels were investigated by RT-qPCR in different strains and conditions in *F. oxysporum* and *F. fujikuroi*, and the analysis was extended to other relevant genes.

**Figure 6:**
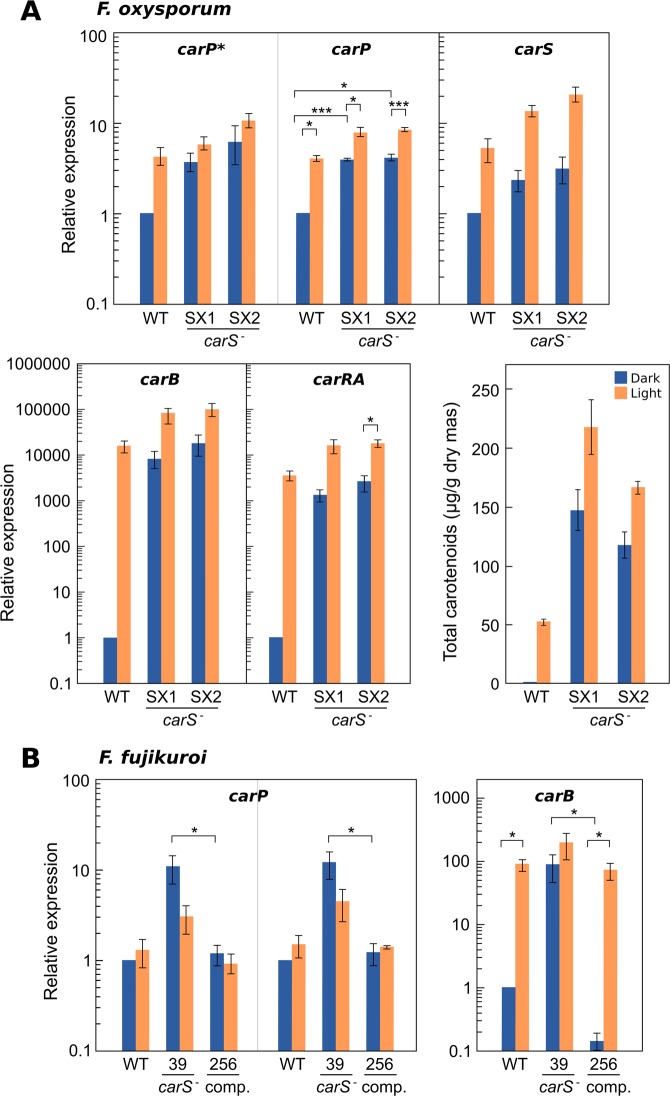
(**A**) Effect of light and *carS* mutation (*carS* mutants SX1 and SX2) on transcript levels for genes *carP*, *carS*, *carRA,* and *carB* (light: 1-hour illumination) and carotenoid content (light: continuous illumination) in *F. oxysporum*. In the case of *carP*, the RT-qPCR primers were selected from two different *carP* regions, one of them contained in the carP* sequence. (**B**) Effect of light (1-hour illumination) or *carS* mutation (*carS* mutant SG39 and *carS* complemented strain SG256) on transcript levels for the *carP* and *carB* genes in *F. fujikuroi*. In both species, for RT-qPCR analyses the strains were grown in DGasn broth for 3 days in the dark (dark bars) and exposed for 1 hour to light (light bars). RT-qPCR data show the mean and standard error of RT-qPCR data from three independent experiments. The relative mRNA levels are referred to the mRNA content of the wild strain in darkness. Differences found to be significant according to the *t* tests are indicated (P-values, *p < 0.033; **p < 0.002; ***p < 0.001).

In wild *F. oxysporum*, transcript levels of *Fo-carP* increased appreciably in light in relation to darkness (Fig. 6A). The result was similar with primers for carP* or for another *carP* sequence, as expected for two regions of the same transcript. Photoinduction was less apparent in the *carS* mutants SX1 and SX2, which exhibited higher levels of *carP* RNA in the dark and after illumination. In contrast, exposure to light produced a similar five-fold increase in *carS* transcript, regardless of the presence of the *carS* mutation. SX1 and SX2 have point mutations in the coding sequence of *carS*^18^, and therefore are expected to contain the complete transcripts of the gene. In the case of SX2, a mutation resulted in a premature stop codon in the *carS* coding sequence that is expected to generate a truncated polypeptide that lacks approximately 50% of the protein. The results with *Fo-carP* were similar to those obtained with the structural genes *carRA* and *carB*, but in this case the effect of light in the wild strain or the effect of the *carS* mutation were much greater. As expected, the carotenoid content correlated with the mRNA levels of the *carRA* and *carB* genes, but with higher carotenoid content in *carS* mutants than in the wild strain under illumination.

The expression pattern of *carP* in *F. fujikuroi* differed from that of *F. oxysporum* (Fig. 6B), a result already suggested by the comparison of their RNA-seq data (Fig. 1). *Ff*-*carP* RNA levels were hardly affected by light in the wild strain investigated. In this case, to test the effect of the *carS* mutation, the study focused on a *carS* mutant, SG39, and its *carS*-complemented strain SG256, in which a functional *carS* gene has been reintroduced. *carP* transcript levels were higher in the *carS* mutant than in the strains with a functional *carS* gene, especially without illumination.

### Phenotype of a Δ*carP* and ΔcarP* mutants in *F. oxysporum*

**Figure 7:**
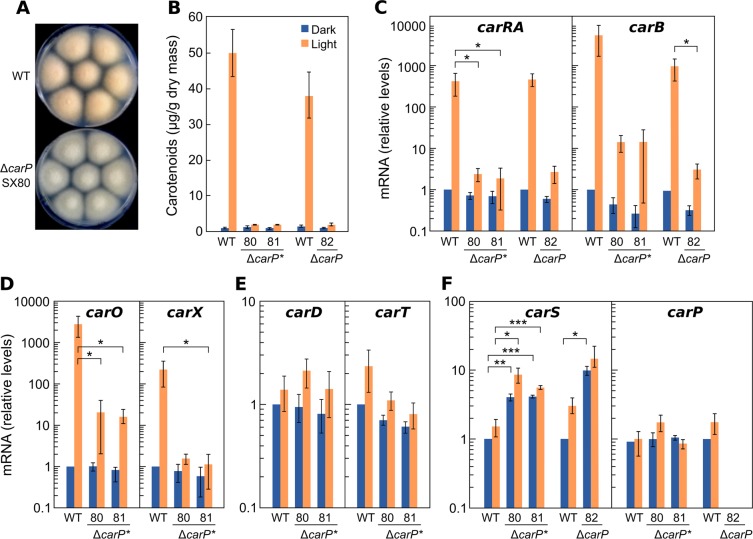
Effect of *carP* deletion in *F. oxysporum*. (**A**) Aspect of surface colonies of the wild strain and a representative Δ*carP* mutant grown on minimal medium for 1 week under light. (**B**) Carotenoid content in the wild strain and mutants ΔcarP* and Δ*carP* grown for 1 week in the dark or under light. (**C–F**) Transcript levels for the *carRA*, *carB, carO, carX, carD, carT, carS*, and *carP* genes in the same strains grown in the dark or exposed for 1 hour to light. RT-qPCR data show the mean and standard error of three independent experiments. Relative mRNA levels refer to the mRNA content of the wild strain in the dark. Differences found to be significant according to the *t* tests are indicated (P-values, *p < 0.033; **p < 0.002; ***p < 0.001).

To determine the function of *carP* in *F. oxysporum*, a Δ*carP* mutant (SX82) and two ΔcarP* mutants (SX80 and SX81) were obtained. All three mutants had the same morphology and growth rate as the wild strain. However, they exhibited albino mycelia under illumination (SX80 shown as an example in Fig. 7A), consistent with a defect in carotenoid production, while presenting a wild-type appearance in the dark. As expected, carotenoid analyses showed a strong photoinduction of the carotenoid content in the wild strain, but only traces of carotenoids in the three mutants in the light, with levels only slightly higher than those in the dark (Fig. 7B).

To determine whether the phenotype in the light was due to reduced expression of the structural *car* genes, their mRNA levels were quantified by RT-qPCR in the dark or after one hour of illumination and compared with those of the wild strain. The results showed a sharp decrease in the transcript levels of *carRA* and *carB* genes in the Δ*carP* mutants compared to the wild strain (Fig. 7C). Such descent was similar in the deletion strains Δ*carP* and ΔcarP*, indicating that the absence of carP* was sufficient for the total loss of *Fo-carP* function. However, although the mutants exhibited a 100-fold decrease in the mRNA levels for these genes, a minor photoinduction was still apparent compared to the levels in the dark, which was barely reflected in the actual carotenoid content in the light. On the other hand, the mRNA content of *carRA* and *carB* did not suffer significant variations in the dark compared to those of the wild strain, indicating the participation of *Fo-carP* in the transcriptional photoinduction of the structural *car* genes in *F. oxysporum*.

The deletion of *carP* also produced clear reductions in the photoinduction of the *carX* and *carO* genes (Fig. 7D), organized in a coregulated cluster with *carRA* and *carB* (Supplementary Fig. S1). The effect on *carO* was similar to that of *carB*, adjacent in the *car* cluster, and the effect on *carX* was similar to that of *carRA*, transcribed divergently from a common regulatory region. In contrast, the effect of *carP* deletion was hardly apparent for the *carT* and *carD* genes (Fig. 7E), involved in the last steps of NX production. This is consistent with the lower photoinduction exhibited by these genes compared to *carRA* and *carB*, which may explain the poor influence of *carP* deletion. Interestingly, the transcript levels of the *carS* gene increased appreciably in the Δ*carP* and ΔcarP* mutants, in the dark or after illumination (Fig. 7F). However, the ΔcarP* mutation did not affect the levels of the transcription of *carP* itself.

### Phenotype of Δ*carP* mutants in *F. fujikuroi*

**Figure 8:**
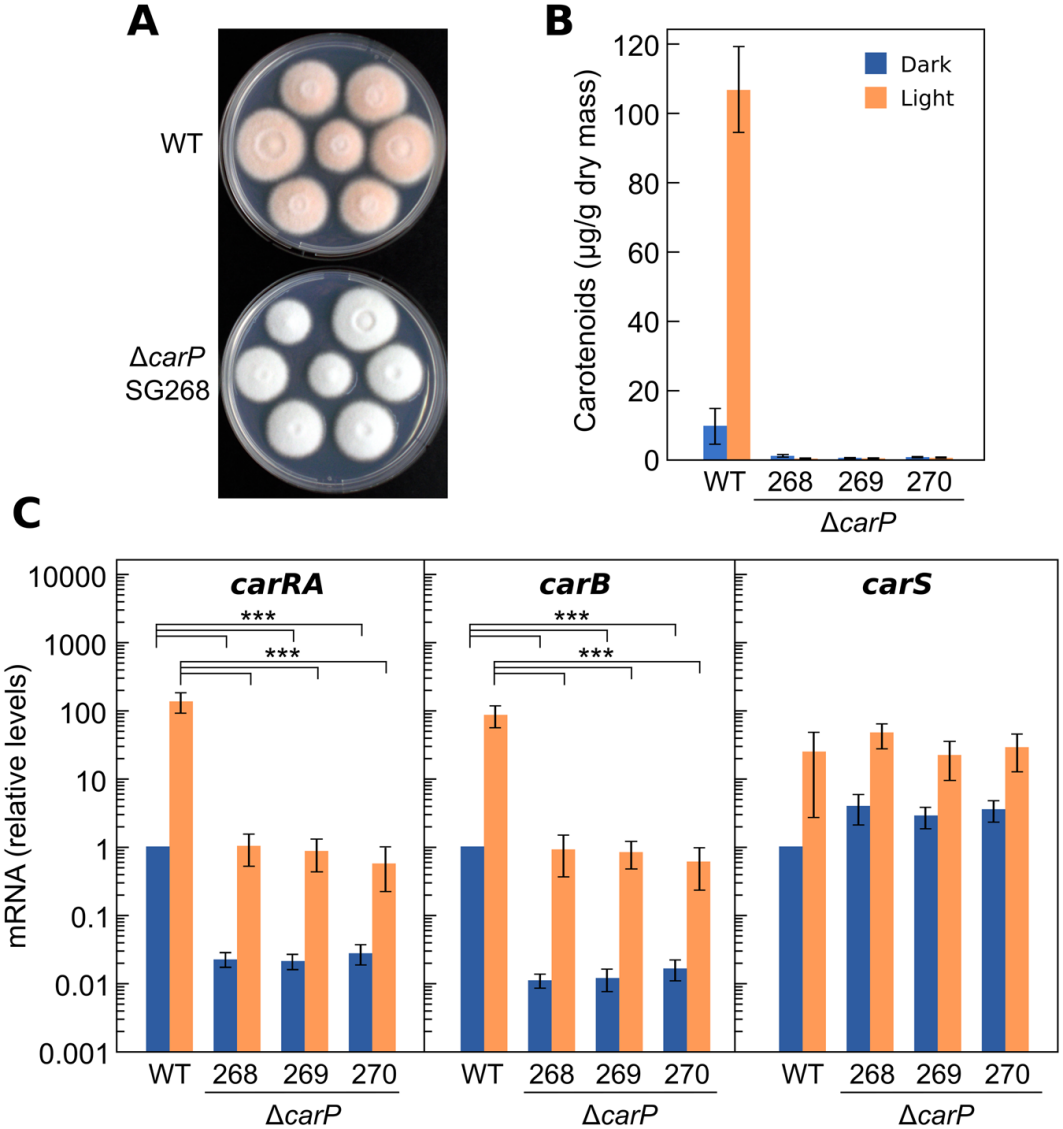
Effect of *carP* deletion in *F. fujikuroi*. (**A**) Aspect of surface colonies of the wild strain and a representative Δ*carP* mutant grown on minimal medium for 1 week under light. (**B**) Carotenoid content in the wild strain and three Δ*carP* mutants grown for 1 week in the dark or under light. (**C**) Transcript levels for the *carRA*, *carB*, and *carS* genes in the same strains grown in the dark or exposed for 1 hour to light. RT-qPCR data show the mean and standard error of three independent experiments. Relative mRNA levels refer to the mRNA content of the wild strain in darkness. Differences found to be significant according to the *t* tests are indicated (P-values, *p < 0.033; **p < 0.002; ***p < 0.001).

To determine the function of the *carP* gene in *F. fujikuroi*, three Δ*carP* strains (SG268, SG269, and SG270) were obtained. As already observed in *F. oxysporum*, the colonies of the three Δ*Ff*-*carP* strains exhibited morphologies and growth capacities similar to those of the wild strain but lacked the characteristic orange pigmentation in the light (SG268 shown in Fig. 8A). However, analysis of their carotenoid contents revealed a more drastic effect on carotenogenesis than that observed in *F. oxysporum*. The wild strain of *F. fujikuroi* had detectable amounts of carotenoids in the dark, which increased approximately tenfold under illumination, but the Δ*Ff*-*carP* mutants contained only traces of carotenoids either in the light or in darkness (Fig. 8B). However, mRNA levels for the *carRA* and *carB* genes decreased about 100-fold but exhibited the same pattern of photoinduction. Interestingly, the Δ*Ff*-*carP* mutants contained in the light the same amounts of *carRA* and *carB* transcripts as the wild strain in the dark, but despite that, their carotenoid content was much lower. In contrast to the effect on *carRA* and *carB*, the Δ*Ff*-*carP* mutants exhibited a modest increase on *carS* mRNA levels in the dark, which was not apparent after illumination.

### Effect of *carP* deletion on the expression of photoreceptor genes

As already mentioned, the regulation of carotenogenesis by light is mediated in *F. fujikuroi* by the WcoA photoreceptor, called Wc1 in *F. oxysporum*^32^, with the accessory participation of the CryD and VvdA photoreceptors^16^. For that reason, we investigated whether the reduced photoinduction of *car* structural genes as a result of the deletion of *carP* is due to an alteration in expression of the genes for these photoreceptors. The study of mRNA levels of the *wcoA*/*wc1*, *cryD*, and *vvdA* genes by RT-qPCR in the wild strain and Δ*carP* mutants showed the same expression patterns for the three genes in *F. fujikuroi* and *F. oxysporum* (Supplementary Fig. S10). The transcript levels of the *cryD* and *vvdA* genes were strongly photoinduced, but this photoinduction was maintained in the Δ*carP* mutants, indicating that *carP* is not necessary for this photoresponse. As additional evidence, the effect of *carP* deletion was also checked in the *F. oxysporum* FOXG_01269 gene, ortholog of the photoregulated *con-10* gene of *N. crassa*^33^. FOXG_01269 maintained a robust photoinduction in the Δ*carP* mutant, indicating that *carP* is not a general regulator of photoinducible genes in *Fusarium*.

## Discussion

Most studies on lncRNAs have been dedicated to higher eukaryotes, especially mammals, but they are present in all taxonomic groups, including plants^34^ and microorganisms, such as the yeasts *Saccharomyces cerevisiae* and *Schizosaccharomyces pombe*^35^. The occurrence of lncRNAs has also been reported in some filamentous fungi. In *N. crassa*, a study of massive RNA sequencing revealed 939 lncRNAs, more than half of them antisense of annotated genes^36^. In *Fusarium graminearum*, a search identified 2574 lncRNAs, of which 1040 were antisense transcripts, and 547 exhibited differential expression associated to the formation of fruiting bodies^37^. *F. oxysporum* induces changes in the formation of lncRNAs by plants during the infection process as a defence mechanism^38,39^, but the presumable changes in lncRNA production in the fungus associated to pathogenesis have not been investigated.

To date, only one lncRNA has been characterized in filamentous fungi, *HAX1* of *T. reesei*^40^, which plays a regulatory role in the expression of cellulases, and possibly other lytic enzymes. The *HAX1* gene was identified from phenotypic alterations resulting from insertional mutants, and its function was confirmed by targeted mutation and overexpression, but its mechanism of action remains unknown. In this work, we have demonstrated for the first time the participation of a *Fusarium* lncRNA, that we called *carP*, in a specific metabolic function. The lack of translation of the ORFs in the lncRNAs is difficult to prove. In our case, the lack of correlation between ORFs in two *carP* versions investigated: *Fo-carP* in *F. oxysporum* and *Ff-carP* in *F. fujikuroi*, whose deletions produce similar phenotypes, is a strong indication of the non-participation of a protein in the regulatory function of *carP*. This lack of correlation is basically due to the occurrence of gains or losses of few bases, as revealed by the alignment of *Fo-carP* and *Ff-carP*, which indicates that their sequences have evolved maintaining their function despite the alteration of their ORFs. Accordingly, none of the various informatic tools used to investigate its sequence found any indication of protein coding functions either in *Fo-carP* or *Ff-carP*. Considered globally, the available information strongly supports the non-coding nature of *carP* in *Fusarium*.

The absence of relevant *carP* matching sequences in predictable target genes suggests that *carP* does not exert its function by modulating the translation or stability of mRNAs involved in carotenogenesis. Other types of mechanisms, such as those based on a specific three-dimensional conformation of the transcript, are not ruled out. *carP* RNA could interfere with the function of a regulatory protein, which could be CarS. E.g., *carP* RNA could bind CarS and block its repressor function, so that the absence of *carP* would leave CarS free to suppress carotenoid synthesis. In this case, the light could perform its activating function, at least partially, facilitating the binding of *carP* to CarS. As an example of protein inactivation by binding of a lncRNA, the 600-nt *gas5* ncRNA binds to the DNA-binding domain of the glucocorticoid receptor under nutrient starvation and inhibits glucocorticoid-regulated transcription in human cells^41^. Other mechanisms of action based on *carP* interaction with regulatory proteins cannot be ruled out. E.g., *carP* might act as a scaffold to facilitate the assembly of histone modification enzymes and alter the expression of target genes. This is the case of the human HOTAIR lncRNA, which binds to two independent protein complexes and results in histone H3 lysine 27 methylation and lysine 4 demethylation^42^.

A possible regulatory mechanism of lncRNAs is the interference of its transcription on the start of transcription of a neighbor gene from a downstream promoter, resulting in a down-regulation. A well-known example is the regulation of the *SER3* gene by the upstream *SRG1* ncRNA in *S. cerevisiae*^43^. This could be the case of the *carP* gene, whose transcription could interfere negatively with the transcription of *carS*. The increase in *carS* mRNA levels found in the Δ*Fo-carP* mutants is consistent with this hypothesis. However, this effect is not so evident in the Δ*Ff-carP* mutants, which again suggests a different mechanism of action between *carP* RNAs in both species. Moreover, the ΔFo-carP* deletion affects only the 3′ segment of *carP*, but the phenotype resulting of this mutation is very similar to the elimination of the entire *carP* segment. Approximately 56% of the *carP* sequence (659 of 1178 bp) is conserved in the ΔFo-carP* mutant. This should be interpreted as that the 3′ end region of *carP* plays an essential role in its function, but it does not mean that the rest of the transcript is not essential, as would happen if the function depended on the overall structure of the RNA.

The orientation of the *carP* transcript in the upstream region of *carS* in *Fusarium* species suggests an evolutionary origin from a long 5′-UTR region of the *carS* gene. This hypothesis is supported by the finding of the *carP* sequence in a single transcript with *carS* in *F. verticillioides*. 5′-UTRs are usually shorter than 3′-UTRs in eukaryotic transcripts, even in higher eukaryotes^44^. The length of the 5′-UTR of the *carS* gene in *F. verticillioides* would be very exceptional compared to usual 5′-UTR lengths. It should also be noted that, according to RNA-seq data, the levels of *Fo-carP* and *Ff-carP* are very low, and different from those of *carS*. This indicates a low transcription of *carP* or a very short life for this lncRNA. However, the levels of *Fo-carP* and *Ff-carP* are higher in *carS* mutants than in wild strains, suggesting a regulatory loop between both genes. This could be consistent with a direct role of *carP* on the CarS protein, that could result in the accumulation of *carP* in the absence of a functional CarS polypeptide.

Despite the functional similarity between *Fo-carP* and *Ff-carP*, there are some discrepancies between them that suggest differences in their mechanisms of action. RT-qPCR analyses show that in wild strains, *Fo-carP* is apparently induced by light, but *Ff-carP* is not. On the other hand, the Δ*Fo-carP* mutants have lost the photoinduction of carotenoid synthesis, although they retain a slight photoinduction of the structural *car* genes, whereas the Δ*Ff-carP* mutants show a clear decay in carotenoid synthesis in both light and in darkness and a drastic drop in mRNA levels of structural *car* genes, but they maintain a more clear photoinduction. Δ*carP* phenotypes indicate that *carP* is necessary for high expression of *car* genes in both species, but its influence on photoinduction is only partial in *F. oxysporum*, and almost non-existent in *F. fujikuroi*. This is consistent with the lack of effect of the deletion of *carP* on the expression of the genes involved in the photoreceptor machinery, either in *F. oxysporum* or in *F. fujikuroi*. In summary, the characteristics described for *carP* conform to the requirements to be considered as a lncRNA that acts as an up-regulatory element in *Fusarium* carotenogenesis. Its mechanism of action is unknown, and it will be the focus of future work.

## Methods

### Strains and culture conditions

Wild strain of *Fusarium oxysporum* f. sp. *lycopersici* 4287 (race 2) was kindly provided by A. Di Pietro (Universidad de Córdoba, Spain), and *carS* mutants SX1 and SX2 were obtained from strain 4287 by chemical mutagenesis^18^. Wild strain of *Fusarium fujikuroi* IMI58289 was obtained from the Imperial Mycological Institute (Kew, Surrey, England), and the *carS* mutant SG39 was obtained from IMI58289 by chemical mutagenesis^17^. The *carS*-complemented strain SG256 was obtained through introduction of a wild *carS* allele in SG39^19^ and subsequent loss of the mutant allele^20^.

For routine maintenance and to obtain conidia, *F. oxysporum* strains were grown on PDB medium, either prepared in the laboratory (200 g potatoes were peeled, sliced, boiled in 1 l of distilled water for 60 minutes, and filtered. 20 g of glucose were added, and the volume was completed with distilled water to 1 l) or commercially obtained (24 g/l of PDB, FORMEDIUM LTD, 16 g/l agar). To obtain conidia, the strains were grown for 3–4 days with shaking at 200 rpm at 30 °C in 250 ml Erlenmeyer flasks with 100 ml of culture medium, inoculated with 10^8^ conidia. The conidia were collected through a filter (Monodur 10–15 μm) and used immediately or kept frozen in 30% glycerol at −80 °C. For expression studies the strains were grown on DGasn minimal medium, containing 30 g/l glucose, 3 g/l asparagine, 1 g/l KH_2_PO_4_, 0.5 g/l KCl, 0.5 g/l MgSO_4_·7H_2_O, and microelements (adapted from DG medium^45^).

Because of their lower conidiation levels, conidia of *F. fujikuroi* strains were obtained after growth on sporulation agar, consisting of 1 g/l glucose, 1 g/l yeast extract, 1 g/l NO_3_NH_4_, 1 g/l KH_2_PO_4_, 0.5 g/l MgSO_4_·7H_2_O, 16 g agar. To collect the conidia, the culture surface was washed with water, the mycelial debris was removed by filtration, and the resulting conidia were counted in a haemocytometer (Bürker chamber, Blau Brand, Germany). For phenotypic characterizations and expression studies, *F. fujikuroi* strains were grown on DG medium, identical to DGasn medium but with 3 g/l NaNO_3_ instead of asparagine^45^.

For expression studies, in the case of *F. oxysporum*, Petri dishes of 15 cm diameter with 80 ml DGasn liquid medium were inoculated with 10^6^ fresh conidia of the corresponding strains and incubated in the dark for three days. At this time, they were eventually exposed to light for one hour. In the case of *F. fujikuroi*, 500-ml Erlenmeyer flasks with 100 ml of DG medium were inoculated with 10^6^ conidia of the corresponding strains and cultured in the dark for three days on an orbital shaker at 150 rpm. Subsequently, 25-ml samples were transferred to standard Petri dishes under red safelight and incubated for one hour in the dark or under white light. In both species, light exposures were performed under a platform with 4 fluorescent tubes (Philips TL-D 18 W/840) at a distance of *ca*. 60 cm, providing a light intensity of 7 w/m^2^ (420 Lm/w). Upon incubation, the mycelium samples were removed by filtration, washed with distilled water, frozen in liquid nitrogen, and stored at −80 °C.

For analysis of carotenoid production, standard 9-cm diameter Petri dishes with 25 ml solid medium were incubated at 30 °C in the dark or under light as described above. Each Petri dish was inoculated with 7 symmetrically distributed punctures with sterile sticks previously punctured on isolated fresh colonies.

### PCR assays and determination of *carP* transcript orientation

Depending on the purpose of the experiment, different thermostable polymerases were used for PCR reactions: BIOTAQ^TM^ DNA polymerase (Bioline, Memphis, TN, U.S.A.) for PCR checks, Expand High Fidelity PCR System (Roche, Mannheim, Germany) for vector construction, and Velocity DNA polymerase (Bioline) for expected products larger than 5 kb. Reaction conditions were those indicated in manufacturer’s instructions. The reactions were carried out in total volumes of 25 µl with an amount of template DNA ranging from 10 to 50 ng for genomic DNA and from 1 to 10 ng for plasmid DNA. The sets of primers used are described in Supplementary Table S5.

To analyse the transcription orientation of the *carP* gene, a variant of the cDNA synthesis protocol was used. DNase-treated RNA was reverse-transcribed with the Transcriptor First Strand cDNA synthesis kit (Roche), following the manufacturer’s instructions. The polyT primer, normally used in the synthesis of cDNA, was replaced with a mixture of specific primers for each of the chains, so that only the cDNA substrate corresponding to the orientation of the transcript was obtained. Each primer mix consists of 3–4 different primers, described for each species of *Fusarium* in Supplementary Table S5, which cover different parts of the transcript in a single orientation. Subsequently, a PCR was performed for the *carP* gene with both substrates (primer sets 3 and 9) and it was only expected that the PCR product would be formed from the substrate generated by the mixture of primers complementary to the orientation of the transcript. As a control, the same analysis was performed in parallel with the β-tubulin gene in each species (primers 4, 5, 10, and 11, and primer sets 6 and 12).

### Plasmid constructions and transformation

Plasmids for deletion of target sequences in *F. oxysporum* and *F. fujikuroi* were constructed by homologous recombination of four DNA segments, with overlapping end-sequences of at least 20 bases. One DNA segment was the 5.7-kb yeast vector pRS426 (Fungal Genetics Stock Center^46^), which contains the *URA3* gene of *S. cerevisiae*, digested with *Xho*I and *Eco*RI at its multicloning site. Another DNA segment was the hyg^R^ cassette, which contains the *hph* gene of *E. coli* under control of regulatory sequences of *Aspergillus nidulans*, obtained by PCR from plasmid pCSN44 (Fungal Genetics Stock Center) with primer set 19. Finally, two DNA segments were obtained on both sides of the target sequence of *carP* by PCR using primers with tails overlapping with the ends of *Xho*I/*Eco*RI digested pRS426 on one side, and with the ends of the Hyg^R^ resistance cassette on the other side. The four fragments were introduced by transformation into the strain FY834 of *S. cerevisiae* (*MAT*α, *ura3-52*, *leu2Δ1*, *trp1Δ63*, *his3Δ200*, *lys2Δ202*), where they recombined through the coinciding end-sequences to generate circular plasmids.

Using this method, the 8.9-kb plasmid pDul8 was obtained through the joining of the linear pRS426 and Hyg^R^ cassette segments with a DNA fragment upstream to carP*, obtained with primer set 23, and with a DNA fragment downstream of carP*, obtained with primer set 24. Similarly, the 9.8-kb plasmid pDul14 was obtained by joining the linear pRS426 and Hyg^R^ cassette segments with a DNA fragment upstream to the entire *Fo-carP* sequence, obtained with primer set 20; and with a DNA fragment downstream of *Fo-carP*, obtained with primer set 21. pDul8 and pDul14 were used to generate the carP* and *carP* deletions in *F. oxysporum*, respectively. The same procedure was followed to obtain the 9.3-kB plasmid pcarPhyg, used to obtain the *carP* deletion in *F. fujikuroi*. In this case, the DNA segments corresponding to the pRS426 and the Hyg^R^ cassette were joined with DNA fragments upstream (obtained with primer set 26) and downstream (primer set 27) to *Ff-carP*. Transformations were achieved with linear replacement cassettes obtained from plasmids pDul14, pDul8, and pcarHyg with primers sets 22, 25, and 28, respectively. Sizes of the 5′ and 3′ *carP* border regions in the replacement cassettes were (bp): 1429 (5′) and 1508 (3′) for *carP*, and 1193 (5′) and 790 (3′) for carP* in *F. oxysporum*, and 1175 (5′) and 1257 (3′) for *carP* in *F. fujikuroi*.

Protoplasts of the wild strain of *F. fujikuroi* were obtained and transformed as described^47^. The same protocol was used for the wild strain *of F. oxysporum*, except that for the formation of protoplast 5 × 10^8^ spores were inoculated into 100 ml YPED-2G (3 g/l yeast extract, 10 g/l peptone, 20 g/l glucose) and incubated for 10–12 hours at 22 °C. Germlings were collected by filtration, washed with 0.7 M NaCl, and transferred to 15 ml of an enzyme solution in 0.7 M NaCl containing 13 g/l of lysing enzymes of *Trichoderma harzianum* (Sigma-Aldrich, St. Louis, MO, USA), 33 g/l of driselase from basidiomycetes (Sigma-Aldrich) and 60 mg/l of chitinase from *Streptomyces griseus* (Sigma-Aldrich). The enzymatic treatment lasted between 30 minutes and 1 hour until protoplast formation.

### Generation of Δ*carP* and ΔcarP* mutants in *F. oxysporum*

To obtain Δ*Fo-carP* mutant strains, wild protoplasts were transformed with the linear replacement cassette derived from plasmid pDul14. Five transformants were obtained by selection on hygromycin-supplemented medium and, after three selection steps through uninucleate spores to ensure homokaryosis, they were analysed by PCR using primers external to the deleted region. Only one of the 5 transformants (#3) gave the 4.4 kb band corresponding to the replacement of *carP* with the Hyg^R^ cassette, the same size of the DNA product obtained with the plasmid pDul14 (Supplementary Fig. S11A). In the other 4 transformants, a 4.2 kb band similar to that found in the wild strain and corresponding to the intact *Fo*-*carP* sequence was obtained, indicating heterologous integrations of the plasmid. As an additional verification, a Southern blot hybridization of genomic DNA from the wild strain and transformant #3 was performed (Supplementary Fig. S11B) using as a probe a DNA segment next to the deleted region (Supplementary Fig. S11C). Genomic DNA samples were digested with *Ava*I restriction enzyme, for which there is a target within the *carP* gene, but not in the Hyg^R^ cassette. The result showed a 3.3-kb band in the transformant, indicating the presence of the Hyg^R^ cassette instead of *Fo*-*carP*, and a 1.8 kb band in the wild strain, as expected from the presence of the *Fo*-*carP* sequence. This Δ*carP* transformant was named SX82.

In addition, Δ*Fo*-carP* mutants were obtained by transformation of wild protoplasts with the linear replacement cassette obtained from plasmid pDul8. As a result, eight transformants were obtained, passed three times through uninucleate spores, and checked by PCR with primers surrounding the replaced region. Five transformants (#2, #3, #4, #5, #8) showed the expected 2.7 kb band instead of 2 kb of the wild strain, indicating the replacement of carP* by the Hyg^R^ cassette (Supplementary Fig. S11D). Two positive transformants were chosen for verification by Southern blot using as a probe a DNA segment adjacent to the deleted carP* region (Supplementary Fig. S11C). This hybridization gave a 4-kb band in the case of the two transformants analysed (#3 and #4), consistent with the replacement of carP* by the Hyg^R^ cassette, compared to the 1.8 kb band obtained with the wild strain (Supplementary Fig. S11E). These ΔcarP* transformants were called SX80 and SX81.

### Generation of Δ*carP* mutants in *F. fujikuroi*

Wild protoplasts were transformed with the linear replacement cassette obtained from plasmid pcarPhyg, and twenty transformants were obtained upon selection on hygromycin-supplemented medium. After three passes through uninucleate spores, the replacement of the *carP* sequence by the Hyg^R^ cassette in the candidate transformants was analyzed by PCR using different primer combinations (Supplementary Fig. S12A). In eleven of the transformants, a 3.7 kb band was obtained with primer set 33, corresponding to an amplification from a sequence external to *carP* and absent in pcarPhyg, and a terminal Hyg^R^ sequence, indicating the desired loss of the *carP* sequence (positive transformants #1, #2, #4, and #10 shown in Supplementary Fig. S12B). Southern blot hybridization of genomic DNA from the wild strain, three of the positive transformants (#1, #2, and #4) and one negative (#6), was carried out using as a probe a DNA segment near the deleted region (Supplementary Fig. S12A). Genomic DNA samples were digested with restriction enzymes *Xho*I and *Eco*RI, chosen for their opposite presence in the *carP* gene and in the Hyg^R^ cassette. The results in the *Eco*RI*-*digested samples showed a 4-kb band in the wild strain and in the negative transformant, and the expected 1.9-kb band in the positive transformants (Supplementary Fig. S12C). In samples digested with *Xho*I, the 4.9-kb band corresponding to the presence of the Hyg^R^ cassette was found in the positive transformants, while the expected 3.1-kb band for the *carP* sequence was found in the wild strain and in the negative transformant, confirming the results of the PCR tests and the hybridization of the samples digested with *Eco*RI. Therefore, the transformants #1, #2, and #4 had lost the *Ff*-*carP* sequence and were chosen for detailed phenotypic characterization. These strains were called SG268, SG269, and SG270, respectively.

### Southern blot of transformants

Southern blot analyses were achieved essentially as described^48^. Samples of at least 10 μg of genomic DNA were treated with restriction enzymes and the resulting products were electrophoresed on a 0.7% agarose gel and subjected to the Southern protocol using a positively charged nylon membrane (Hybond-N from Amersham). The probes used in *F. oxysporum* were obtained from genomic DNA sequences by PCR with primer set 30 in the case of the southern for Δ*carP* mutants, and primer set 32 in the case of the southern for ΔcarP* mutants. The probe used in the southern of the *F. fujikuroi* transformants was obtained with primer set 37. The probes were labelled with ^32^P dCTP and the membranes were exposed for radioactivity detection in a radioisotope imaging system (FujiFilm FLA 5100, Life Science, Cambridge, MA, USA).

### Expression analysis

Transcript levels for the genes of interest were determined by reverse transcription qPCR (RT-qPCR) with a LightCycler 480 real-time equipment (Roche). Total RNA samples were extracted with the RNeasy RNA isolation kit (Qiagen, Chatsworth, CA, USA). RNA concentrations were determined with a Nanodrop ND-1000 spectrophotometer (Nanodrop Technologies, Wilmington, DE, USA). Samples of 2.5 μg of RNA were retrotranscribed to cDNA with Transcriptor first-strand cDNA synthesis kit (Roche), and final cDNA concentrations were adjusted to 25 ng/μl. LightCycler 480 SYBR Green I Master (Roche) was used for amplification and detection following the manufacturer’s protocols. Genes and primer sets (forward *vs* reverse in 5′- >3′ orientation) for *F. oxysporum* genes *carP*, *carS*, *carRA*, *carB*, *carO*, *carX*, *carD*, *carT*, *cryD*, *vvdA*, *wc1*, and the *con-10* ortholog FOXG_01269, and for *F. fujikuroi* genes *carP*, *carS*, *carRA*, *carB, cryD, vvdA, and wcoA*, are listed in Supplementary Table S5E (primers sets 38–50 and 52–59). Transcript levels were normalized against the β-tubulin gene FOXG_06228 of *F. oxysporum* (primer set 51) and FFUJ_04397 of *F. fujikuroi* (primer set 61). In some cases, the glyceraldehyde 3-phosphate dehydrogenase (GAPDH) gene *gpdh1*, FFUJ_13490, was used as a second reference gene in *F. fujikuroi* (primer set 60).

Statistical significance of differences between mRNA values was checked with the Welch’s *t* test using the GraphPad QuickCalcs program (www.graphpad.com/quickcalcs/contMenu). Since data were relativized to the value of the wild strain grown in the dark, tests against these values were carried out with the one sample *t* test.

### Bioinformatic analyses

The identification of ORFs in *carP* was carried out with ORFfinder^49^ and the detection of potential protein domains was performed with InterproScan^50^. Clustal alignments were done through the EMBL/EBI server (https://www.ebi.ac.uk/Tools/msa/clustalo). We used different approaches to check the coding capability of *carP*. Three-base periodicity was checked through TestCode statistics^51^ with the Tcode program of EMBOSS^52^, using a window of 200 and a step of 3. Codon usage statistics was plotted with the program Syco of EMBOSS^52^ using a codon usage table generated by the Cusp program of EMBOSS^52^ from the *F. oxysporum* CDSs. The protein-coding potential of *carP* was also analysed using the CPC^28^ and CPC2^29^ algorithms. Coding capability can be also detected by evolutionary conservation. For that, we first made a structure-based alignment of *carP* homologous sequences with LocARNA^31^. Then we studied coding capability by evolutionary conservation with RNAcode^30^.

For the prediction of secondary structure for *carP*, we used LocARNA^31^ plotted with VARNA^53^. Maximum-likelihood trees were generated with the IQ-Tree server^54^. ModelFinder, implemented at the IQ-Tree server, was used to find the free rate heterogeneity substitution model that best fit the alignments^55^. Maximum-likelihood tree of *carP* was generated with the *carP* sequences shown in Fig. S6. The tree derives from the multiple alignment based on the structures carried out with the locARNA program used for the RNAcode test. The maximum-likelihood tree for species phylogeny was based on the translation elongation factor 1-alpha (TEF1). TEF1 was selected as species barcoding gene because rRNAs of the studied species are very similar and ITSs showed intrastrain sequence heterogeneity. TEF1 has proven to be one of the best barcoding genes in fungi^56^. Multiple alignment was performed with ProbCons^57^. In both trees, branch support analysis was evaluated by 1000 ultrafast bootstrap replicates.

## Supplementary information

Supplementary Information.
