# Supplementary material for: A novel lncRNA as a positive regulator of carotenoid biosynthesis in Fusarium
Source: Sci Rep. 2020 Jan 20;10:678. doi: 10.1038/s41598-020-57529-2 (PMC6971296; doi:10.1038/s41598-020-57529-2)
Supplement: Supplementary file 1 — Supplementary Information. [file 41598_2020_57529_MOESM1_ESM.pdf]

A novel lncRNA as a positive regulator of carotenoid biosynthesis in *Fusarium*

Obdulia Parra-Rivero, Javier Pardo-Medina, Gabriel Gutiérrez, M. Carmen Limón, Javier Avalos

---

SUPPLEMENTARY MATERIAL

Figure S1. Carotenoid metabolism in *Fusarium*. Genes and enzymes responsible for each of the biosynthetic steps are indicated with different colours. Genomic organization of the genes is shown in the box. The *carX*/*carRA*/*carB* cluster includes the gene *carO*, encoding a rhodopsin assumed to use retinal as light-absorbing prosthetic group.

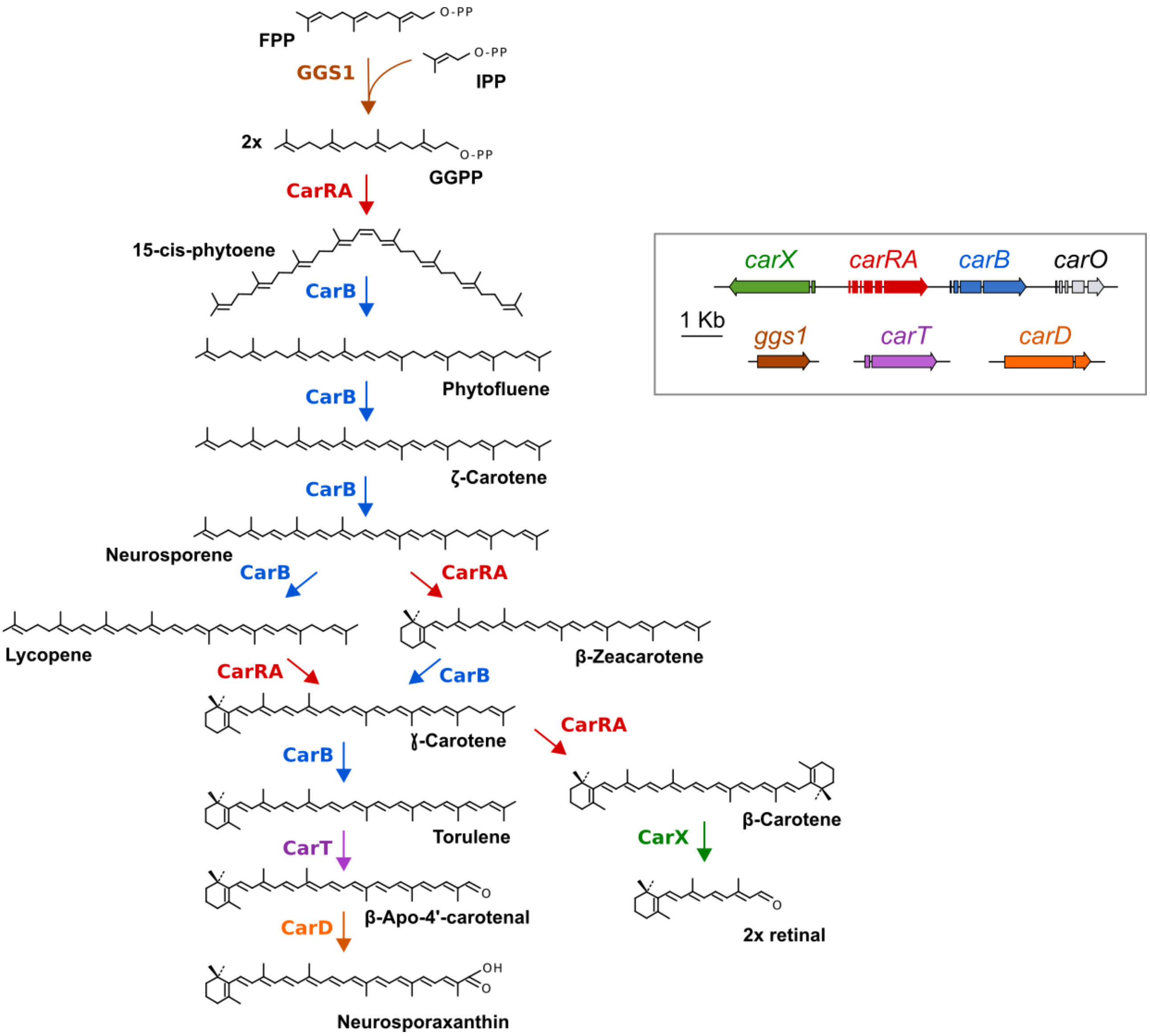

Figure S2. (**A**). Sequences of the *carP* transcripts in *F. oxysporum* and *F. fujikuroi*. Overlapping sequences in both species are indicated in black, and non-overlapping in red. (**B**). Clustal alignment between *F. oxysporum* (Fo-carP) and *F. fujikuroi* (Ff-carP) *carP* sequences.

A

carP F. oxysporum
1357 bp (1165 overlapping in *F. oxysporum carP* sequence)

CTCTCAACTATTAAGTAGCGTCGATGTGCCAGTTGATTCACCCGATGGCCGTTGCAACTGCAGCGTTGTCTC
GATCGTCCTCGTCAGCCAGAATCTCGAAAGGAAAAAGAGTCGAGAGAGAAGCGAGAACGACACCCCTTACGA
TAACCCCCGACTCACAACGGATTTTAGGTGCATTTTCGAGCGCGCGCTGTATTCCGTTTTCTGCGGACCGTC
CAAGAGTGACAAGAGGAGGTTGTACACACAAACAAGAGCAAGATCGGCGACAGATGCGAAACTAGGGTCCTC
TGCCGCGGGGAGAAAGGTCTTGATCGAGTACCGTCTACAATACTGCATCTTCACTGAGAACCCTGCATTCTC
GTTGGTTTCCTGCATCGCGATCTGTGATACTGGTCTCTATGGTTATTGGCCATGCTTGCGCTTCTGGAGGGC
AACTTTTCGGTTACGCTATGAAGAAGGGGAAAGTTTCCTTTGTCTGCCTTGTCTCAATCCTCAGCTCAGTCA
AGAGGCCCCGGCCCAGATCTTGAATCTTGGATCTTGGATCTGAACGTTTTTCTTGGCCCCATTGAGCTGGGA
TGTGTTTTATTTTAGGTTCATCCTCCCATGCATCCCCGGTTGGCAGCACAGCGCACCACAAAACAGCAAAAG
CAGGGAACTGACGAGCTCCCGATTATCGAGATGAGTGAGGGGTTTAGGCTCCGTCCCTCGGTTGCTGCAACG
GGCACCAAAGTCTATCTATCTGGTGCTGCACCTTGGCCGTTAGATACGCGGCAAACATCACTGGTTGCTCGA
AATGGATTACCATTCGATATCGGGCAGTGCAGTTGGTGTTTTTTGGGGGGGTTGTTGTTTAGCTACTGCTGT
TGGCTTAGATTCGATGTTTTGCCTCGACGTCATGAGTTCATGACCCCGGCCTGCCATCGTCATCCGTTTTGC
CTTGTAAGACAGAAGTTGATGCTGTTGTGTGACTCGAGAGCTCCAGGGTATGAGTTCTTGTGACCATATCAC
TGGCTTTCATTCCTATGTATCCTTCAACTTCAACTTCAATCATCTGAACACAGTATTCATGACCCTTTGAAC
CCTGAGATGAGCTTAGACTGGAGGCGTCTAAAAACTCGTGATATACGCAGAGTCCTCTCAAGTTTTCTAGGG
GACCCTCTTTCTATCTAGCAGGCTACTAGGTACTTACTAGGAA

>carP F. fujikuroi
1357 bp (1238 overlapping in *F. oxysporum carP* sequence

AGTTGATTCACCCGATGGCCGCTGCAACTGCAGCGTTGTCTCGATCGTCCTCGTCAGCCAGAATCTCGGAAA
CAAGAAACAGCCGAGAGTCCCTTTTACGATAACCCCCGACTCACAACGGACTTTGGGTGCATTTTTTCGACT
GCGCGCGCGCTCTGTATTCCGCTTCCCGCGGACGTTGCAAGAAGGAAAAGAGGAGATTGTCCTATTCTGTAC
TCCGTACACACACAAACGAGAGCAAGATCGGCGACAGATGCGAAACTGGGGTCTTTTGTCGCGGGGACAAAC
AAGTCTTGATCGTGTACCGTCTACAACGCTACAGTCCATCTTGTACCGACAACCCTGCCCTGCCCTGCCCTG
TATCCAATGCTTCTTGTGGTTTCCTGCATCGCGATCGGTGATACTGGTTCCTATGGTAATTGGTCATGCTTG
CGCTTGTGGAGGGAGGGCAACTCTTCGGTTACGCTGTGAAGAAGGGGAAAGTTTCCTTTGTCTGTCTCAATC
CTATCAGCTCAGTCAAGAGTTGAGCTCCCTCCCGTCCCCATCACAAATCTTGGATCTTGGATCTTGGGTCTG
AACGTTTTCCTTGGACCCATTGAGCTGGGATGTGTTTTATTTCAGGTTCATCCTCCCATGCATCCCTGGTTT
ACAGCACAGCGCACCACAACACAACACAACACTGCACTGCACTGCACTGCATAACATAGCACACCAAAAGCA
GGGAATTAATTAACAAGCTCCCGATTGTCGAGATGAGTGAGGGGGTTTAGGTTCCGGTTGCTGCAGCAAAAC
GGCGACCAAAGTCTATCTATCCGGTGCTCGAAATGGATTACCATTCGATATCCGGCAGCGCCGTTGGTGTTT
TTGCCTCGAATACTCCAAGATAGTTACTGCTATTGGCTTAGATTTGAGGTTTTCGCCTCGGCGTCATGAGTC
CATGACCCCGTCATCCGTTTTGCCTTGTAAGACAGAAGTTGATGCTGCTGTTGTGTGACTCGAGAGTTCCAG
GGTACGAGTTCCTATGACCATATCACTGGCTTCATTCCTGTGTGTCTACTCCTTAGGTGTAGGTGCCCTTCC
TTTAATCACCTGAGCACAGTATTCCAAGACCGTTTGAACCCCGAGATGAGTTTAGACTGGAGGAGGCGTCTA
AAACTCGTGATATACGCAGGGACCTCTCAAGTTTTCTAGGGGACCCTCTTTCTGTACAGCAGAGGCCACTAG
GCGCTTGCTAGGAACTGCCTAGTGCTAAAGTTGGAAAAATCTACAACATTTCCCAGTTGTCTAGCCTGTTCC
GAAGTACTGTATACATGCCAGAGACTAGGGCCTCTCCACCAAGTTTCTCGATATATTTTGT

B

```
Fo-carP  CTCTCAACTATTAAGTAGCGTCGATGTGCCAGTTGATTCACCCGATGGCCGTTGCAACTG
Ff-carP  ------------------------------AGTTGATTCACCCGATGGCCGCTGCAACTG
                                       ********************* ********

Fo-carP  CAGCGTTGTCTCGATCGTCCTCGTCAGCCAGAATCTCGAAAGGAAAAAGAGTCGAGAGAG
Ff-carP  CAGCGTTGTCTCGATCGTCCTCGTCAGCCAGAATCTCGGAAA----------CAAGAAAC
         ************************************** **           * *** *

Fo-carP  AAGCGAGAACGACACCCCTTACGATAACCCCCGACTCACAACGGATTTTAGGTGCATTTT
Ff-carP  AGCCGAGAGTC---CCTTTTACGATAACCCCCGACTCACAACGGACTTTGGGTGCATTTT
         *  *****      **  *************************** *** **********

Fo-carP  --------CGAGCGCGCGCTGTATTCCGTTTTCTGCGGACCGTCCAAGAGTGACAAGAGG
Ff-carP  TTCGACTGCGCGCGCGCTCTGTATTCCGCTTCCCGCGGACGTTGCAAGAAGGAAAAGAGG
                 ** ****** ********** ** * ******  * *****  ** ******

Fo-carP  AGGTTGT-------------------ACACACAAACAAGAGCAAGATCGGCGACAGATGC
Ff-carP  AGATTGTCCTATTCTGTACTCCGTACACACACAAACGAGAGCAAGATCGGCGACAGATGC
         ** ****                   ********** ***********************

Fo-carP  GAAACTAGGGTCCTCTGCCGCGGGGAGAAA--GGTCTTGATCGAGTACCGTCTACAATAC
Ff-carP  GAAACTGGGGTCTTTTGTCGCGGGGACAAACAAGTCTTGATCGTGTACCGTCTACAACGC
         ****** ***** * ** ******** ***   ********** *************  *

Fo-carP  TGCA-----TCTT-CACTGAGAACCCTGCATTCTCGT----------------------T
Ff-carP  TACAGTCCATCTTGTACCGACAACCCTGCCCTGCCCTGCCCTGTATCCAATGCTTCTTGT
         * **     ****  ** ** ********  *  * *                      *

Fo-carP  GGTTTCCTGCATCGCGATCTGTGATACTGGTCTCTATGGTTATTGGCCATGCTTGCGCTT
Ff-carP  GGTTTCCTGCATCGCGATCGGTGATACTGGTTCCTATGGTAATTGGTCATGCTTGCGCTT
         ******************* ***********  ******* ***** *************

Fo-carP  CTGGAGGG----CAACTTTTCGGTTACGCTATGAAGAAGGGGAAAGTTTCCTTTGTCTGC
Ff-carP  GTGGAGGGAGGGCAACTCTTCGGTTACGCTGTGAAGAAGGGGAAAGTTTCCTTTGTCTGT
          *******    ***** ************ ****************************

Fo-carP  CTTGTCTCAATCCTCAGCTCAGTCAAGAGG---------------CCCCGGCCCAGATCT
Ff-carP  CTCAATCCTAT---CAGCTCAGTCAAGAGTTGAGCTCCCTCCCGTCCCCATCACAAATCT
         **     * **   ***************                ****  * ** ****

Fo-carP  TGAATCTTGGATCTTGGATCTGAACGTTTTTCTTGGCCCCATTGAGCTGGGATGTGTTTT
Ff-carP  TGGATCTTGGATCTTGGGTCTGAACGTTTTCCTTGGACCCATTGAGCTGGGATGTGTTTT
         ** ************** ************ ***** ***********************

Fo-carP  ATTTTAGGTTCATCCTCCCATGCATCCCCGGTTGGCAGCACAGCGCACCACAAAACAGCA
Ff-carP  ATTTCAGGTTCATCCTCCCATGCATCCCTGGTTTACAGCACAGCGCACCACAACACAACA
         **** *********************** ****  ****************** *** **

Fo-carP  AAAGCAGGGAACTGACGAGCTCCCGATTATCGAG------ATGAGTGAGGGGTTTAGGCT
Ff-carP  CAA-CACTGCACTGCACTGCACTGCATAACATAGCACACCAAAAGCAGGGAATTAATTAA
          ** **  * ****    ** *   ** *   **      *  **   **  ** *

Fo-carP  CCGTCCCTCGGTTGCTGCAACGGGCACCAAAGTCTATCTATCTGGTGCTGCACCTTGGCC
Ff-carP  CAAGCTCCCGATTGTCGAGATGAGTGAGGGGGTTTAGGTTCCGGTTGCTGCAGCAAAACG
         *   * * ** ***  *  * * *       ** **  *  * * ******* *    *
```

```
Fo-carP   GTTAGATACGCGGCAAACATCACTGGTTGCTCGAAATGGATTACCATTCGATATCGGGCA
Ff-carP   GC--GACCAAAGTCTATC--TATCCGGTGCTCGAAATGGATTACCATTCGATATCCGGCA
          *   **     * * * *   *   * **************************** ****

Fo-carP   GTGCAGTTGGTGTTTTT--TGGGGGGGTTGTTGTTTAGCTACTGCTGTTGGCTTAGATTC
Ff-carP   GCGCCGTTGGTGTTTTTGCCTCGAATACTCCAAGATAGTTACTGCTATTGGCTTAGATTT
          * ** ************     *     *      *** ******* ************

Fo-carP   GATGTTTT-GCCTCGACGTCATGAGTTCATGACCCCGGCCTGCCATCGTCATCCGTTTTG
Ff-carP   GAGGTTTTCGCCTCGGCGTCATGAGTCCATGACCCCG-----------TCATCCGTTTTG
          ** ***** ****** ********** **********           ************

Fo-carP   CCTTGTAAGACAGAAGTTGATGCTG---TTGTGTGACTCGAGAGCTCCAGGGTATGAGTT
Ff-carP   CCTTGTAAGACAGAAGTTGATGCTGCTGTTGTGTGACTCGAGAGTTCCAGGGTACGAGTT
          *************************   **************** ********* *****

Fo-carP   CTTGTGACCATATCACTGGCTTTCATTCCTATGTATC----CTTCAACTTCAA-------
Ff-carP   CCTATGACCATATCACTGGCTT-CATTCCTGTGTGTCTACTCCTTAGGTGTAGGTGCCCT
          * * ****************** ******* *** **    * * *  *  *

Fo-carP   --CTTCAATCATCTGAACACAGTATTC-ATGACCCTTTGAACCCTGAGATGAGCTTAGAC
Ff-carP   TCCTTTAATCACCTGAGCACAGTATTCCAAGACCGTTTGAACCCCGAGATGAGTTTAGAC
            *** ***** **** ********** * **** ********* ******** ******

Fo-carP   TGGAGG---CGTCTAAAAACTCGTGATATACGCAGAGTCCTCTCAAGTTTTCTAGGGGAC
Ff-carP   TGGAGGAGGCGTCTAAAA-CTCGTGATATACGCAGGGACCTCTCAAGTTTTCTAGGGGAC
          ******   ********* **************** * **********************

Fo-carP   CCTCTTTCTATCTAGCAG--GCTACTAGGTACTTACTAGGAA------------------
Ff-carP   CCTCTTTCTGTACAGCAGAGGCCACTAGGCGCTTGCTAGGAACTGCCTAGTGCTAAAGTT
          ********* *  *****  ** ******  *** *******

Fo-carP   ------------------------------------------------------------
Ff-carP   GGAAAAATCTACAACATTTCCCAGTTGTCTAGCCTGTTCCGAAGTACTGTATACATGCCA

Fo-carP   ----------------------------------------
Ff-carP   GAGACTAGGGCCTCTCCACCAAGTTTCTCGATATATTTGT
```

Figure S3. (A) Open reading frames in the forward (A) and reverse (B) orientations of *carP* sequence of *F. oxysporum* (oxy) and *F. fujikuroi* (fuj). F and R numbers are as indicated in Fig. 3. ORF lengths are indicated in parentheses. Significant coincidences between overlapping ORFs are indicated below.

Forward ORFs in *F. fujikuroi* and *F. oxysporum carP*

```
>oxyF1 (85)
MCQLIHPMAVATAALSRSSSSARISKGKRVEREARTTPLTITPDSQRILGAFSSARCIPFSADRPRVTRGGCTHKQEQD
RRQMRN

>oxyF2 (42)
MVIGHACASGGQLFGYAMKKGKVSFVCLVSILSSVKRPRPRS

>oxyF3 (27)
MCFILGSSSHASPVGSTAHHKTAKAGN

>oxyF4 (131)
MHPRLAAQRTTKQQKQGTDELPIIEMSEGFRLRPSVAATGTKVYLSGAAPWPLDTRQTSLVARNGLPFDIGQCSWCFLG
GLLFSYCCWLRFDVLPRRHEFMTPACHRHPFCLVRQKLMLLCDSRAPGYEFL

>oxyF5 (37)
MDYHSISGSAVGVFWGGCCLATAVGLDSMFCLDVMSS

>oxyF6 (30)
MSSCDHITGFHSYVSFNFNFNHLNTVFMTL

>oxyF7 (27)
MSLDWRRLKTRDIRRVLSSFLGDPLSI


>fujF1 (29)
MAAATAALSRSSSSARISETRNSRESLLR

>fujF2 (107)
MRNWGLLSRGQTSLDRVPSTTLQSILYRQPCPALPCIQCFLWFPASRSVILVPMVIGHACACGGRATLRLRCEEGESFL
CLSQSYQLSQELSSLPSPSQILDLGSWV

>fujF3 (34)
MLLVVSCIAIGDTGSYGNWSCLRLWREGNSSVTL

>fujF4 (33)
MLALVEGGQLFGYAVKKGKVSFVCLNPISSVKS

>fujF5 (33)
MCFISGSSSHASLVYSTAHHNTTQHCTALHCIT
```

Higher similarities between *F. fujikuroi* and *F. oxysporum* forward ORFs

```
oxyF1  MCQLIHPMAVATAALSRSSSSARISKGKRVEREARTTPLTITPDSQRILGAFSSARCIPFSADRPRVTRGGC
fujF1  -------MAAATAALSRSSSSARISET----RNSRESLLR---------------------------------
              **.***************:     *::* : *
oxyF1  THKQEQDRRQMRN
fujF1  -------------

oxyF2  MVIGHACASGGQLFGYAMKKGKVSFVCLVSILSSVKRPRPRS
fujF4  ML---ALVEGGQLFGYAVKKGKVSFVCLNPI-SSVKS-----
       *:   * ..********:********** .* ****

oxyF3  MCFILGSSSHASPVGSTAHHKTAKAGN------
fujF5  MCFISGSSSHASLVYSTAHHNTTQHCTALHCIT
       **** ******* * *****:*::  .
```

Reverse ORFs in *F. fujikuroi* and *F. oxysporum carP*

```
>oxyR1
MQETNENAGFSVKMQYCRRYSIKTFLPAAEDPSFASVADLALVCVYNLLLSLLDGPQKTEYSARSKMHLKSVVSRGLS

>oxyR2
MVIHFEQPVMFAAYLTAKVQHQIDRLWCPLQQPRDGA

>oxyR3
MTMAGRGHELMTSRQNIESKPTAVAKQQPPQKTPTALPDIEW

>oxyR4
MIEVEVEGYIGMKASDMVTRTHTLELSSHTTASTSVLQGKTDDDGRPGS


>fujR1
MDCSVVDGTRSRLVCPRDKRPQFRICRRSCSRLCVYGVQNRTISSFPSCN
VRGKRNTERARSRKNAPKVRCESGVIVKGTLGCFLFPRFWLTRTIETTLQ
LQRPSGEST

>fujR2
MQETTRSIGYRAGQGRVVGTRWTVAL

>fujR3
MTNYHRNQYHRSRCRKPQEALDTGQGRAGLSVQDGL

>fujR4
MGPRKTFRPKIQDPRFVMGTGGSSTLD

>fujR5
MLCSAVQCSVVLCCGALCCKPGMHGRMNLK

>fujR6
MVIHFEHRIDRLWSPFCCSNRNLNPLTHLDNRELVN

>fujR7
MVIGTRTLELSSHTTAASTSVLQGKTDDGVMDS
```

Higher similarities between *F. fujikuroi* and *F. oxysporum* reverse ORFs

```
oxyR4  MIEVEVEGYIGMKASDMVTRTHTLELSSHTT-ASTSVLQGKTDDDGRPGS
fujR7  ----------------MVIGTRTLELSSHTTAASTSVLQGKT-DDGVMDS
                       **  *:********* ********** ***  .*
```

Figure S4. Test of *Fo-carP* coding capacity through 3-base periodicity according to the Fickett TESTCODE statistic using Tcode of EMBOSS, windows of 200, stepping 3. Segments above the green line are predictable coding sequences. Segments below the red line are rejected as coding sequences. ORF F4 of *Fo-carP* is delimited by two red vertical lines.

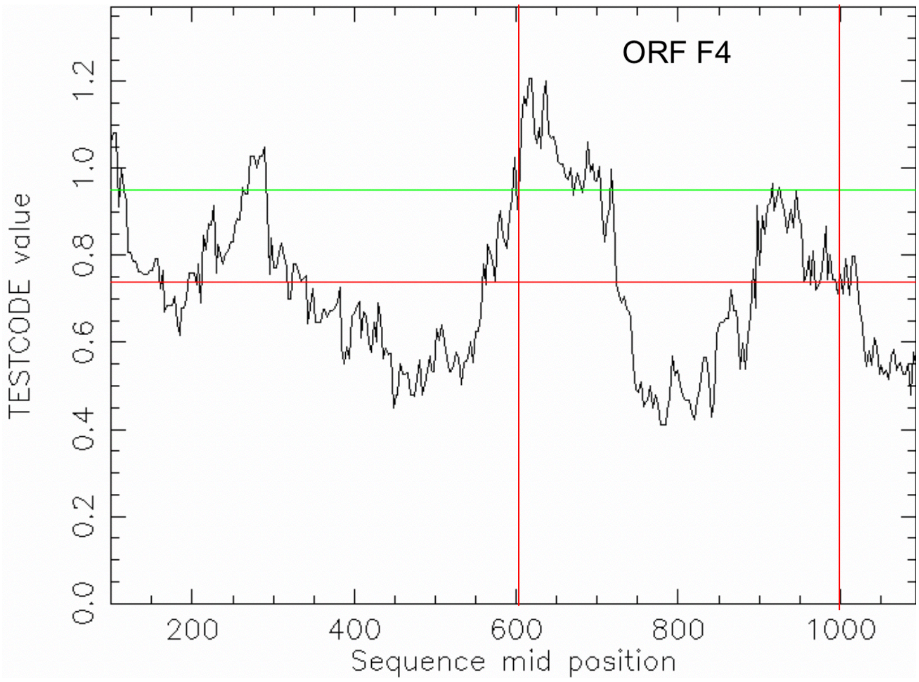

Figure S5. Test of *Fo-carP* coding capacity through codon usage. A codon usage table for *F. oxysporum* was constructed with CUSP (EMBOSS) from the CDS of *F. oxysporum* obtained from FungiDB. The results were graphed with Syco, window 30. Segments above the blue line are in agreement with codon usage preferences in *F. oxysporum*. The data do not support appropriate codon usage in ORF F4, delimited by two red vertical lines.

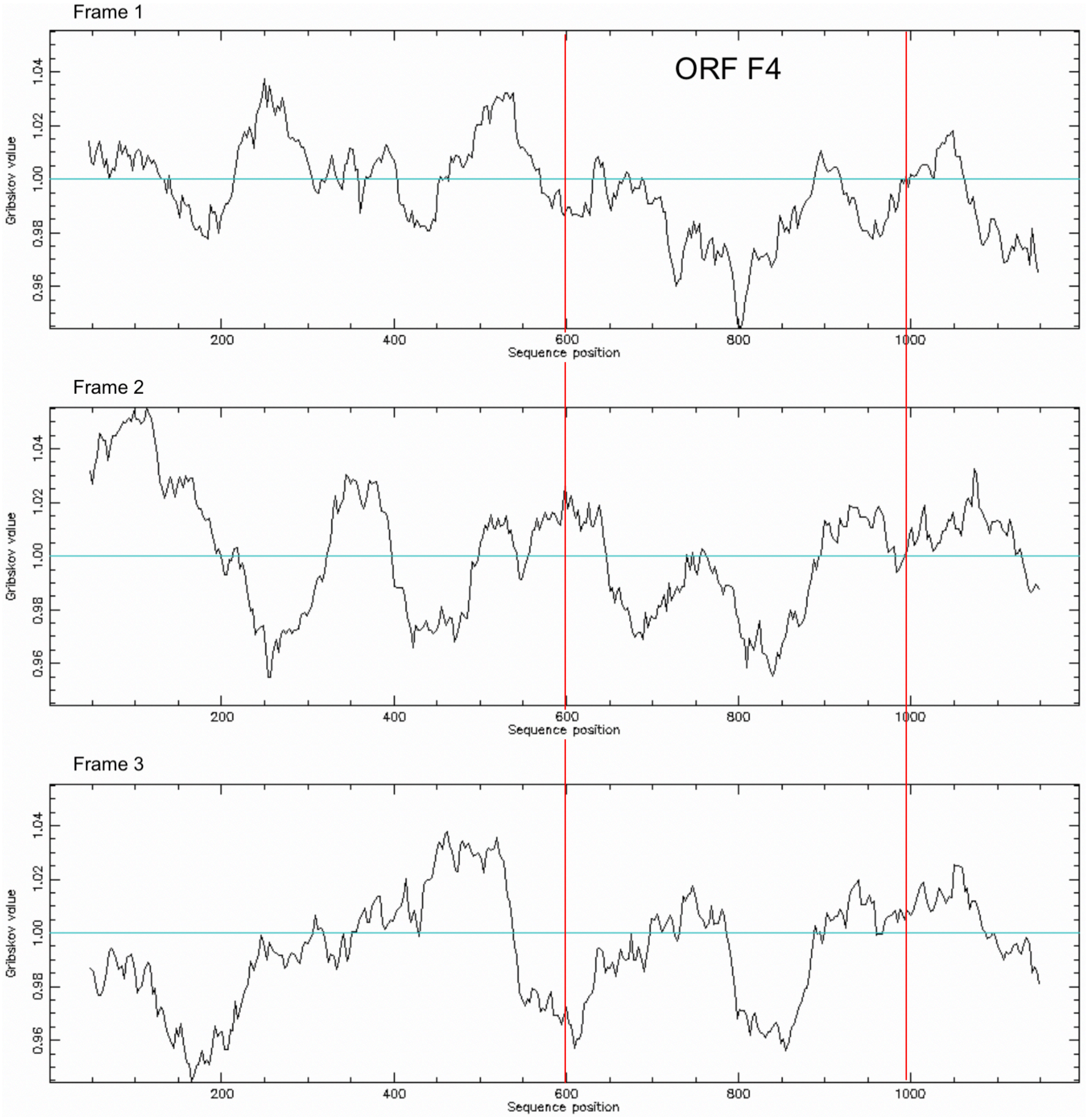

Figure S6. Sequences of *carP* orthologous to *Fo-carP* in *Fusarium* genomes with 100% coverage. Sequences were retrieved after a BLASTn with *Fo-carP* in FungiDB and discontinuous MEGABLAST against WGS at NCBI. A single *carP* sequence was selected for each species except *F. fujikuroi* FUS01, which apparently is a wrong assignation of a *F. oxysporum* strain.

```
>carP_F_oxysporum_00293
CTCTCAACTATTAAGTAGCGTCGATGTGCCAGTTGATTCACCCGATGGCCGTTGCAACTGCAGCGTTGTCTCGATCGTCC
TCGTCAGCCAGAATCTCGAAAGGAAAAAGAGTCGAGAGAGAAGCGAGAACGACACCCCTTACGATAACCCCCGACTCACA
ACGGATTTTAGGTGCATTTTCGAGCGCGCGCTGTATTCCGTTTTCCGCGGACCGTCCAAGAGTGACAAGAGGAGGTTGTA
CACACAAACAAGAGCAAGATCGGCGACAGATGGGAAACTAGGGTCCTCTGCCGCGGGGAGAATGGTCTTGATCGAGTACC
GTTGAGAACCCTGCATTCTTGTGGTTTCCTGCATCGCGATCTGTGATACTGGTCTCTATGGTTATTGGCCATGCTTGCGC
TTCTGGAGGGCAACTTTTCGGTTACGCTATGAAGAAGGGGAAAGTTTCCTTTGTCTGCCTTGTCTCAATCCTCAGCTCAG
TCAAGAGGCCCCAGCCCAAATCTTGAATCTTGGATCTTGGATCTGAACGTTTTTCTTGGCCCCATTGAGCTGGGATGTGT
TTTATTTTAGGTTCATCCTCCCATGCATCCCCGGTTGGCAGCACAGCGCACCACAACACAGCAAAAGCAGGGAACTGACG
AGCTCCCGATTATCGAGATGAGTGAGGGGTTTAGGCTCCGTCCCTCGGTTGCTGCAACGGGCACCAAAGTCTATCTATCT
GGTGCTGCACCTTGGCCGTTAGATACGCGGCAAGCATCACTGGTTGCTCGAAATGGATTACCATTCGATATCGGGCAGTG
CAGTTGGTGTTTTTTTGGGGGGGGTTTGTTGTTTAGCTACTGCTGTTGGCTTAGATTCGATGTTTTGCCTCGACGTCATG
AGTTCATGACCCCGGCCTGCCATCGTCATCCGTTTTGCCTTGTAAGACAGAAGTTGATGCTGTTGTGTGACTCGAGAGCT
CCAGGGTATGAGTTCTTGTGACCATATCACTGGCTTTCATTCCTATGTATCCTTCAACTTCAACTTCAATCATCTGAACA
CAGTATTCATGACCCTTTGAACCCTGAGATGAGCTTAGACTGGAGGCGTCTAAAACTCGTGATATACGCAGGGTCCTCTC
AAGTTTTCTAGGGGACCCTCTTTCTATCTAGCAGGCTACTAGGTACTTACTAGGAA

>carP_F_fujikuroi_FUS01
CTCTCAACTATTAAGTAGCGTCGATGTGCCAGTTGATTCACCCGATGGCCGTTGCAACTGCAGCGTTGTCTCGATCGTCC
TCGTCAGCCAGAATCTCGAAAGGAAAAAGAGTCGAGAGAGAAGCGAGAACGACACCCCTTACGATAACCCCCGACTCACA
ACGGATTTTAGGTGCATTTTCGAGCGCGCGCTGTATTCCGTTTTCCGCGGACCGTCCAAGAGTGACAAGAGGAGGTTGTA
CACACAAACAAGAGCAAGATCGGCGACAGATGCGAAACTAGGGTCCTCTGCCGCGGGGAGAAAGATCTTGATCGAGTACC
GTCTACAATACTGCATCTTCACTGAGAACCCTGCATTCTTGTGGTTTCCTGCATCGCGATCTGTGATACTGGTCTCTATG
GTTATTGGCCATGCTTGCGCTTCTGGAGGGCAACTTTTCGGTTACGCTATGAAGAAGGGGAAAGTTTCCTTTGTCTGCCT
TGTCTCAATCCTCAGCTCAGTCAAGAGGCCCCAGCCCAAATCTTGAATCTTGGATCTTGGATCTGAACGTTTTTCTTGGC
CCCATTGAGCTGGGATGTGTTTTATTTTAGGTTCATCCTCCCATGCATCCCCGGTTGGCAGCACAGCGCACCACAACACA
GCAAAAGCAGGGAACTGACGAGCTCCCGATTATCGAGATGAGTGAGGGGTTTAGGCTCCGTCCCTCGGTTGCCGCAACGG
GCACCAAAGTCTATCTATCTGGTGCTGCACCTTGGCCGTTAGATACGCGGCAAGCATCACTGGTTGCTCGAAATGGATTA
CCATTCGATATCGGGCAGTGCAGTTGGTGTTTTTTTGGGGGGGTTGTTGTTTAGCTACTGCTGTTGGCTTAGATTCGATG
TTTTGCCTTGACGTCATGAGTTCATGACCCCGGCCTGCCATCGTCATCCGTTTTGCCTTGTAAGACAGAAGTTGATGCTG
TTGTGTAACTCGAGAGCTCCAGGGTATGAGTTCTTGTGACCATATCACTGGCTTTCATTCCTATGTATCCTTCAACTTCA
ACTTCAATCATCTGAACACAGTATTCATGACCCTTTGAACCCTGAAATGAGCTTAGACTGGAGGCGTCTAAAAACTCGTG
ATATACGCAGGGTCCTCTCAAGTTTTCTAGGGGACCCTCTTTCTATCTAGCAGGCTACTAGGTACTTACTAGGAA

>carP_F_proliferatum_ET1
CTCTCAACTATTAAGTAGCGTCGATGCGCCAGTTGATTCACCCGATGGCCGCTGCAACTGCAGCGTTGTCTCGATCGTCC
TCGTCAGCCAGAATCTCGGAAACAAGAAACAGCCGAGAGTCCCTTTCACGATAACCCCCGACTCACAACGGACTTTGGGT
GCATTTTTTCGACTGCGCGCGCGCTCTGTATTCCGCTTCCCGCGGACGTTGCAAGAAGGAAAAGAGGAGATTGTCCTATT
CTGTACACACACAAACGAGAGCAAGATCGGCGACAGATGCGAAACTGGGGTCTTTTGCCGCGGGGACAAACAAGTCTTGA
TCGAGTACTGTCTACAACGCTACAGTCCATCTTGTACTGAGAACCCTGCCCTGCATCGCGATCGGTGATACTGGTTCCTA
TGGTAATTGGTCATGCTTGCGCTTCTGGAGGGAGGGCAACTCTTCGGTTACGCTGTGAAGAAGGGGAAAGTTTCCTTTGT
CTGTCTCAATCCTATCAGCTCAGTCAAGAGTTGATTCCCCTCCCGTCCCCATCACAAATCTTGGATCTTGGATCTGAACG
TTTTTCTTGGCTCCATTGAGCTGGGATGTGTTTTATTTTAGGTTCATCCTCCCATGCATCCCTGGTTTACAGCACAGCGC
ACCACAACACAACACAACACTGCACTGCACTGCACTGCATAACACGGCACAACAAAAGCAGGGAATTAATTAACAAGCTC
CCGATTGTCGAGATGAGTGAGGGGGTTTAGGTTCCGGTTGCTGCAGCAAAACGGCGACCAAAGTCTATCTATCCGGTGCT
CGAAATGGATTACCATTCGATATCCGGCAGCGCCGTTGGTGTTTTGCCCCGAATACCTCAAGATAGTTACTGCTAGTGGC
TTAGATTTGAGGTTTTCGCCTCAGCGTCATCAGTCCATGACCCCGTCATCCGTTTTGCCTTGTAAGACAGAAGTTGATGC
TGCTGTTGTGTGACTCGAGAGCTCCAGGGTACGAGTTCCTATGACCATATCACTGGCTTCATTCCTGTGTGTCTACTCCG
TAGGTGTAGGTGTCCTTCAATCAATCACCTGAGCACAGTATTCCATGACCGTTTGAACCCTGAGATGGGTTTAGACTGGA
GGAGGCGTCTAAAACTCGTGATATACATACGCAGGGTCCTCTCAAGTTTTCTAGGGGACCCTCTTTCTCTCCAGCAGAGG
CCACTAGGTGCTTGCTAGGAA
```

Figure S6 (cont.)

```
>carP_F_verticillioides_7600
CTCTCAACTATTAAGTAGCGTCGATGCGCCAGTTGATTCACCCGATGGCCGTTGCAACTGCGGCGTTGTCTCGATCGTCC
TCGTCAACCAGAATCTCTTTTCCAGAATCTCAAGAGAGAGAGAGAGAGAGAGAGAGAGAGAGAGAGAGAGAGAGAGAGAG
AGAAGCGACAACGACAACGACAACGACAACGACACCCCTTTTTGCGATAACCCCCGACTCGCGCGCGCGCTTTGTGTATT
CCATTTTCCGCGGACGAAAAGAGGAGATTGTCCTGTCCTGTACTGTTATTTGTACATACATACACACACAAACAAGAGCA
AAGATCGGCGACAGATGCGAAACTGGGGTCAATCTCTCTCTCCCTCATCTTCTGCCGCGGGGACAAAGAGGTCTTGATCG
AGTACCGTCTACATCTCCATCTACAATGTTACACTCCCATCTTCTACTGAGAACCCTGCCCTGCATCGCGATCTGTGATA
CTGGTCTCTATTTCAATTGGTCATGCTTGCGCTTCTGGAGGGCAACTCTCAACTACTACTTTTCGGTTACCTTACGCTGT
GAAGAAGAGGGTAGCTACTGTAAGTTTTCCTTTGTCTGTCTTGTCGTGTCCCAATTCTCTTAGCTCAATCGAGGTCCCCC
CATTCCAAATCTTGAATCTTGGATCTGAACGATTGTCTTGGTCCCATTGAGCTGGGATGTTTTATTTTAACTAGGTTCAT
CCCCCCGGGAATGCATCCCCAGTTTCGCAGCACAGGCTACAGCACAGCACCAACGCACCACAAGACAGCACAGCAAAAGC
AGGCAATTAACGCTCCCGATTATCGAACTGAATGAGGGGGTTTATTAGGGGGTTTAGGTTCCGGTTGCTGCTGCACAATG
GCGACCAAAGTCTATCTATCCTGTGCTTCAAATAGATTACCATTCGAAATACGACAGCGCTTGGTTTCTTTACTCCGAAA
TGTTCCACGGTAGTTACTGCTATTGGCTTGGATTTGGTGTTTCGGCCTCCACGTCATTGAGTCCATGACCCCCGGCCTGC
CATCGTCATCCGTTTTTGCCCTGTAAGACACAGGTTGATACATACTGTTGTGTGACTCTAGGGCTTCAGGGCACGAGTTC
TTGTGACCACTTCACTGGCTTCGTTCCTCTGTCTGGGTGTCCTTTAGCCTCAGCTTCAATCACCCGAGCACAGTATTTCC
ATGACCGTTTGAACCCTGAGATGAGTTTAGACTGGAGGTGTCTAAAAACTCGTGATATACGCAGGGTCCTCTCAAGTCTT
CTAGGGGACCCTCTTCTGTCCAAGGTAGTTAGCAGGTTACTAGGTGCTTACTAGAAC

>carP_F_fujikuroi_IMI_58289
CTCTCGACTATTAAGTAGCGTCGATGCGCCAGTTGATTCACCCGATGGCCGCTGCAACTGCAGCGTTGTCTCGATCGTCC
TCGTCAGCCAGAATCTCGGAAACAAGAAACAGCCGAGAGTCCCTTTTACGATAACCCCCGACTCACAACGGACTTTGGGT
GCATTTTTTCGACTGCGCGCGCGCTCTGTATTCCGCTTCCCGCGGACGTTGCAAGAAGGAAAAGAGGAGATTGTCCTATT
CTGTACTCCGTACACACACAAACGAGAGCAAGATCGGCGACAGATGCGAAACTGGGGTCTTTTGTCGCGGGGACAAACAA
GTCTTGATCGTGTACCGTCTACAACGCTACAGTCCATCTTGTACCGACAACCCTGCCCTGCCCTGCCCTGTATCCAATGC
TTCTTGTGGTTTCCTGCATCGCGATCGGTGATACTGGTTCCTATGGTAATTGGTCATGCTTGCGCTTGTGGAGGGAGGGC
AACTCTTCGGTTACGCTGTGAAGAAGGGGAAAGTTTCCTTTGTCTGTCTCAATCCTATCAGCTCAGTCAAGAGTTGAGCT
CCCTCCCGTCCCCATCACAAATCTTGGATCTTGGATCTTGGGTCTGAACGTTTTCCTTGGACCCATTGAGCTGGGATGTG
TTTTATTTCAGGTTCATCCTCCCATGCATCCCTGGTTTACAGCACAGCGCACCACAACACAACACAACACTGCACTGCAC
TGCACTGCATAACATAGCACACCAAAAGCAGGGAATTAATTAACAAGCTCCCGATTGTCGAGATGAGTGAGGGGGTTTAG
GTTCCGGTTGCTGCAGCAAAACGGCGACCAAAGTCTATCTATCCGGTGCTCGAAATGGATTACCATTCGATATCCGGCAG
CGCCGTTGGTGTTTTTGCCTCGAATACTCCAAGATAGTTACTGCTATTGGCTTAGATTTGAGGTTTTCGCCTCGGCGTCA
TGAGTCCATGACCCCGTCATCCGTTTTGCCTTGTAAGACAGAAGTTGATGCTGCTGTTGTGTGACTCGAGAGTTCCAGGG
TACGAGTTCCTATGACCATATCACTGGCTTCATTCCTGTGTGTCTACTCCTTAGGTGTAGGTGCCCTTCCTTTAATCACC
TGAGCACAGTATTCCAAGACCGTTTGAACCCCGAGATGAGTTTAGACTGGAGGAGGCGTCTAAAACTCGTGATATACGCA
GGGACCTCTCAAGTTTTCTAGGGGACCCTCTTTCTGTACAGCAGAGGCCACTAGGCGCTTGCTAGGAA

>carP_F_udum_F_02845
CTCTCAACTATTAAGTAGCGTCGATGCGCCAGTTGATTCACCCGATGGCCGTTGCAACTGCGGCGTTGTCTCGATCGTCC
TCGTCAACCAGAATCTCCAAAGGAATCGAGAGAGAGACGACGACAACGACACCCCTTTTTACGATAACCCCCGACTCGCA
ACGGATTTTATTAGAGCGCGCGCTGTATTCCGTCTTCCGCGGACGTTCCAAGAGGGAAAAAGAGGAGATTGTCCTGTACA
ACACACAGACAAACAAGAGCAAAGATCGGCGACAGATGCGAAACTGGGGTCCCTCTCTCTGCCGCGGGGACACGACGAGG
TCTTGATCGAGTACCATCTACAATGCTACAGAACATCTTCTACTGAGAACCCTGCCCTGCATCGCGATCTGTGATACTGG
TCTCTATGGTAATTGGTCATGCTTGCGCTTCTGGAGGGCAACTTTTCAACTTTTCGGTTACGCTGAGAAGAAGAGATTAA
GTTTCCTTTGTCTGTCTTGTCTCAATTCTCTCAGTCAGTCAAGAGCCCCTCCCCATCCCCATCCCCATCCCCAAATCTTG
GATCTTGGATCTTGGATCTGAACGGTTTTCTTGGCCCCATTGAGCTGGGATTTCTTATTTTAGGTTCATCTTCCCATGCA
TCCCCGGTTTGCAGCACAGCACAGGCTACAGCGCAGCACACCTCAACACTGCATAGTATATCACAGCAAAAGCAGGGAAT
CAACATGCTCCCGATTATCAATCGAAATGAATGAGGGGTTTTAGGTTCCGGTTGCTGCAGCACAACGGCGACCAAAGTCT
ATCTATCTGGTGCTATTGAGTACGCGGTTTAATCACTGGTTGCTCCAAATGGATTACCATTCGATACCAGGCAGCGAATT
TGGTGATTTTNNNNNNNNNNGAATGTCTCAAGGTAGTTACTGCTACTGGCTTAGATTTGATCTTTTGGCCTCGACGTCAT
GAGTCAATGACCCCGGCCTGCCATCGTCATCCGTTTTGCCTTGTAAGACACAAGTTGATGCTGTTGTGTGACTCGAGAGC
TCCAGGGTACGAGTTCTCGTGACCATTTCACTGGCTTTCATTCCTGTGTGTCTAGTCTAGAACCTCAGCTTCAATAACCC
GAGCACAGTATTTCCATGACCGTTTGAACCCTGAGATGAGTTTAGACTGGAGGCGTCTAAAACTCTTGTGATATACATAC
GCAGGGTCCTCTCAAGTTTTCTAGGGGACCCTCTTTCTGTCTAGCAGAGGCTGCTAGGTGCTTACTAGGAA
```

Figure S6 (cont.)

>carP_F_xylarioides_K1
CTCTCAACTATTAAGTAGCGTCGATGCGCCAGTTGATTCACCCGATGGCCGTTGCAACTGCGGCGTTGTCTCGATCGTCC
TCGTCAGCCAGAATCTCCAAAAGAGTCAAGAGAGAGAAGACGACAACGACAACGACACCCCTTTTACGATAACCCCCGAC
TCGCAACGGATTTTATTAGAGCGCGCGCTGTATTCCGTCTTCCGCGGACGTTCCAAGAGGGAAAAAGAGGAGATTGTCCT
GTCCTGTACAACACACAGACAAACAAGAGCAAGATCGGCGACAGATGCGAAACTGGGGTCCCTCTCTTTGCCGCGGGGAC
ACGACGAGGTCTTGATCGAGTACCGTCTACAATGCTACAGTACATCTTCTACTGAGAACCCTGCCCTGCATCGCGATCTG
CGATACTGGTCTCTATGGTAATTGGTCATGCTTGCGCTTCTGGAGGGCAACTTTTCAACTTTTTGGTTACGCTGAGAAGA
AGAGAGTAAGTTTCCTTTGTCTGTCTTGTCTTGTCTTGTCTCAATTCTCTCAGTCAGTCAAGAGCCCCTCCCCATCCCAA
ATCTTGGATCTTGGATCTTGGATCTGAACGGTTTTCTTGGCCCGATTGAGCTGGGATGTTTTATTTTAGGTTCATCTTCC
CATGCATCCCCGGTTTGCAGCACAGCACAGGCTACAGCGCAGCACACCTCAACACTGCATAGCATAGCACAGCAAAAGCA
GGGAATCAACATGCTCCCGATTATCAATCGAAATGAATGAGGGGTTTAGGTTCCGGTTGCTGCAGCACAACGGCGACCA
AAGTCTATCTATCTGGTGCTGTTGAATTCGCGATTTAATCACTGGTTGCTCCAAATGGATTACCATTCGATACCAGGCAG
CGAGTTTGGTGTTTTTGCCCCGAATGTTCCAAGGTAGTTACTGCTATTGGCTTAGATTTGATCTTTTGGCCTCGACGTCA
TGAGTCCATGACCCTGGCCTGCCATCGTCATCCGTTTTGCCTTGTAAGACACAAGTCGATGCTGTTGTGTGACTCGAGAG
CTCCAGGGTACGAGTTCTTGTGACCATTTCACTGGCTTTCATTCCTGTTTGTCTAGTCTAGAACCTCAGCTTCAATCACT
TGAGTACAGTATTTCCATGACCGTTTGAACCCTGAGATGAGTTTAGACTGGAGGCGTCTAAAACTCTCGTGATATACGCA
GGGTCCTCTCAAGTTTTCTAGGGGACCCTCTTTCTGTCTAGCAGAGGCTGCTAGCTA

>carP_F_nygamai_CS10214
CTCTCAACTATTAAGTAGCGTCGATGCGCCAGTTGATTCACGCGATGGCCGTTGCAACTGCGGCGTTGTCTCGATCGTCC
TCGTCAACCAGAATCTCCAGAATCTCGAAAAGAGTCAAGAGAGAGAAGCGACAACGACAACGACAACGACACCCCTTTTA
CGATAACCCCCGACTCACGCACGCGCTGTATTCCGTTTTCCGCGGATGAAAAGAGGGAAAAAGAGGAGATTGTCCCGTAC
TGTACTCACATAAACAAGAGCAAAGATCGGCGACAGATGCGAAACTGGGGTCCCTCTCTCTCACTCTCTCTGCCGCGGGG
ACAAGGAGGTCTTGATCGAGTACCGTCTACATCTACATACAATGCTACAGTACCTAGATCCATCTTCTACCGAGAACCCT
GCCCTGCATCGCGATCTGTGATACTGGTCTCTATGGTAATTGGTCATGCTTGCGCTTCTGGAGGGCAACTTTCAAACTTT
TCGGTTACGCTGTGAAGAGGGTAAGTTTTCCTTTGTCTGCCTTGTCTTGTCTCAATTCTCTCAGCTCAATCGAGGTCCCC
CCATTTCAAATCTTGGATCTTGGATCTCGGATCTGAACGGTTTTCTTGGCCCCATTGAGCTGGGATGTTTTATTTTAGGT
TCATCCTCCCATGCATCCCCGGTTTGCAGCACAGGCTACAGCACAGCACAGCGCACCACAAGACAGCACTGGATGGCACA
GCACAGCAAAAGCAGGGAATTAACATGCTCCCGATTATCGAACTGAATGAGGGGGCTTTAATGGGTTTAGGTTCCGGTTG
CTGCTGCACAACGGCGACCAAAGTCTATCTATTCGGTGCTGTTCATCACTGGTTGCTCAAAATGGATTACCATTCGAAAT
TCGACAGCAAATTTGGTGTTTCTGCCCCGAATGTTCCACGGTAGTTACTGCTATTGGCTTAGATTTGGTGTTTCGGCCTC
CACGTCATGAGTCCATGACCCCCGGCCTGCCATCGTCATCCGTTTTTGCCTTGTAAGACAGAAGTCGATGCTGTTGTGTG
ACTCGAGAGCTCCAGGGTACGAGTTCTTGTGACTACTTCACTGGCTTCATTCCTGTCTGTCTGGGTCTCCTTTAACTTCA
GCTTCGATCACCTGAGCACGGTATTTCCATGACCGTTTGAACCCTGAGATGAGTTTATACTGGAGGCGTCCTAAAACTCG
TGATATACGCAGGGTCCTCTCAAGTTTTCTAGGGGACCCTCTTCTGTCTAAGGTAGTTAGCAGGTTACTAGGTGCTTACT
AGGAA

>carP_F_mangiferae_MRC7560
CTCTCAACTATTAAGTAGCGTCGATGCGCCAGTTGATTCACCCGATGGCCGCTGCAACTGCAGCGTTGTCTCGATCGTCC
TCGTCAGCCAGAATCTCGGAAACAAGAAACAGCCGAAAGACCCTTTTACGATAACCCCCCGACTCACAACGGACTTTGGG
TGCATCCTTTCAACTGCGCGCGCGCGCTGTTACCATTCCGTTTTCCGCGGACGTTGCAAGAAGGAAAAGAGGAGATTGTC
CTAGTTATCCTGTACACACACACACAAACGAGCGCAAGATCGGCGACAGATGCGAAACTGGGGTCTTCTGCCGCGGGGAC
AAACAAGTCTTGATCGAGTACCGTCTACAACGCTACGGTCCATCTTGTACTGAGAACCCTGCCCTGCCCTGCCCTGTATT
CAATGCTTCTTGTGGTTTCCTGCATCGCGATCTGTGATACTGGTCTCTATGATAATTGGTCATGCTTGCGCTTCTGGAGG
GAGGGCAACTTTTCGGTTACGCTGTGAAGAAGGGCAAAGTTCCCTCAGCTCAGTCAAGAGTTGAGCCCCCTCCCGTCCCC
ATCGCAAATCTTGGATCTTGGATCTTGGATCTGAACGGTTTCCTTGGCCCCATTGAGCTGGGATGTGTTTTATTTTAGGT
TCATCCTCGCATGCATCCCTGGATTCCCTGGTTTACAGCCCAGCGCACCACAACACAACACAACACTGCACTGCACTGCA
TAACATAGCACAACAAAAGCAGGGAATTAATTAACAAGCTCCCGATTGTCGAGATGAGTGAGGGGGTTTAGGTTCCGGTT
GCCGCAGCAAAACGGCGACCAAAGTCTATCTGTCCGGTGCTCGAAATGGATTACCATTCGATGTTGGGCAGTGCAGTTGG
TGTTTTTGCCCCGAATACTCCAAGATAGTTACTGCTATTGGCTTAGGTTTGAGGTTTTCGCCTCGGCGTCATGAGTCCCA
GTCCATGACCGACCCCGGCCTGCCATCGTCATCCGTTTTGCCTTGTAAGACAGAAGTTGATGCTGCTGTTGTGTGACTCG
AGAGCTCCAGGGTACGAGTTCCTATGACCATATCACTGGCTTCATTCCTGTGTGTCTACTCCGTAGGTGTAGGTGTCCTT
CAATCAATCACCTGAGCACAGTATTCCATGACCGTTTGAACCCTGAGATGAGTTTAGACTGGAGGAGGCGTCTAAAACTC
GTGATACACGCAGGGTCCTCTCAAGTTTTCTAGGGGACCCTCTTTCTGTCCAGCAGAGGCCACTAGGTGCTTGCTAGGAA

Figure S6 (cont.)

>carP_F_fracticaudum_CBS 137234
CTCTCAACTATTAAGTAGCGTCGATTCGCCAGTTGATTCCCCCGATGGCCGTTGCAACTGCGGCGTTGTCTCGATCGTCC
TCGTCACCCAGAATCTCCAAAACATCAAGAGAGGGAGAAGCGACAACGGCACCCCTTTAACGAAAACCCCCGACTCACAA
CGGATTTTAGTTGTATTTCTTTTCGAGAGAGAGAGGGCGCGCGCGCTGTATTCCGTTGTCCGCGGACGTTCCAGGAGCGA
AAAAGAGGAGATTGTCCTGTACTGTACACACACAAACAAGAGCAAGATCGGCGACAGATGCGAAACTGGGGTCCTTCTCT
CTCTCTGCCGCGGGGAGAAAGAGGTCTTGATCGAGTACCGTCTACATCCAATGCTACAGCACACACACATACACATACAC
ATCTTCTGCTGAGAACCCTGCCCTGGCCATGTACGTTTCCTGTGGTTTCCTGCATTTTCCTGCATCGCGATCTGCGATAA
CTACTGGTCTCTATGGTAATTGGCCATGCTTGCGTTTCTGGGGCGAGTAACTTTTCGGTTATGCTGTGAAGAAGAGGAAA
GTTTCCTTTGTCTGTGTTTCAACTTTTATTATCTTGACGCTCAACTCAGACAAGAACCCCTCCCCTCCCCTCCCTGCCCT
TCCCATCACAAATCTTGAATCTTGGATCTTGGATCTGAACGGTTTTCTTGGCCCAATCGAGCCGGGATGTGTCTTATTTT
AGGTTCATCCTCCCATGCATCCCCGGTTTGCAGCACAGCACAATACAACACAGCATGGCACAGCAATCTGCAAAAGCAAG
GAATGGAATTAGCATGCTCCCGATTATCGAACTGAATCATTGAATGAGAGAGGGCTTGTCGTAGGTTCCGGTTGCTATAT
TGGCGACCAAAGTCTATCTATCTGGTACTGTCGAATTCGCAACTCAATCACTGGTTGCTCGTAATGCATTACCATTTGAA
ATCCGACAGCGTACTTGGTGTTTTTGCCCCCAAGTTATTGAAGTAGTTACTGCTATTGGCTTCGACGTCATGAGTCCATG
ACCCCTGGCCTGCCATCGTCATCCGTTTTGCCTTGTAAGACACAAGTTGATGATGCTGTTGTGTGACTCGCGAGCTCCAG
GGTACGAGTTCTTGTGACCATTTGACTCGTTGGCTTCATTCCTGCGTGTCCGTGTCCGTCAACTTCAGCTTCAATAACCC
GAGCATAGTCTTCCATGACCGTTTGAACCCTGAGATGAGTTTAGACTGAAGGCGTCTAAAACTCTCGTGATATACGCAGG
GTCCTCTCAAGTTTTCTAGGGGACCCTCATTCTGTCTAGCAGCTTTACTAGGTGCTTACTAGGAA

>carP_F_pininemorale_CMW 25243
CTCTCAACTATTAAGTAGCGTCGATTCGCCAGTTGATTCCCCCGATGGCCGTTGCAACTGCGGCGTTGTCTCGATCGTTC
TCGTCAGCCAGAATCTCGAAAACATCAAGAGAGAGAGAGAAGCGACAACAACAACGACACCCCTTTTTACGATAACCCCC
GACTCACAACGGATTTTAGTCGCATTTCTTTTCGAGAGAGAGAGAGAGAGAGCGCACGCGCTGTATTCCGTTGTCCGCGG
ACGTTTCCAAGAGGGCCAAAGAGGAGATTGTCCTGTGCTGTACACACACAAATAAGAGCAAGATCGGCGACAGATGCGAA
ACTGGGGTCCCTTCTCTCTGCCGCGGGGAGAGAGAGGTCTTGATCGAGTACCGTCTACATCCAATGCTACAACACATACA
CATACACATACACATACACATCTTCTACTGAGAACCCTGCCCTGGCCCTGGCCCTGTGGTTTCCTGCATCGCGATCTGTG
ATAGCTACTGGTCTCTATGGTAATTGGCCATGCTTGCGTTTCTGGGGCGAGTAACTTTTCGGTTTCGCTGTGAAGAAGAG
GAAAGTTTCCTTTGTCTGTATTTCAACTTTTATCATCTTAACCCTCAACTCAGACAAGAGCCCCTCCCCTCCCTCCCTCC
CTTCCCATCCCGAATCTTGAATCTTGAATCTTGGATCTTGGATCTGAACGGTTTTCTTGGCCCAATCGAGCTGGGATGTA
TCTTATTTTAGGTTCATCCTCCCATGCATCCCCGGTTTTCAGCACAGCACAATACAACACAACACAGCATGGCATGGCAT
GGCACAGCAATCTGCAAAAGCAAGGAATGGAATTAGCATGCTCCCGATTATCGAACTGAATCATTGAATGAGAGAGGGCT
TGTCGTAGGTTCCGGTTGCTACATTGGCGACCAAAGTCTATCTATCTGGTACTGTCGAATTCGCAACTCAATCACTGGTT
GCTCGAAATGGATTACCATTTGAAATCCGACAGCGTAGTTGATGTTTTTGCCCCCAAGTTTTTGAAGTATGTAGTTACAG
CTATTGGCCTCGACGTCATTGAGTCCTTGACCCCGGCCTGCCATCGTCATCCGTTTTGCCTTGTAAGACACAAGTTGATG
ATGCTGTTGTGTGACTCGCGAGCTCCAGGGTACGAGTTCTTGTGACCATTTGACTCGTTGGCTTCATTCCTGTCCGTGTC
CGTGTCCGTCAACTTCAGCTTCAACCGCCTGAGCATAGTCTTCCATGACCGTTTGAACCCTGAGATGAGTTTAGACTGAA
GGCGTCTAAAACTCTCGTGATATACGCAGGGTCCTCTCAAGTTTTCTAGGGGACCCTCATTCTGTCTAGCAGCTTTACTA
GGTGCTTACTAGGAA

>carP_F_agapanthi_NRRL_31653
CTCTCAACTATTAAGTAGCGTCGATTCGCCAGTTGTTTCCCCCGATGGCCGTTGCAACTGCGGCGTTGTCTCGATCGTCC
TCGTCACCCAGAATCTCGAAAAGATCAAGATCAAGATCAAGAAAGAGAGAGAGAGAGAGAGAGAAGCGACAGCGACAACG
ACACCCCTTTTTACGATAACCCCCGACTCACAACGGATTTTAGTTGCATTTCTTTTCGAGAGAGAGCGCGCGCGCGCTGT
ATTCCGTTGTCCGCGGACGTTCCAAGAGGGAAAAAGAGGAGATTGCCCTGTACTGTACACACACAAACAAACAAACAAGA
GCAAGATCGGCGACAGATGCGAAACTGGGGTCCCTCTCTCTGCCGCGGGGAGAAAGAGGTCTTGATCGAGTACCGTCTAC
AATGCTACAGTACATACACATACACATCTTCTACTGAGAACCCTGTCCTGGCCCTGTACGCTTCGCTTCTTTGTGGTTTC
CTGCATCGCGATCTGTGATAACTACCGGTCTCTATGGTAATTGGCCATGCTTGCGTTTCTGGGGCGGGTAACTTTTCGGT
TAATGTGAAGAAGAGGAAAGTTTCCTTTGTCCTTATTATTTCGACTTTTATCTCAACCCTCAGCTCAGACAAGAGCCCCT
CCCCTTCCTCCCTTCCAATCCCGAATCTTGAATCTTGGATCTTGGATCTGAACGGTTTTCTTGGCCCCATCGAGCCGGGA
TGTGTTTTATTTTAGGTTCATCCTCCCATGCATCCCCGGTTTGCAACACAACACAGCACAACACAGCGCACCACAATACA
ACACAGCATGGCACAGCAAGCTGCAAAAGCAAGGAAATAGAATGCTCCCGATTATCGAACTGAGGGCTTGTATTAGGTTC
CGGCTACTACAGAATATTGGCGACAAAAGTCTATCTATCTGGTGCTGTTGAATTCGCAGCTCAATCACTGGTTGCTCGAA
ATGCATTACCATTCGAAATCCGACAGCGTAGTTGGTGTTTTTGCCCCCGAGTTTTTGAAGTACTGGTAGTTACTGCTATT
GGCCTCGACGTCATGAGTCCGTGACCCCGGCCTACCATCATCATCATCATCATCCATTTTACCTTGTAAGACACAAGTTG
ATGATACTGTTGTATGACTCGAGAGTTCCAGGGTACGAGTTCTTGTGACCATTTCACCCGCTGGCTTCATTCCTGTGTGT
CTAGGTGTCCGTGTCCATCAACCTCAGCTTCAATCACCTGAGCACAGTATTCCATGACCGTTTGAACCCTGAGATGAGTT
TAGACTGAGAAGGCGTCTAAAACTCTCGTGATATACGCAGGGTCCTCTCAAGTTTTCTAGGGGACCCTCTTTCTGCCTAG
CAGCTCTACTAGGTGCTTACCAGGAA

Figure S6 (cont.)

```
>carP_F_sp_Na10
CTCTCAACTATTAAGTAGCGTCGATTCGCCAGTTGATTCCCCCGATGGCCGTTGCAACTGCGGCGTTGTCTCGATCGTCC
TCGTCAGCCAGAATCTCGAAAACCTCAAGAGAGAGAGAAGCGACAACGACAACGACACCCCTTTTTACAATAACCCCCGA
CTCACAACGGACTTTAGTTGCATTTCTTTTCGAGAGAGAGAGAGAGAGAGCGCACGCGCTGTATTCCGTTGTCCGCGGAC
GTTTCCAAGAGGGCCAAAGAGGAGATTGTCCTGTACTGTACACACACAAATAAGAGCAAGATCGGCGACAGATGCGAAAC
TGGGGTCCCTTCTCTCTGCCGCGGGGAGAGAGAGGTCTTGATCGAGTACCGTCTACATCCAATGCTACAGCACATACACA
TACACATACACATCTTCTACTGAGAACCCTGCCCTGGCCCTGGCCCTGTACGTTTCTTGTGGTTTTCCTGCATCGCGATC
TGTGATAGCTACTGGTCTCTATGGTAATTGGCCATGCTTGCGTTTCTGGGGCGAGTAACTTTTCGGTGACGCTGTGAAGA
AGAGTAAAGTTTCCTTTGTCTGTATTTCAACTTTTATTATCTTAACCCTCAACTCAGACAAGAGCCTTTCCCCTCCCCTC
CCCTCCCCTTCCCTTCTCCATCCCAAATCTTGAATCTTGGATCTTGGATCTGAACGGTTTTCTTGGCCCCATCGAGCCGG
GATGTGTCTTATTTTAGGTTCATCCTCCCATGCATCCCCGGTTTGCAGCACAGCACAGCACAGCATGGCACAGCAATCTG
CAAAAGCAAGGAATGGAATTAGCATGCCCCGACTATCGAACTGAATCATTGAATGAGAGAGGGCTTGTCGTAGGTTCTGG
TTGCCACATTGGCGACCAAAGTCTATCTATCTGGTACTGTCGAATTCGCAACTCAACCACTGGTTGCTCGAAATGGATTA
CCATTTGAAATCAGACAGCGTAGTTGATGTTTTTGCCCCCAAGTTTTTGAAGTAGTTACAGCTATTGGCCTCGACGTCAT
GAGTCCTTGACCCCGGCCTGCCATCGTCATCCGTTTTGCTTTGTAAGACACAAGTTGATGATGCTGTTGTGTGACTCGCG
AGCTCCAGGTTACGAGTTCTTGTGACCATTTGACTCGTTGGCTTCATTCCTGTGTCTCCGTGTCCGTGTCCGTGTCCGTC
AACTTCAGCTTCATCAATCACCCGAGCATAGTCTTCCATGACCGTTTGAACCCTGAGATGAGTTTAGACTGAAGGCGTCT
AAAACTCTCGTGATATACGCAGGGTCCTCTCAAGTTTTCTAGGGGACCCTCATTCTGTCTAGCAGCTTTACTAGGTGCTT
ACCAGGAA


>carP_F_algeriense_NRRL_66648
CTCTCAACTATTAAGTAGCGTCGATGCGCCAGTTGATTCTCCCGATGGTCGTTGCATCTGCAGCGTTGTCTCGATCGTCC
TTGTCAGCCAGAATCTCAAGAGGAAACGAGAAAACGAGAACAACCCTACGAACTCCGATTCGTAAGACAAGTTCGAGCGC
GCGCTGTATTCCGTGTTCCGCGGGACGTCGAAGAGAGGGAAGGGGAAAAAACGAGGAGAATGGACACACACAAGCAAGAT
CGGCGAGAGATGCGAAACTAGGGTCCCTGCCGTGGGGAGAAAGGTCTTGATCGAGTATCGTCTACATCTTTGACTGAGAA
CCCTATGTCTTTGTTGTTTTCTGCATCGTGATCTGTGATGCTGGTCTATGGTTATTGGTTACTTCTGGAGGGTAGCTTTT
CGGTTACGCTGTAAAAGAAGAGAAAGGTTTCCCATGTCTCCATTCCCAAAGCCCAAGCCCAAGTCAAATCTTGAATCTGG
AACGTTTTTCTTGGCTCCATCGAGCTGGGATGTGTTTTATTTTAGGTTCATCCTCCATGCGTCCCGGTTGAGTTGGCAGC
ACAGCAAAGCAAAATTCCCTGCTTTTGCTGTCCCGATTATCGAGATGAGTGAGGGGTTTTGGTTTTGGTTCCCTCGGTTG
CTGTAACGGGCAGCCAAAGTCTATCTATCTATCGCTTGCTTGTTGGCTGCAGCTTGGCCGTTGAGATACGGCGCACAGTG
AGTATTGTTACTAGTCGGCCAGCCAGGCAGTGCTAGAAATGTATTACCATTCATTACCAGTGTAATTGAGGATTTTGGGG
TTGTTGGCCTGACGTCATGAGTCTATGACTCCCGGCCTGCTAGCGTCATCCGTTTTTCCCTTGTGAGACAAAAGTTGATG
CTGTCGTGTAACTCGAGAGCTTTTTGGGGTCTAAGTTTTTGTTATTACTGACTCTATTCGCAAGCATTATTTGACATGAA
CGAACCATCACGCCCTTGACACACAGTATTCTTGACACTTTGAACCCTGAGCCTAAACTGGAGGCGTCTAAAACTTGTGA
TATACGCAGGGTCCTTTCACGTTTTCTATGGACCCTCTTTGTGTCTAGTTGCTAACTGTTACTTGCTGGGAA


>carP_F_burgessii_NRRL_66654
CTCTCAACTATTAAGTAGCGTCGATGCGCTAGTTGATTCTCCCGATGGCCGTTGCATCTGCAGCGTTGTCTCGATCGTCC
TTGTCAGCCAGAATCTCAAGAGGAAACGAGAAAACGAGAACAACCCTACGAACCCAGATTCATAAGACATGTTCGAGCAC
GCGCTGTATTCCGTGTTCCGCGGGACGTTGAAGAAAGGGAAGGGGAAAAAAAGAGGAGAATGGACACACACAAGCAAGAT
CGGCGAGAGATGCGAAACTAGGGTCCCTGCCGTGGGGAGAAAGGTCTTGATCGAGTACCGTCTACATCATTGACTGAGAA
CCCTGTGTCTTTGTGGTTTTCTGCATCGTGATCTGTGATGCTGGTCTATGGTTATTGGTTACTTTTGGAGGGTAACTTTT
CGGTTACGCTGTAAAAGAAGAGAAAGGTTTCCCATGTCTCCATTCCCAAAGCCCAAGCCCAAGTCAAATCTTGGATCTGG
AACGTTTTTCTTGGCTCCATCGAGCAGGGATATGTTTTATTTTAGGTTCATCCTCCATGCATCCTGGTTGAGTTGGCAGC
ACAGCAAGCAAAAATCACTGCTTTTGCTGTCCCGATTATCGAGATGAGTGAGGGGTTTTGGCTTTGGTTCCCTCGGTTGC
TGTAACGGGCAGCCAAAGTCTATCTATCTATCTATCTATCGCTTGCTTGCTGGCTGCAGCTTGGCCGTTGAGATACGGCG
CACTGTGAGTATTGTTACTAGTCAGCCAGCCAGGCAGTGCTAGAAATGTATTACCATTCATTACCAGTACAATTGAGGAT
TTTGGGGGTTGTTGGCCCGACGTCATGAGTCTATGACCCCCGGCCTGCCAGCGTCATCCGTTTTCCCCTTGTGAGACAAA
AGTTGATGCTGTCGTGTAACTTGAGAGCTTTTTGGGGTCTAAGTTTTCGTTATTACTGACTCTATTCGCAAGCATTATTT
GGCATGAACGAACCATCACGCTCTTGACACACAGTATTCTTGACACTTTGAACCCTGAGCCTAAACTGGAGGCGTCTATA
ACTTGTGATATACGCAGGGTCCTTTCACGTTTGCTAGGGGACCCTGTTTGTGTCTAGTTGCTAACTGTTACTTGCTGGGA
A
```

Figure S7. Maximum-likelihood tree based on the Tef1 DNA sequences of the species analysed in Fig. 4. Branch support analysis was evaluated by 1000 ultrafast bootstrap replicates. Some nodes of the tree were not well supported given the low divergence (mean 1.5%) and the length of the alignment (1411 positions, 43 parsimony informative).

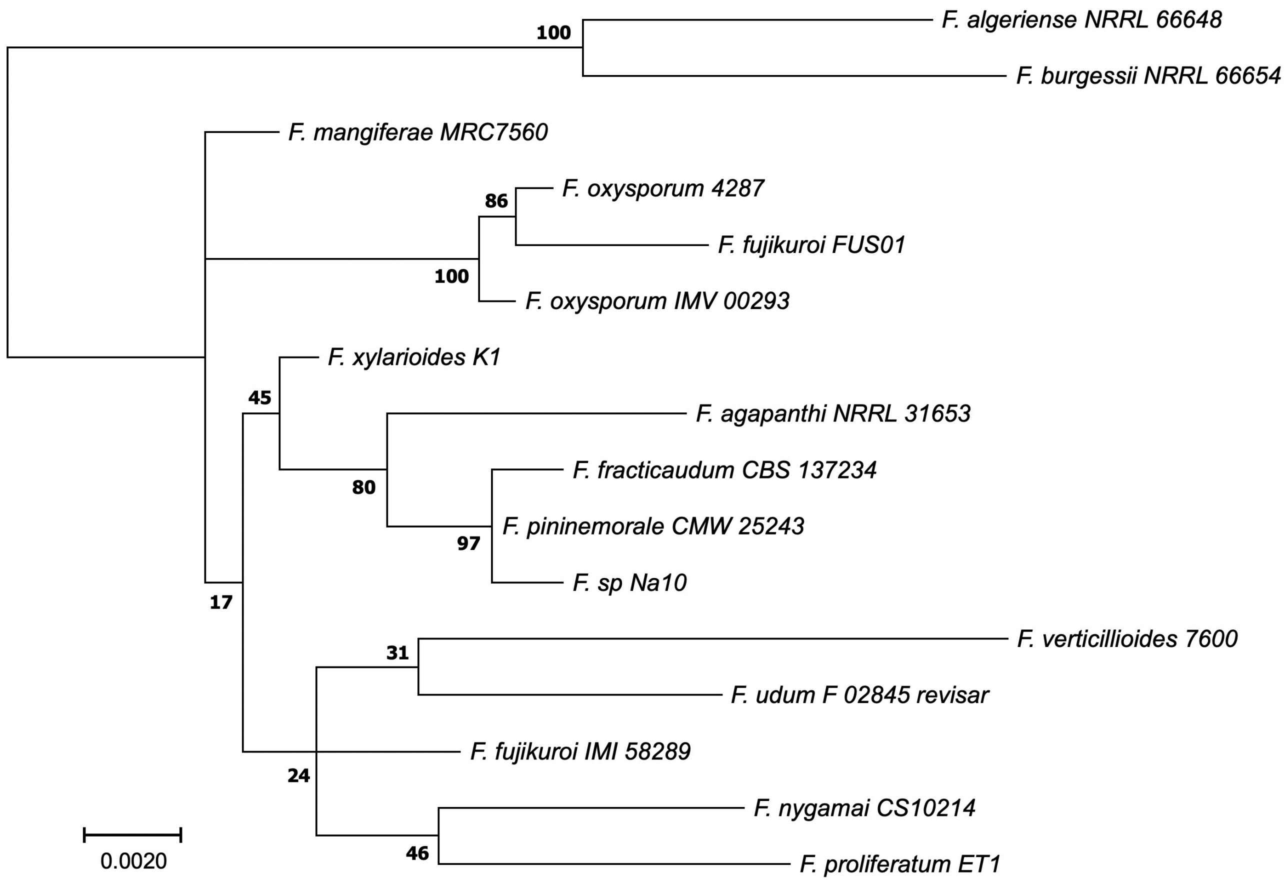

Figure S8. Consensus structure between the *carP* sequences compared in Fig. S7, obtained with the locARNA method (Will et al. 2012). The predicted secondary structure is represented below with the VARNA software (http://varna.lri.fr).

(((((...(((..((((((((((((..((((.(((.......)))))))((((((....))))))((((((((((.(((((.((.....)).((((..)
)))...................................(((((((...........(((........)))....................................((....)).........(((((.....))))).......)))))))...........
...))))))))).................................(((((.((.((((.(((.(((.((((((...(.((((((...................((((.(((((((.........((((((....))).)))..............
....((((((((((.....))))))..........................((((.(((...(((((
(((................))))))))...)......))......((((((((............))))).)))...........................((((.((...((((((..
.........))))).)).))))..((((.....))))....((..(((((((((((............(((.....))).........))))))..........)))))))......))))..(
((..............)))......)))))..................(((((((.((((((....................................))))).(((.(((((........))))))))................................
............((((((((.(((((....))))))))........)))))))))))))..................................(((((....)))))......................))))).)).)).......)).......))))..
.)).)...))).........))))))..))).))))))))))).))))))..)))))))....))))).)))....)))))(((.((((((((.(((((.((((.....)))).))..
...................................(((.....))).........))))))....))))))))..((((((((((....(....))))))))...)))(((.........)))((((((((((..((....)))))))))))).((((
((...(..(((.((...((.....)))).))))...))))))

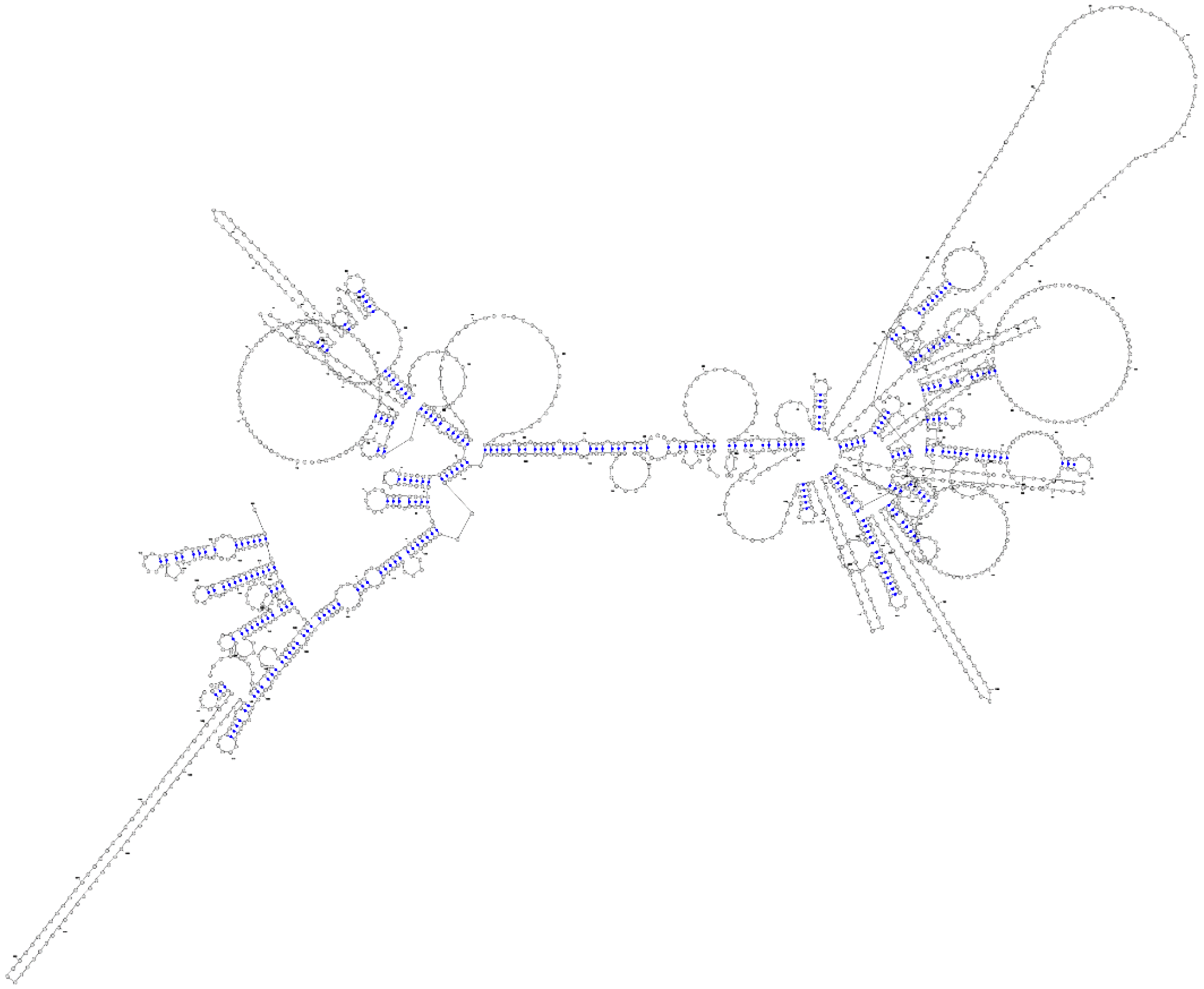

Reference:

Will S, Joshi T, Hofacker IL, et al (2012) LocARNA-P: accurate boundary prediction and improved detection of structural RNAs. RNA 18:900–914.

Figure S9. Transcripts associated to protein-coding genes in the *carS* region of several *Fusarium* species. Data correspond to syntenic sequences referred to *Fusarium oxysporum* f. sp. *lycopersici* 4287, as shown in the Browser FungiDB with the available *Fusarium* genomes. *carP* sequences are shown as an empty brown arrow in the species in which it could be identified. Information on the search and precise locations of these *carP* sequences is indicated in the following table. Three small *carP* homologous DNA segments were found in *F. graminearum*.

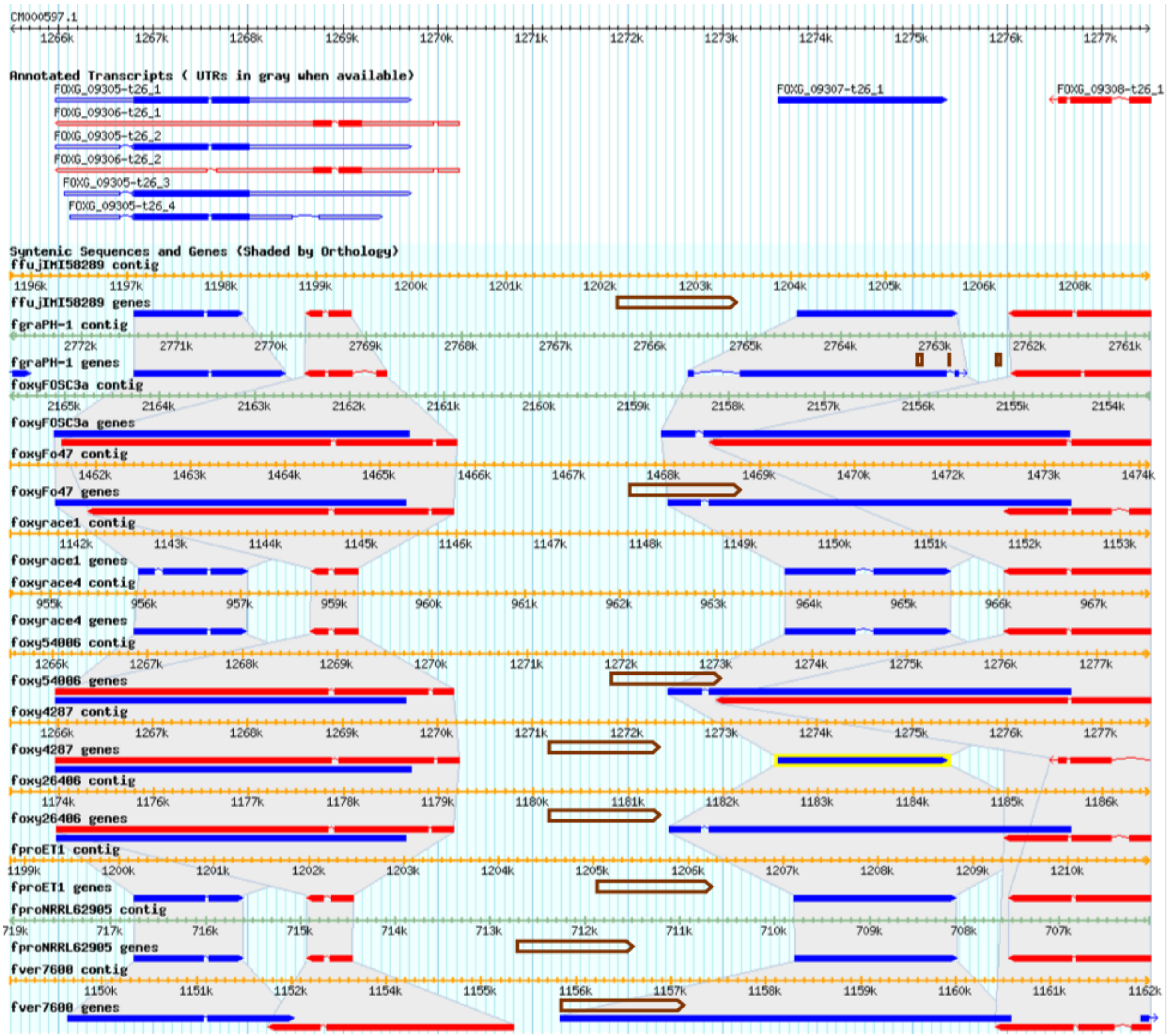

Figure S9 (cont.).

species	contig	start	end	strand	status
Fusarium_fujikuroi_IMI_58289	HF679029	1202162	1203429	+	complete
Fusarium_graminearum_PH-1	HG970334	2762301	2762357	-	fragmented
	HG970334	2763133	2763201	-	
	HG970334	2762854	2762876	-	
F_oxysporum_FOSC3e	NOT PRESENT				
Fusarium_oxysporum_Fo47	JH717904.1	1467616	1468808	+	complete
F_oxysporum_race1	NOT PRESENT				
F_oxysporum_race4	NOT PRESENT				
Fusarium_oxysporum_f._sp._cubense _tropical_race_4_54006	JH658277.1	1271874	1273067	+	complete
Fusarium_oxysporum_f._sp._lycopersici_4287	CM000597.1	1271165	1272359	+	complete
Fusarium_oxysporum_f._sp._melonis_26406	JH659333.1	1180160	1181355	+	complete
Fusarium_proliferatum_ET1	FJOF01000005	1205057	1206277	+	complete
Fusarium_proliferatum_strain_NRRL62905	FCQG01000008	712747	711481	+	complete
Fusarium_verticillioides_7600	CM000584.1	1155827	1157160	+	complete

Figure S10. Effect of *carP* deletion on transcript levels of photoreceptor genes *wc1/wocA*, *cryD* and *vvdA* in *F. oxysporum* (A) and *F. fujikuroi* (B) grown in the dark or exposed for one hour to light. The analysis in *F. oxysporum* was extended to the gene Foxg_01269, orthologous to *con-10* of *N. crassa*. The results show the mean and standard error of RT-qPCR data from three independent experiments. The relative RNA levels are referred to the RNA content of the wild strain in darkness.

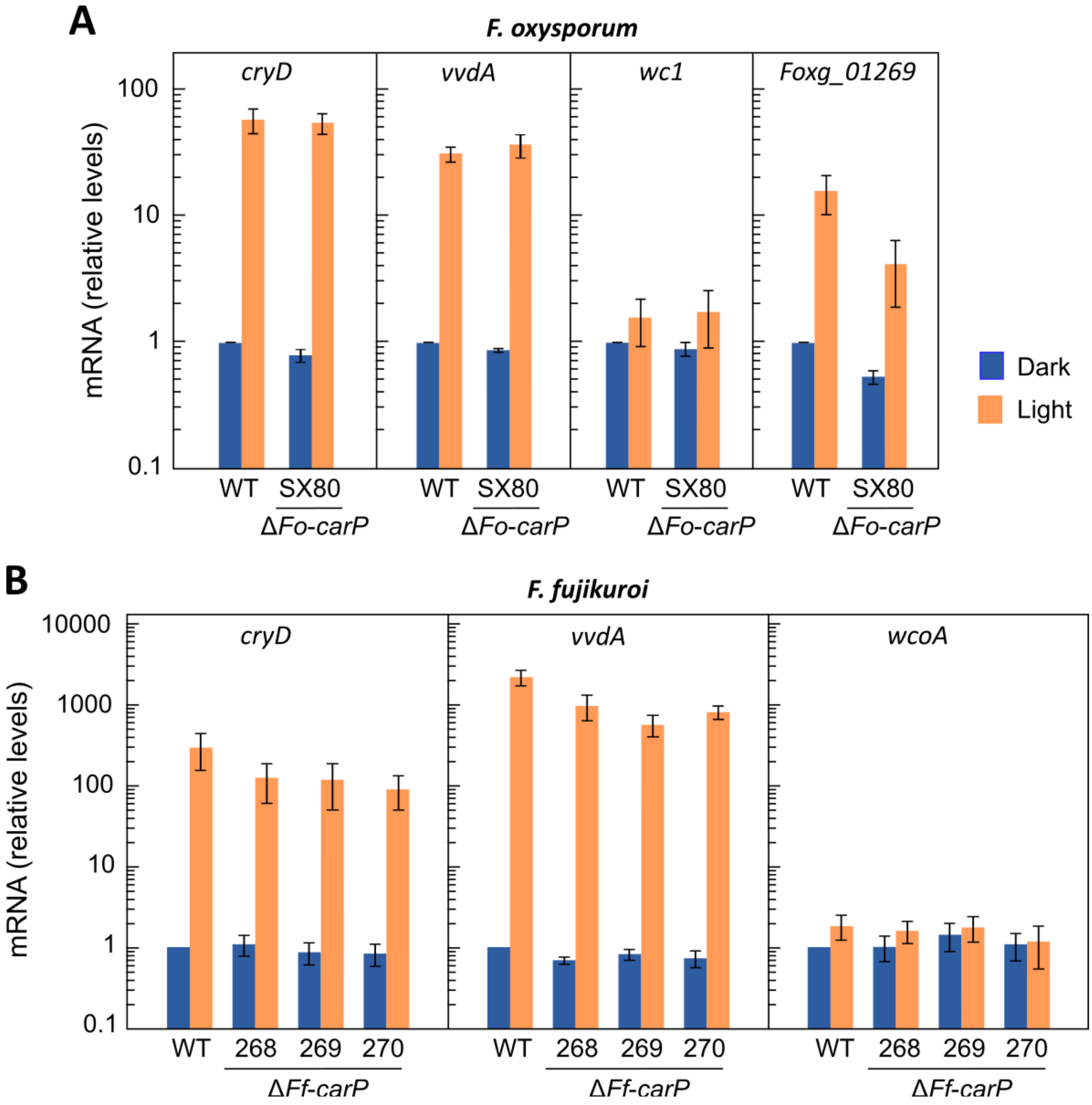

Figure S11. Molecular analysis of deletion of the *carP** and *carP* sequences in the *F. oxysporum* wild strain by replacement with a Hyg^{R} cassette (*hph* gene). A physical map of the genomic region where *carP* and its neighbouring genes are located is shown in the central scheme (Middle map in **C**). *carP* and the overlapping *carP** segment are indicated in pale blue. The maps for the same region in the Δ*carP* (above) and Δ*carP** (below) transformants are shown. Candidate transformants were checked for the correct replacement by PCR (**A** and **D** panels) and Southern blot (**B** and **E**). Cropped lanes are separated by a black line. Full-length blots/gels are presented in **F**.

PCR interpretation: External primers used to amplify the relevant DNA segment are indicated on the map (**C**) with green or red arrowheads, and the corresponding products as green or red dotted lines. SM: Size markers. PS: Primer set. Wt: wild type. In panel A, only transformant #3 exhibited the slight band shift found in the pDul14 plasmid, used in the transformation. In Panel D, transformants #2, 3, 4, 5, and 8 exhibited the band pattern expected for the correct replacement.

Southern blot interpretation: *Ava*I restriction sites are indicated as A. Hybridization probe is indicated as a blue bar and expected hybridization products as blue lines. In panel B, the only transformant with the expected band in the PCR exhibits the band expected for the correct *carP* replacement. In Panel E, two transformants with the expected band in the PCR test exhibits the hybridizing band expected for the correct *carP** replacement.

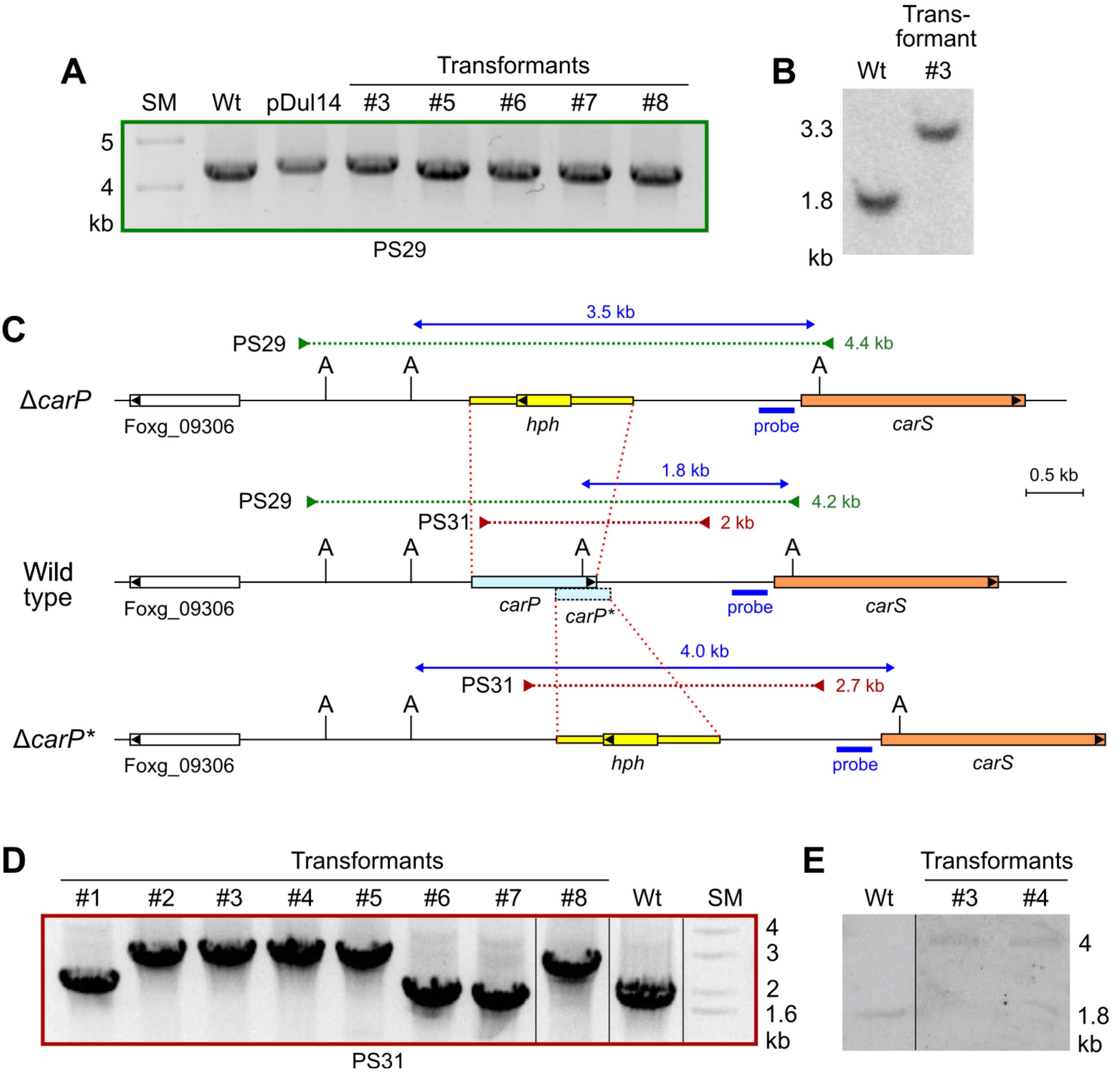

F

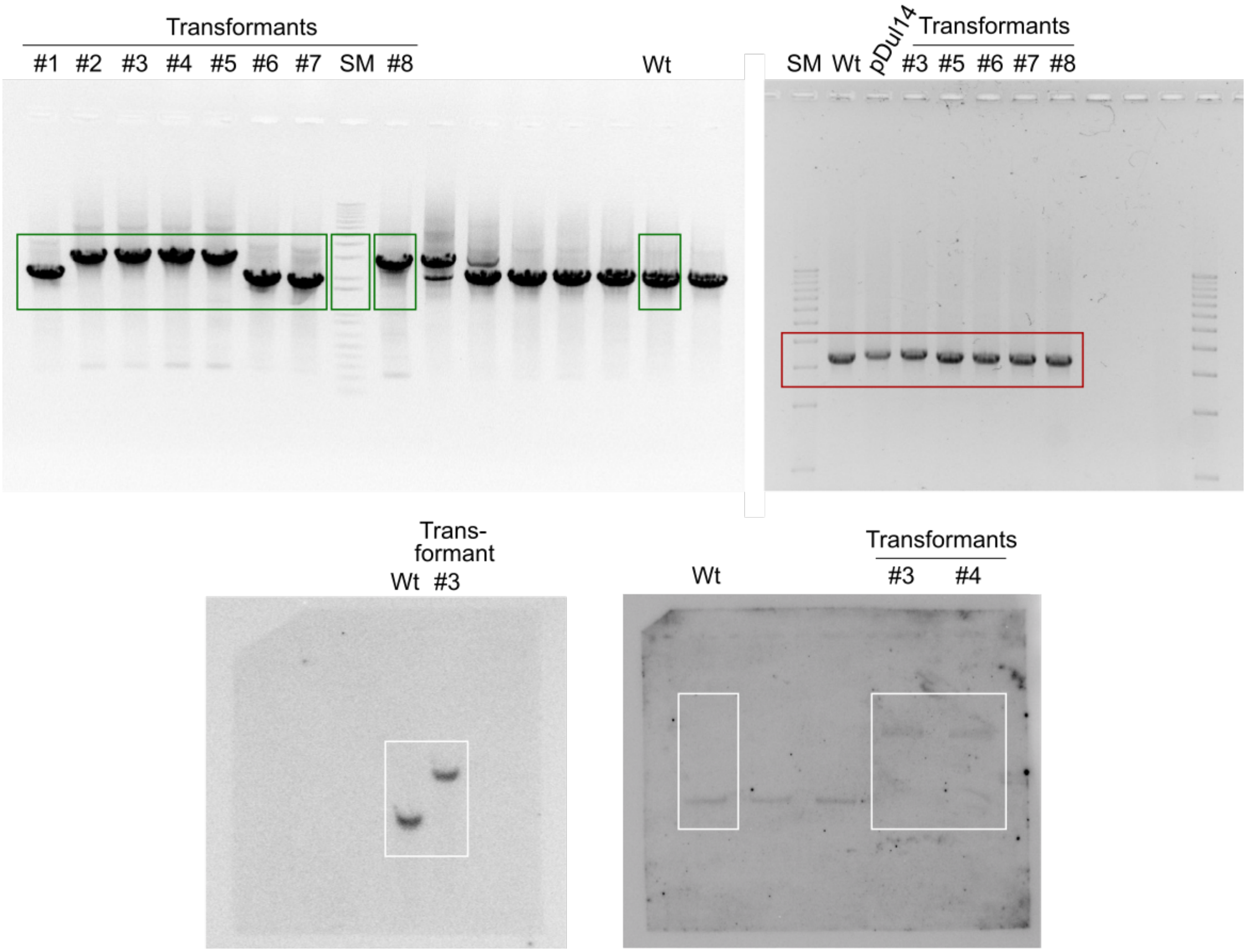

Transformants
#1 #2 #3 #4 #5 #6 #7 SM #8
Wt
SM Wt pDul14
Transformants
#3 #5 #6 #7 #8
Trans-
formant
Wt #3
Wt
Transformants
#3 #4

Figure S12. Molecular analysis of deletion of the *carP* sequence in the *F. fujikuroi* wild strain by replacement with a Hyg^R cassette (*hph* gene). A physical map of the genomic region where *carP* (pale blue) and its neighbouring genes are located is shown in the scheme (Upper map in **A**). The map for the same region in the Δ*carP* transformants is shown below. Candidate transformants were checked for the correct replacement by PCR (**B**) and Southern blot (**C**). Full-length blots/gels are presented in **D** and **E**.

PCR interpretation: Primer sets (PS) used to amplify relevant regions are indicated on the map with coloured arrowheads at the extremes of the corresponding products, indicated as coloured dotted lines. SM: Size markers. C: DNA-free control. P: plasmid pcarPhyg. Wt: wild type. In panel B, transformants #1, 2, 4, and 10 exhibited the band pattern expected for the correct replacement.

Southern blot interpretation: *Eco*RI and *Xho*I restriction sites are indicated as E and X. Hybridization probe is indicated as a blue bar and expected hybridization products as blue lines. In panel C, three transformants with the expected band in the PCR, #1, 2 and 4, exhibit the band corresponding to the correct *carP* replacement.

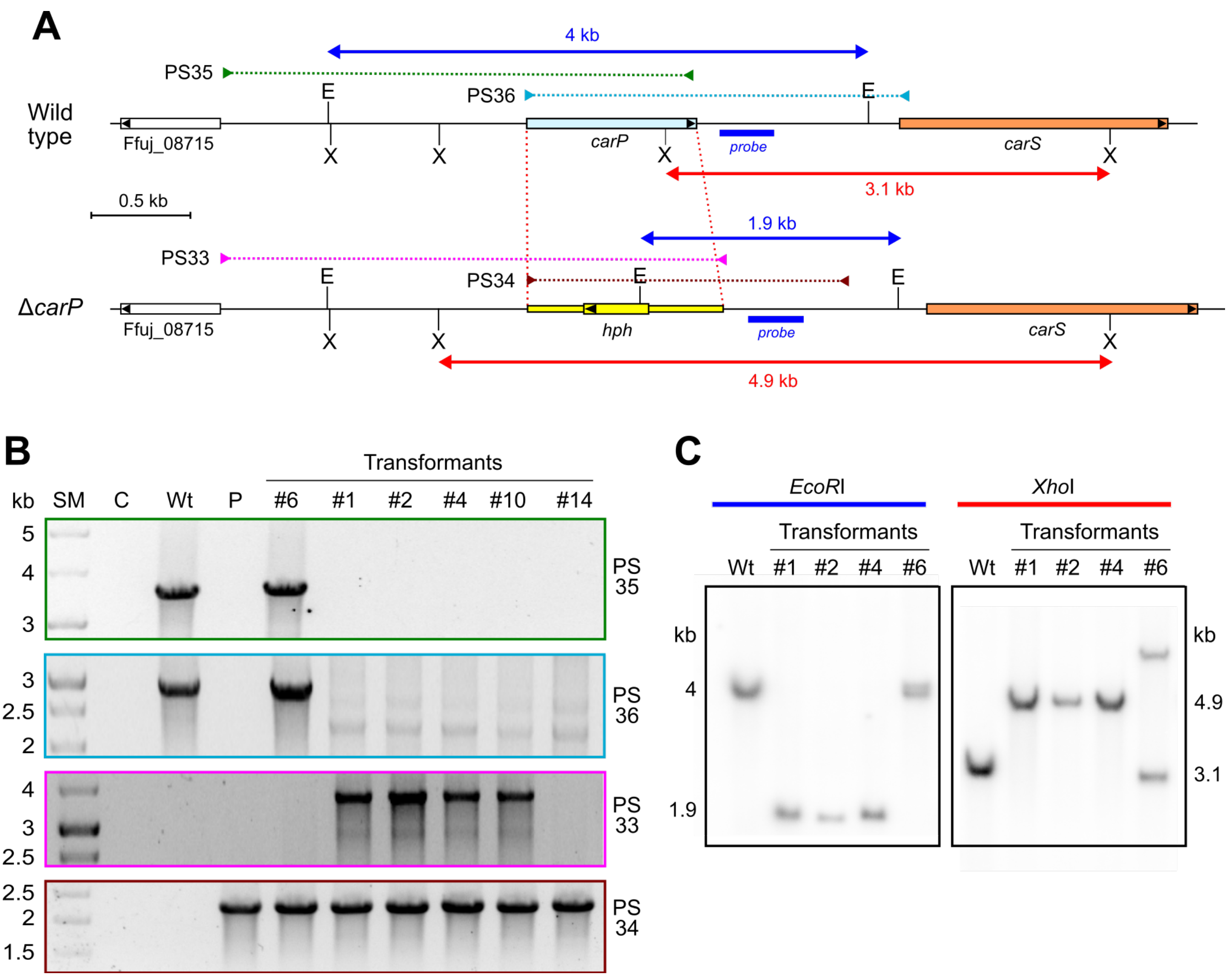

D

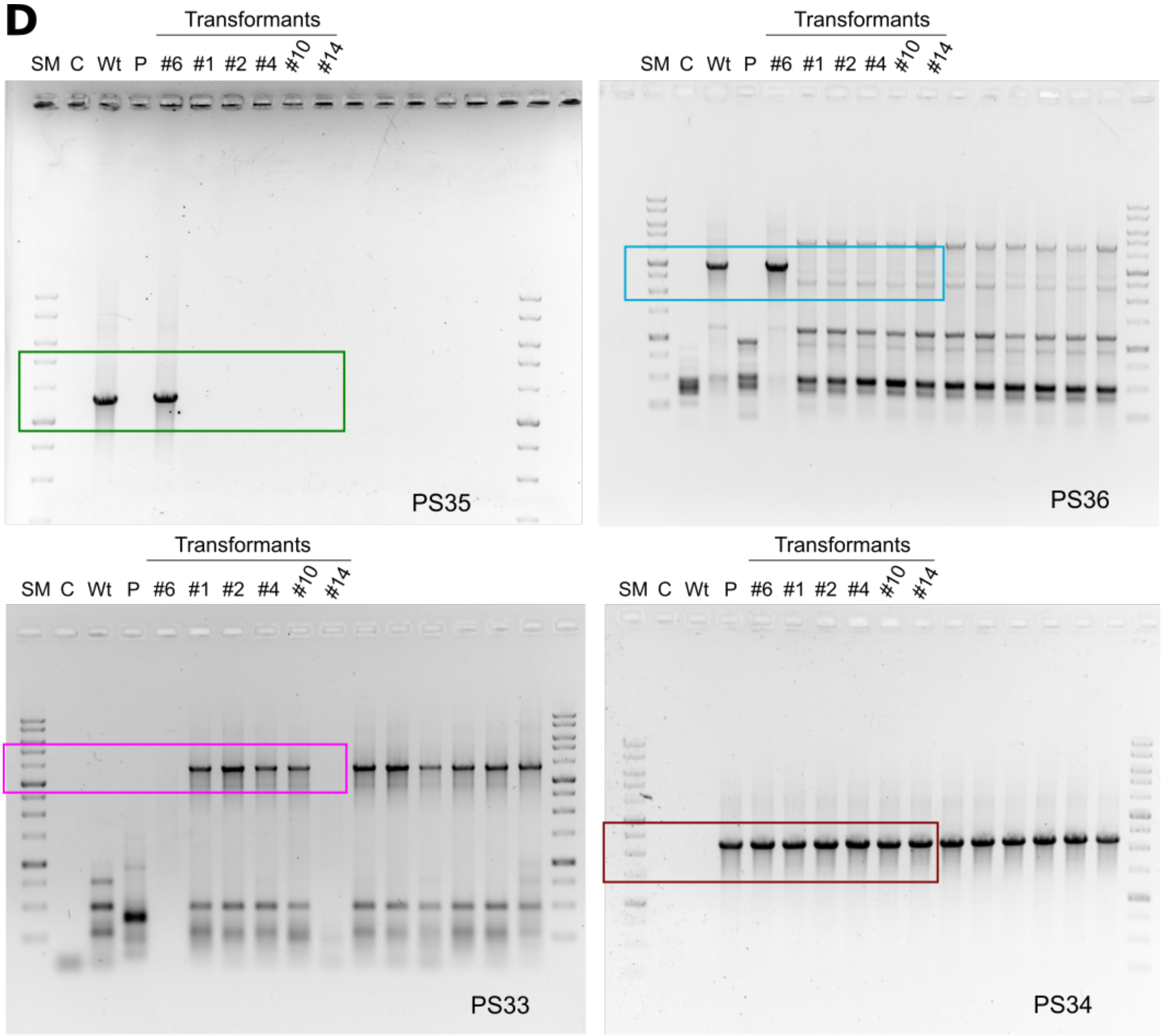

Transformants
SM C Wt P #6 #1 #2 #4 #10 #14
PS35
Transformants
SM C Wt P #6 #1 #2 #4 #10 #14
PS36
Transformants
SM C Wt P #6 #1 #2 #4 #10 #14
PS33
Transformants
SM C Wt P #6 #1 #2 #4 #10 #14
PS34

E

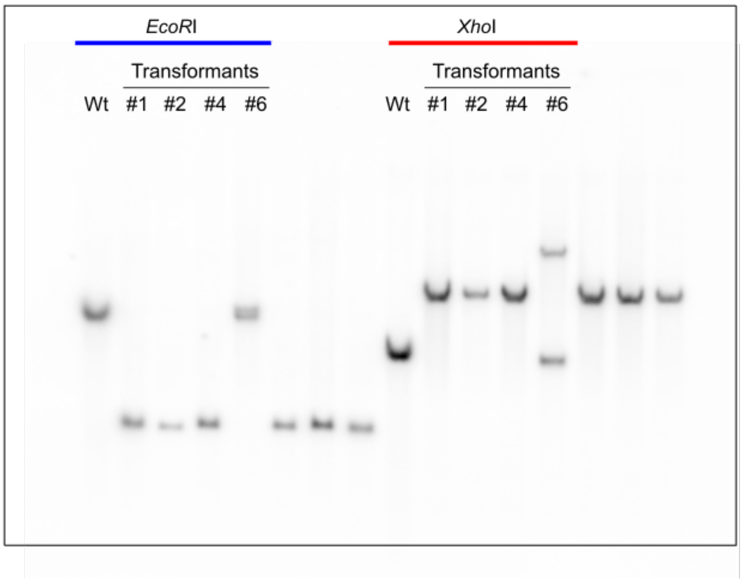

*Eco*RI
Transformants
Wt #1 #2 #4 #6
*Xho*I
Transformants
Wt #1 #2 #4 #6

Figure S13. Pictures of original gels for panels displayed in Figures 2 and 5. Lanes used from each gel are labelled above.

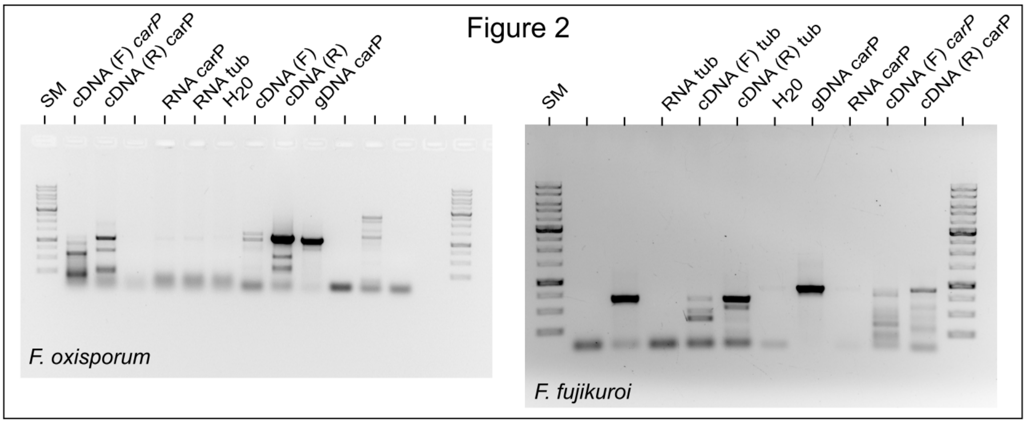

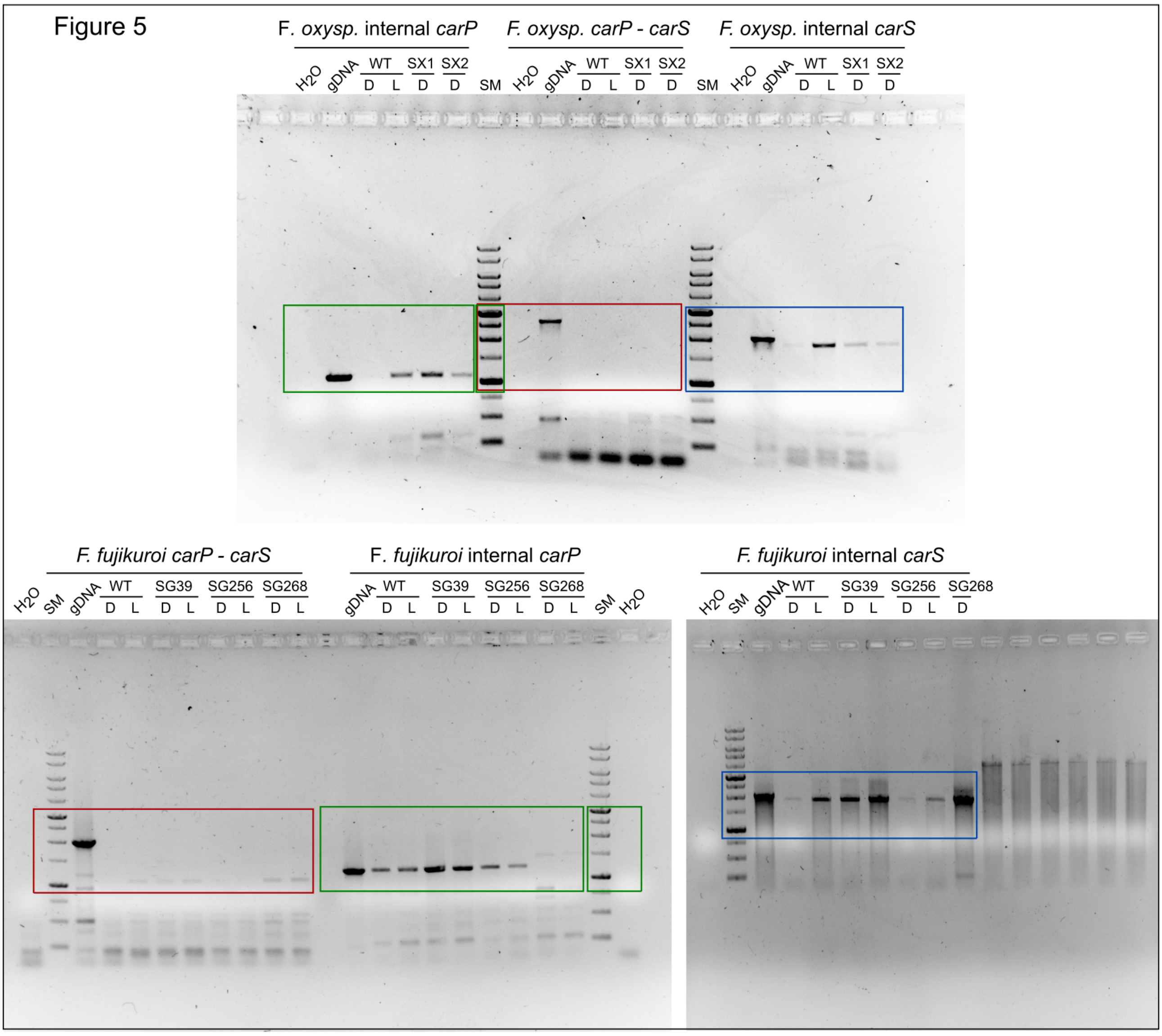

Table S1. Test of *Fo-carP* coding capacity through CPC method (Kong et al. 2007). None of the tested *carP* sequences fit the conditions for coding functions.

ID	C/NC	Coding potential score
(revcomp)carP_F_nygamai_CS10214	noncoding	-1.45891
(revcomp)carP_F_fujikuroi_IMI_58289	noncoding	-1.45322
(revcomp)carP_F_mangiferae_MRC7560	noncoding	-1.43248
(revcomp)carP_F_proliferatum_ET1	noncoding	-1.43141
carP_F_verticillioides_7600	noncoding	-1.41673
(revcomp)carP_F_verticillioides_7600	noncoding	-1.38289
carP_F_proliferatum_ET1	noncoding	-1.31366
carP_F_sp_Na10	noncoding	-1.27707
(revcomp)carP_F_algeriense_NRRL_66648	noncoding	-1.25455
carP_F_fujikuroi_FUS01	noncoding	-1.24671
carP_F_oxysporum_00293	noncoding	-1.24505
(revcomp)carP_F_xylarioides_K1	noncoding	-1.23913
(revcomp)carP_F_burgessii_NRRL_66654	noncoding	-1.23374
carP_F_burgessii_NRRL_66654	noncoding	-1.22717
(revcomp)carP_F_agapanthi_NRRL_31653	noncoding	-1.22276
carP_F_pininemorale_CMW	noncoding	-1.18521
carP_F_xylarioides_K1	noncoding	-1.18359
carP_F_fujikuroi_IMI_58289	noncoding	-1.17698
(revcomp)carP_F_fracticaudum_CBS	noncoding	-1.17189
carP_Fo	noncoding	-1.16931
(revcomp)carP_F_pininemorale_CMW	noncoding	-1.12739
carP_F_nygamai_CS10214	noncoding	-1.12625
(revcomp)carP_F_udum_F_02845	noncoding	-1.11736
carP_F_fracticaudum_CBS	noncoding	-1.0927
carP_F_agapanthi_NRRL_31653	noncoding	-1.08856
carP_F_algeriense_NRRL_66648	noncoding	-1.04656
(revcomp)carP_F_sp_Na10	noncoding	-1.02215
carP_F_udum_F_02845	noncoding	-1.00673
carP_F_mangiferae_MRC7560	noncoding (weak)	-0.999518
(revcomp)carP_F_fujikuroi_FUS01	noncoding (weak)	-0.940017
(revcomp)carP_F_oxysporum_00293	noncoding (weak)	-0.938433
(revcomp)carP_Fo	noncoding (weak)	-0.928604

Reference:

Kong L, Zhang Y, Ye Z-Q, et al (2007) CPC: assess the protein-coding potential of transcripts using sequence features and support vector machine. Nucleic Acids Res 35:W345-349.

Table S2. Test of *Fo-carP* coding capacity through CPC2 method (Kang et al. 2017). With the exception of *F. verticillioides*, none of the tested *carP* sequences fits the conditions for coding functions. In the case of *F. verticillioides*, there is an ORF coding for 200 amino acids with negative results both in searches with blastp in NCBI or through Interproscan for the presence of protein domains.

ID	Label	Coding probability	Peptide length(aa)	Fickett score	Isoelectric point	ORF integrity
carP_F_agapanthi_NRRL_31653	noncoding	0.0638958	74	0.3684	7.8147583	complete
carP_F_algeriense_NRRL_66648	noncoding	0.0836067	93	0.3111	10.9751587	complete
carP_F_burgessii_NRRL_66654	noncoding	0.0596944	80	0.33524	11.8445435	complete
carP_F_fracticaudum_CBS	noncoding	0.0436296	61	0.35357	11.2278442	complete
carP_F_fujikuroi_FUS01	noncoding	0.0858631	86	0.34657	11.4961548	complete
carP_F_fujikuroi_IMI_58289	noncoding	0.11919	108	0.28542	8.23492432	complete
carP_F_mangiferae_MRC7560	noncoding	0.0370059	59	0.36706	6.01104736	complete
carP_F_nygamai_CS10214	noncoding	0.103646	107	0.27674	8.76934814	complete
carP_Fo	noncoding	0.472934	132	0.35616	9.15484619	complete
carP_F_oxysporum_00293	noncoding	0.375419	133	0.33306	8.99163818	complete
carP_F_pininemorale_CMW	noncoding	0.109845	74	0.34055	4.87689209	complete
carP_F_proliferatum_ET1	noncoding	0.0374705	60	0.33021	5.46099854	complete
carP_F_sp_Na10	noncoding	0.0761937	83	0.33167	10.3644409	complete
carP_F_udum_F_02845	noncoding	0.132091	101	0.33795	11.0901489	complete
carP_F_verticillioides_7600	coding	0.733734	200	0.29216	9.09442139	complete
carP_F_xylarioides_K1	noncoding	0.0214854	43	0.30796	12.3058472	complete

Reference:

Kang Y-J, Yang D-C, Kong L, et al (2017) CPC2: a fast and accurate coding potential calculator based on sequence intrinsic features. Nucleic Acids Res 45:W12–W16.

Table S3. Identity of *carP* sequences from other *Fusarium* species compared to *Fo-carP*. As observed here, and in Figs S6 and S7, the strain FUS01 of *F. fujikuroi* has been very probably a wrong species assignation of a *F. oxysporum* strain.

Species	Max score	Total score	Query cover	E	Identity
carP_F_fujikuroi_FUS01	2087	2087	100%	0	98.83%
carP_F_oxysporum_00293	2008	2008	100%	0	97.08%
carP_F_xylarioides_K1	1120	1120	99%	0	79.20%
carP_F_udum_F_02845	1114	1114	100%	0	78.85%
carP_F_proliferatum_ET1	943	943	100%	0	74.98%
carP_F_nygamai_CS10214	927	927	100%	0	74.67%
carP_F_mangiferae_MRC7560	900	900	100%	0	74.28%
carP_F_fracticaudum_CBS 137234	891	891	100%	0	73.56%
carP_F_pininemorale_CMW 25243	874	874	100%	0	73.06%
carP_F_agapanthi_NRRL_31653	868	868	100%	0	72.83%
carP_F_sp_Na10	838	838	100%	0	72.16%
carP_F_fujikuroi_IMI_58289	805	805	94%	0	72.95%
carP_F_algeriense_NRRL_66648	592	592	100%	8.00E-172	70.76%
carP_F_burgessii_NRRL_66654	588	588	100%	3.00E-170	70.50%
carP_F_verticillioides_7600	548	711	87%	3.00E-158	71.07%

Table S4. Positive hits after a blastn search of *Fo-carP* against the 249 Fusarium WGS available at NCBI. Selection criteria for positive hits were E <0.001, and query cover >25%.

Select for downloading or viewing reports	Description	Max Score	Total Score	Query Cover	E value	Per. Ident	Accession
RBXW01000013.1	Fusarium oxysporum f. sp. lycopersici strain race 3 isolate D11 chromosome 9, whole genome shotgun sequence	2156	2156	100%	0	100.00%	RBXW01000013.1
QESU01000034.1	Fusarium oxysporum f. sp. lycopersici 4287 C_9.4, whole genome shotgun sequence	2156	2156	100%	0	100.00%	QESU01000034.1
MAMG01000084.1	Fusarium oxysporum f. sp. lycopersici strain Fol002 Fol002_contig_84, whole genome shotgun sequence	2156	2156	100%	0	100.00%	MAMG01000084.1
MALW01000063.1	Fusarium oxysporum f. sp. lycopersici 4287 Fol4287illumina_contig_63, whole genome shotgun sequence	2156	2156	100%	0	100.00%	MALW01000063.1
MALV01000433.1	Fusarium oxysporum f. sp. lycopersici 4287 Fol4287_iont_contig_435, whole genome shotgun sequence	2156	2156	100%	0	100.00%	MALV01000433.1
MALS01000302.1	Fusarium oxysporum f. sp. lycopersici strain Fol074 Fol074_contig_302, whole genome shotgun sequence	2156	2156	100%	0	100.00%	MALS01000302.1
MALR01000315.1	Fusarium oxysporum f. sp. lycopersici strain Fol073 Fol073_contig_315, whole genome shotgun sequence	2156	2156	100%	0	100.00%	MALR01000315.1
MALO01000148.1	Fusarium oxysporum f. sp. lycopersici strain Fol038 Fol038_contig_148, whole genome shotgun sequence	2156	2156	100%	0	100.00%	MALO01000148.1
MALN01000096.1	Fusarium oxysporum f. sp. lycopersici strain Fol029 Fol029_contig_96, whole genome shotgun sequence	2156	2156	100%	0	100.00%	MALN01000096.1
MALL01000022.1	Fusarium oxysporum f. sp. lycopersici strain Fol018 Fol018_contig_22, whole genome shotgun sequence	2156	2156	100%	0	100.00%	MALL01000022.1
MALK01000081.1	Fusarium oxysporum f. sp. lycopersici strain Fol026 Fol026_contig_81, whole genome shotgun sequence	2156	2156	100%	0	100.00%	MALK01000081.1
MALJ01000018.1	Fusarium oxysporum f. sp. lycopersici strain Fol014 Fol014_contig_18, whole genome shotgun sequence	2156	2156	100%	0	100.00%	MALJ01000018.1
MALI01000137.1	Fusarium oxysporum f. sp. lycopersici strain Fol007 Fol007illumina_contig_137, whole genome shotgun sequence	2156	2156	100%	0	100.00%	MALI01000137.1
MALH01000116.1	Fusarium oxysporum f. sp. lycopersici strain Fol004 Fol004_contig_116, whole genome shotgun sequence	2156	2156	100%	0	100.00%	MALH01000116.1
AAXH01000589.1	Fusarium oxysporum f. sp. lycopersici 4287 chromosome 9 cont2.589, whole genome shotgun sequence	2156	2156	100%	0	100.00%	AAXH01000589.1
NJBV01000031.1	Fusarium oxysporum strain KOD887 Fophy_KOD886_contig_31, whole genome shotgun sequence	2151	2151	100%	0	99.92%	NJBV01000031.1
NJBU01000114.1	Fusarium oxysporum strain KOD888 Fophy_KOD887_contig_114, whole genome shotgun sequence	2151	2151	100%	0	99.92%	NJBU01000114.1
NJBT01000028.1	Fusarium oxysporum strain Tu58 Fophy_KOD888_contig_28, whole genome shotgun sequence	2151	2151	100%	0	99.92%	NJBT01000028.1
NJCY01000008.1	Fusarium oxysporum f. sp. melonis 26406 Fom001_contig_7, whole genome shotgun sequence	2140	2140	100%	0	99.75%	NJCY01000008.1
AGNE01000072.1	Fusarium oxysporum f. sp. melonis 26406 cont1.72, whole genome shotgun	2140	2140	100%	0	99.75%	AGNE01000072.1

	sequence						
NJBS01000012.1	Fusarium oxysporum f. sp. tulipae strain Tu67 Fotul_Tu67_contig_12, whole genome shotgun sequence	2116	2116	100%	0	99.33%	NJBS01000012.1
LSNI01000177.1	Fusarium oxysporum f. sp. medicaginis isolate Fom-5190a contig_177, whole genome shotgun sequence	2105	2105	100%	0	99.16%	LSNI01000177.1
NJBW01000006.1	Fusarium oxysporum strain KOD886 Fonon_Tu58_contig_6, whole genome shotgun sequence	2103	2103	100%	0	99.08%	NJBW01000006.1
AGNB01000077.1	Fusarium oxysporum f. sp. radicis-lycopersici 26381 cont1.77, whole genome shotgun sequence	2101	2101	100%	0	99.08%	AGNB01000077.1
NJBZ01000115.1	Fusarium oxysporum f. sp. nicotianae strain FON-1 Fonic_001_contig_115, whole genome shotgun sequence	2096	2096	100%	0	99.00%	NJBZ01000115.1
NJBY01000043.1	Fusarium oxysporum f. sp. nicotianae strain 10913 Fonic_003_contig_43, whole genome shotgun sequence	2096	2096	100%	0	99.00%	NJBY01000043.1
NJBX01000052.1	Fusarium oxysporum f. sp. nicotianae strain Ft-Rob Fonic_010_contig_52, whole genome shotgun sequence	2096	2096	100%	0	99.00%	NJBX01000052.1
MRDA01000004.1	Fusarium oxysporum strain Fo_PG contig_4, whole genome shotgun sequence	2096	2096	100%	0	99.00%	MRDA01000004.1
MQTW01000055.1	Fusarium oxysporum f. sp. narcissi strain N139 contig_55, whole genome shotgun sequence	2096	2096	100%	0	99.00%	MQTW01000055.1
MALT01000032.1	Fusarium oxysporum f. sp. lycopersici strain Fol075 Fol075_contig_32, whole genome shotgun sequence	2096	2096	100%	0	99.00%	MALT01000032.1
MALQ01000096.1	Fusarium oxysporum f. sp. lycopersici strain Fol072 Fol072_contig_96, whole genome shotgun sequence	2096	2096	100%	0	99.00%	MALQ01000096.1
MALP01000118.1	Fusarium oxysporum f. sp. lycopersici strain Fol069 Fol069_contig_118, whole genome shotgun sequence	2096	2096	100%	0	99.00%	MALP01000118.1
MALM01000268.1	Fusarium oxysporum f. sp. lycopersici strain Fol016 Fol016_contig_268, whole genome shotgun sequence	2096	2096	100%	0	99.00%	MALM01000268.1
AGBH01000064.1	Fusarium oxysporum f. sp. lycopersici MN25 cont1.64, whole genome shotgun sequence	2096	2096	100%	0	99.00%	AGBH01000064.1
NJCV01000056.1	Fusarium oxysporum f. sp. narcissi strain Na5 Fonar_Na5_contig_56, whole genome shotgun sequence	2092	2092	100%	0	98.91%	NJCV01000056.1
NJCL01000082.1	Fusarium oxysporum f. sp. gladioli strain G2 Fogla_G2_contig_82, whole genome shotgun sequence	2090	2090	100%	0	98.83%	NJCL01000082.1
MABS01000058.1	Fusarium oxysporum f. sp. radicis-cucumerinum strain Forc031 Forc031_contig_58, whole genome shotgun sequence	2090	2090	100%	0	98.83%	MABS01000058.1
MABR01000052.1	Fusarium oxysporum f. sp. radicis-cucumerinum strain Forc024 Forc024_contig_52, whole genome shotgun sequence	2090	2090	100%	0	98.83%	MABR01000052.1
MABQ02000007.1	Fusarium oxysporum f. sp. radicis-cucumerinum strain Forc016 chromosome 9, whole genome shotgun sequence	2090	2090	100%	0	98.83%	MABQ02000007.1
NCQQ02000003.1	Fusarium fujikuroi strain FUS01 Fusarium_fujikuroi-3-size2100021, whole genome shotgun sequence	2087	2087	100%	0	98.83%	NCQQ02000003.1
MRCZ01000055.1	Fusarium oxysporum strain Fo_CB3 contig_55, whole genome shotgun sequence	2087	2087	100%	0	98.83%	MRCZ01000055.1

MRCY01000001.1	Fusarium oxysporum strain Fo_A28 contig_1, whole genome shotgun sequence	2087	2087	100%	0	98.83%	MRCY01000001.1
MRCW01000010.1	Fusarium oxysporum f. sp. cepae strain FoC_A23 contig_10, whole genome shotgun sequence	2087	2087	100%	0	98.83%	MRCW01000010.1
MRCV01000043.1	Fusarium oxysporum f. sp. cepae strain FoC_125 contig_43, whole genome shotgun sequence	2087	2087	100%	0	98.83%	MRCV01000043.1
MRCU01000007.1	Fusarium oxysporum f. sp. cepae strain FoC_Fus2 chromosome 9, whole genome shotgun sequence	2087	2087	100%	0	98.83%	MRCU01000007.1
JNNQ01001109.1	Fusarium oxysporum strain UASWS AC1 Contig1109, whole genome shotgun sequence	2087	2087	100%	0	98.83%	JNNQ01001109.1
QUXA01000013.1	Fusarium oxysporum strain ISS-F4 Fusariumoxysporum_strainISSF4-22025, whole genome shotgun sequence	2085	2085	100%	0	98.83%	QUXA01000013.1
QUWZ01000011.1	Fusarium oxysporum strain ISS-F3 Fusariumoxysporum_strainISSF3-18464, whole genome shotgun sequence	2085	2085	100%	0	98.83%	QUWZ01000011.1
MALU01000008.1	Fusarium oxysporum strain FoMN14 FoMN14_contig_8, whole genome shotgun sequence	2085	2085	100%	0	98.83%	MALU01000008.1
AFMM01000085.1	Fusarium oxysporum Fo47 cont1.85, whole genome shotgun sequence	2085	2085	100%	0	98.83%	AFMM01000085.1
NJCU01000077.1	Fusarium oxysporum f. sp. nicotianae strain Ft-1512 Fonic_012_contig_77, whole genome shotgun sequence	2072	2072	100%	0	98.58%	NJCU01000077.1
NJCF01000001.1	Fusarium oxysporum f. sp. lilii strain Fol39 Folil_Fol39_contig_1, whole genome shotgun sequence	2072	2072	100%	0	98.58%	NJCF01000001.1
MABO01000306.1	Fusarium oxysporum f. sp. cucumerinum strain Foc035 Foq035_contig_306, whole genome shotgun sequence	2072	2072	100%	0	98.58%	MABO01000306.1
RBCA01000166.1	Fusarium oxysporum strain MOD1-FUNGI16 MOD1-FUNGI16_166_length_69382_cov_102.517192, whole genome shotgun sequence	2067	2067	100%	0	98.49%	RBCA01000166.1
NRIA02000062.1	Fusarium oxysporum f. sp. conglutinans strain FGL03-6 Scaffold11_3, whole genome shotgun sequence	2067	2067	100%	0	98.49%	NRIA02000062.1
NRHZ01000012.1	Fusarium oxysporum f. sp. conglutinans strain 58385 Scaffold12, whole genome shotgun sequence	2067	2067	100%	0	98.49%	NRHZ01000012.1
NJCJ01000131.1	Fusarium oxysporum f. sp. lagenariae strain 01-03008 Folag_001_contig_131, whole genome shotgun sequence	2067	2067	100%	0	98.49%	NJCJ01000131.1
NJCI01000170.1	Fusarium oxysporum f. sp. lagenariae strain 03-05118 Folag_002_contig_170, whole genome shotgun sequence	2067	2067	100%	0	98.49%	NJCI01000170.1
NJCG01000175.1	Fusarium oxysporum f. sp. lagenariae strain Lag:1-1 Folag_005_contig_175, whole genome shotgun sequence	2067	2067	100%	0	98.49%	NJCG01000175.1
NJCB01000018.1	Fusarium oxysporum f. sp. momordicae strain NRRL26413 Fomom_001_contig_18, whole genome shotgun sequence	2067	2067	100%	0	98.49%	NJCB01000018.1
NJCA01000063.1	Fusarium oxysporum f. sp. momordicae strain 90NF2-1 Fomom_004_contig_63, whole genome shotgun sequence	2067	2067	100%	0	98.49%	NJCA01000063.1
MALG01000016.1	Fusarium oxysporum f. sp. niveum strain Fon021 Fon021blob3c_contig_16, whole genome shotgun sequence	2067	2067	100%	0	98.49%	MALG01000016.1
MALF01000100.1	Fusarium oxysporum f. sp. niveum strain Fon037 Fon037_contig_100, whole genome shotgun sequence	2067	2067	100%	0	98.49%	MALF01000100.1

MALE01000022.1	Fusarium oxysporum f. sp. niveum strain Fon020 Fon020_contig_22, whole genome shotgun sequence	2067	2067	100%	0	98.49%	MALE01000022.1
MALC01000302.1	Fusarium oxysporum f. sp. niveum strain Fon013 Fon013blob2c_contig_302, whole genome shotgun sequence	2067	2067	100%	0	98.49%	MALC01000302.1
MALB01000456.1	Fusarium oxysporum f. sp. niveum strain Fon010 Fon010_contig_456, whole genome shotgun sequence	2067	2067	100%	0	98.49%	MALB01000456.1
MALA01000111.1	Fusarium oxysporum f. sp. niveum strain Fon002 Fon002_contig_111, whole genome shotgun sequence	2067	2067	100%	0	98.49%	MALA01000111.1
MAKZ01000076.1	Fusarium oxysporum f. sp. cucumerinum strain Foc001 Foq001_contig_76, whole genome shotgun sequence	2067	2067	100%	0	98.49%	MAKZ01000076.1
MAKY01000143.1	Fusarium oxysporum f. sp. niveum strain Fon005 Fon005blob3c_contig_143, whole genome shotgun sequence	2067	2067	100%	0	98.49%	MAKY01000143.1
MABP01000183.1	Fusarium oxysporum f. sp. cucumerinum strain Foc037 Foq037_contig_183, whole genome shotgun sequence	2067	2067	100%	0	98.49%	MABP01000183.1
MABN01000345.1	Fusarium oxysporum f. sp. cucumerinum strain Foc030 Foq030blob2c_contig_346, whole genome shotgun sequence	2067	2067	100%	0	98.49%	MABN01000345.1
MABM01000196.1	Fusarium oxysporum f. sp. cucumerinum strain Foc018 Foq018_contig_196, whole genome shotgun sequence	2067	2067	100%	0	98.49%	MABM01000196.1
MABL01000060.1	Fusarium oxysporum f. sp. cucumerinum strain Foc021 Foq021_contig_60, whole genome shotgun sequence	2067	2067	100%	0	98.49%	MABL01000060.1
MABK01000035.1	Fusarium oxysporum f. sp. cucumerinum strain Foc015 Foq015_contig_35, whole genome shotgun sequence	2067	2067	100%	0	98.49%	MABK01000035.1
LPZQ01000239.1	Fusarium oxysporum f. sp. conglutinans strain 1 cENTRYscaffold699_cov430, whole genome shotgun sequence	2067	2067	100%	0	98.49%	LPZQ01000239.1
FMJY01000007.1	Fusarium oxysporum strain V64-1 genome assembly, contig: FRV6_scaffold007, whole genome shotgun sequence	2067	2067	100%	0	98.49%	FMJY01000007.1
AGNG01000074.1	Fusarium oxysporum f. sp. raphani 54005 cont1.74, whole genome shotgun sequence	2067	2067	100%	0	98.49%	AGNG01000074.1
AGNF01000101.1	Fusarium oxysporum f. sp. conglutinans race 2 54008 cont1.101, whole genome shotgun sequence	2067	2067	100%	0	98.49%	AGNF01000101.1
AGNC01000072.1	Fusarium oxysporum f. sp. vasinfectum 25433 cont1.72, whole genome shotgun sequence	2067	2067	100%	0	98.49%	AGNC01000072.1
AFQF01003431.1	Fusarium oxysporum Fo5176 contig03613, whole genome shotgun sequence	2067	2067	100%	0	98.49%	AFQF01003431.1
RBJF01000172.1	Fusarium oxysporum strain MOD1-FUNGI10 MOD1-FUNGI10_172_length_74869_cov_20.722965, whole genome shotgun sequence	2063	2063	100%	0	98.41%	RBJF01000172.1
RBCF01000115.1	Fusarium oxysporum strain MOD1-FUNGI11 MOD1-FUNGI11_115_length_126873_cov_77.332976, whole genome shotgun sequence	2063	2063	100%	0	98.41%	RBCF01000115.1
MAMH01000155.1	Fusarium oxysporum f. sp. niveum strain Fon019 Fon019blob2c_contig_155, whole genome shotgun sequence	2063	2063	100%	0	98.41%	MAMH01000155.1
MALD01000062.1	Fusarium oxysporum f. sp. niveum strain Fon015 Fon015blob2c_contig_62, whole genome shotgun sequence	2063	2063	100%	0	98.41%	MALD01000062.1
AGBI01000171.1	Fusarium oxysporum f. sp. pisi HDV247 cont1.171, whole genome shotgun	2063	2063	100%	0	98.41%	AGBI01000171.1

	sequence						
MRCX01000035.1	Fusarium oxysporum strain Fo_A13 contig_35, whole genome shotgun sequence	2061	2061	100%	0	98.17%	MRCX01000035.1
MEHF01000029.1	Fusarium oxysporum f. sp. ciceris strain 38-1 Scaffold_0029, whole genome shotgun sequence	2059	2059	100%	0	98.41%	MEHF01000029.1
RBCG01000224.1	Fusarium oxysporum strain MOD1-FUNGI9 MOD1-FUNGI9_224_length_62345_cov_20.573874, whole genome shotgun sequence	2058	2058	100%	0	98.33%	RBCG01000224.1
NJCC01000383.1	Fusarium oxysporum f. sp. melongenae strain J-71 Fomel_001_contig_383, whole genome shotgun sequence	2058	2058	100%	0	98.33%	NJCC01000383.1
MTPZ01000163.1	Fusarium oxysporum strain CS5870 CS5870_contig_166, whole genome shotgun sequence	2058	2058	100%	0	98.33%	MTPZ01000163.1
MAMF01000057.1	Fusarium oxysporum f. sp. melonis strain Fom016 Fom016pilon_contig_57, whole genome shotgun sequence	2058	2058	100%	0	98.33%	MAMF01000057.1
MAME01000138.1	Fusarium oxysporum f. sp. melonis strain Fom013 Fom013pilon_contig_138, whole genome shotgun sequence	2058	2058	100%	0	98.33%	MAME01000138.1
MAMD01000121.1	Fusarium oxysporum f. sp. melonis strain Fom012 Fom012pilon_contig_121, whole genome shotgun sequence	2058	2058	100%	0	98.33%	MAMD01000121.1
MAMC01000118.1	Fusarium oxysporum f. sp. melonis strain Fom011 Fom011pilon_contig_118, whole genome shotgun sequence	2058	2058	100%	0	98.33%	MAMC01000118.1
MAMB01000002.1	Fusarium oxysporum f. sp. melonis strain Fom010 Fom010pilon_contig_2, whole genome shotgun sequence	2058	2058	100%	0	98.33%	MAMB01000002.1
MAMA01000071.1	Fusarium oxysporum f. sp. melonis strain Fom009 Fom009pilon_contig_71, whole genome shotgun sequence	2058	2058	100%	0	98.33%	MAMA01000071.1
MALZ01000033.1	Fusarium oxysporum f. sp. melonis strain Fom006 Fom006pilon_contig_33, whole genome shotgun sequence	2058	2058	100%	0	98.33%	MALZ01000033.1
MALY01000099.1	Fusarium oxysporum f. sp. melonis strain Fom005 Fom005pilon_contig_99, whole genome shotgun sequence	2058	2058	100%	0	98.33%	MALY01000099.1
MALX01000010.1	Fusarium oxysporum f. sp. melonis strain Fom004 Fom004pilon_contig_10, whole genome shotgun sequence	2058	2058	100%	0	98.33%	MALX01000010.1
NJCM01000254.1	Fusarium oxysporum f. sp. gladioli strain G14 Fogla_G14_contig_254, whole genome shotgun sequence	2050	2050	100%	0	98.25%	NJCM01000254.1
MBFV01000645.1	Fusarium oxysporum f. sp. cubense strain C1HIR_9889 ctg7180000050671, whole genome shotgun sequence	2043	2043	100%	0	98.08%	MBFV01000645.1
AMGQ01001041.1	Fusarium oxysporum f. sp. cubense race 4 contig1041, whole genome shotgun sequence	2043	2043	100%	0	98.08%	AMGQ01001041.1
AGND01000065.1	Fusarium oxysporum f. sp. cubense tropical race 4 54006 cont1.65, whole genome shotgun sequence	2043	2043	100%	0	98.08%	AGND01000065.1
NJCH01000321.1	Fusarium oxysporum f. sp. lagenariae strain Lag:3-1 Folag_004_contig_321, whole genome shotgun sequence	2024	2024	100%	0	97.57%	NJCH01000321.1
NJCE01000046.1	Fusarium oxysporum f. sp. luffae strain Fol-114 Foluf_001_contig_46, whole genome shotgun sequence	2024	2024	100%	0	97.57%	NJCE01000046.1
NJCD01000076.1	Fusarium oxysporum f. sp. luffae strain Fol-167 Foluf_002_contig_76, whole genome shotgun sequence	2024	2024	100%	0	97.57%	NJCD01000076.1

AMGP01000730.1	Fusarium oxysporum f. sp. cubense race 1 contig730, whole genome shotgun sequence	2024	2024	100%	0	97.57%	AMGP01000730.1
MABT01000079.1	Fusarium oxysporum f. sp. cucumerinum strain Foc011 Foq011_contig_79, whole genome shotgun sequence	2021	2021	100%	0	97.66%	MABT01000079.1
MABJ01000074.1	Fusarium oxysporum f. sp. cucumerinum strain Foc013 Foq013_contig_74, whole genome shotgun sequence	2021	2021	100%	0	97.66%	MABJ01000074.1
PXUO01000019.1	Fusarium oxysporum strain VEG-01C1 Fusarium_oxysporum_strain_Leaf-19312, whole genome shotgun sequence	2008	2008	100%	0	97.08%	PXUO01000019.1
PXUN01000009.1	Fusarium oxysporum strain VEG-01C2 Fusarium_oxysporum_strain_Leaf-15414, whole genome shotgun sequence	2008	2008	100%	0	97.08%	PXUN01000009.1
MSJJ02000861.1	Fusarium solani strain IMV 00293 jcf7180000048451, whole genome shotgun sequence	2008	2008	100%	0	97.08%	MSJJ02000861.1
BCHB01000007.1	Fusarium commune DNA, scaffold: scaffold_6, strain: JCM 11502, whole genome shotgun sequence	1706	1706	100%	0	91.89%	BCHB01000007.1
NIFK01000007.1	Fusarium udum strain F-02845 scaffold7, whole genome shotgun sequence	1114	1114	100%	0	78.85%	NIFK01000007.1
RBJG01000079.1	Fusarium proliferatum strain MOD1-FUNGI8 MOD1-FUNGI8_79_length_107407_cov_19.723442, whole genome shotgun sequence	977	977	100%	0	75.94%	RBJG01000079.1
QBDQ01000011.1	Fusarium fujikuroi strain COH1152 Fusarium_fujikuroi_strain_COH1152-16973, whole genome shotgun sequence	977	977	100%	0	75.94%	QBDQ01000011.1
MRDB01000006.1	Fusarium proliferatum strain Fp_A8 contig_6, whole genome shotgun sequence	977	977	100%	0	75.94%	MRDB01000006.1
MBPS01000043.1	Fusarium proliferatum strain CF-295141 Contig0000043, whole genome shotgun sequence	977	977	100%	0	75.94%	MBPS01000043.1
RBCE01000027.1	Fusarium proliferatum strain MOD1-FUNGI12 MOD1-FUNGI12_27_length_281258_cov_73.965915, whole genome shotgun sequence	943	943	100%	0	75.11%	RBCE01000027.1
RBCD01000151.1	Fusarium proliferatum strain MOD1-FUNGI13 MOD1-FUNGI13_151_length_84766_cov_31.101241, whole genome shotgun sequence	943	943	100%	0	75.11%	RBCD01000151.1
RBCC01000012.1	Fusarium proliferatum strain MOD1-FUNGI14 MOD1-FUNGI14_12_length_419446_cov_40.606356, whole genome shotgun sequence	943	943	100%	0	75.11%	RBCC01000012.1
RBCB01000024.1	Fusarium proliferatum strain MOD1-FUNGI15 MOD1-FUNGI15_24_length_419438_cov_97.082058, whole genome shotgun sequence	943	943	100%	0	75.11%	RBCB01000024.1
RBBY01000010.1	Fusarium proliferatum strain MOD1-FUNGI19 MOD1-FUNGI19_10_length_419491_cov_16.741182, whole genome shotgun sequence	943	943	100%	0	75.11%	RBBY01000010.1
FJOF01000005.1	Fusarium proliferatum ET1 genome assembly, contig: FPRO_scaffold005, whole genome shotgun sequence	943	943	100%	0	74.98%	FJOF01000005.1
FCQG01000008.1	Fusarium proliferatum strain NRRL62905 genome assembly, contig: FPRN_scaffold008, whole genome shotgun sequence	929	929	100%	0	74.63%	FCQG01000008.1
MTQA01000064.1	Fusarium nygamai strain CS10214 CS10214_contig_65, whole genome shotgun sequence	927	927	100%	0	74.59%	MTQA01000064.1

LBNR01000004.1	Fusarium nygamai strain MRC8546 scaffold0000004, whole genome shotgun sequence	927	927	100%	0	74.59%	LBNR01000004.1
NJCT01000048.1	Fusarium proliferatum strain Fol3 F.proliferatum_Fol3_contig_48, whole genome shotgun sequence	924	924	100%	0	74.55%	NJCT01000048.1
PKMI01000033.1	Fusarium proliferatum strain ITEM 2341 Scaffold_34.1, whole genome shotgun sequence	905	905	100%	0	74.01%	PKMI01000033.1
JRVH01001215.1	Fusarium fujikuroi strain KSU 3368 seq_1215, whole genome shotgun sequence	900	900	100%	0	73.93%	JRVH01001215.1
JRVF01000719.1	Fusarium fujikuroi strain FGSC 8932 seq_719, whole genome shotgun sequence	900	900	100%	0	73.93%	JRVF01000719.1
FMSM01000008.1	Fusarium fujikuroi strain FSU48 genome assembly, contig: FFFS_scaffold008, whole genome shotgun sequence	900	900	100%	0	73.93%	FMSM01000008.1
FMSL01000012.1	Fusarium fujikuroi strain B14 genome assembly, contig: FFB14_scaffold012, whole genome shotgun sequence	900	900	100%	0	73.93%	FMSL01000012.1
FMJX01000011.1	Fusarium fujikuroi strain NCIM1100 genome assembly, contig: FFNC_scaffold011, whole genome shotgun sequence	900	900	100%	0	73.93%	FMJX01000011.1
FMJW01000007.1	Fusarium fujikuroi strain MRC2276 genome assembly, contig: FFMR_scaffold007, whole genome shotgun sequence	900	900	100%	0	73.93%	FMJW01000007.1
FMJV01000008.1	Fusarium fujikuroi strain m567 genome assembly, contig: FFM5_scaffold008, whole genome shotgun sequence	900	900	100%	0	73.93%	FMJV01000008.1
FMJU01000012.1	Fusarium fujikuroi strain C1995 genome assembly, contig: FFC1_scaffold012, whole genome shotgun sequence	900	900	100%	0	73.93%	FMJU01000012.1
FMJT01000008.1	Fusarium fujikuroi strain E282 genome assembly, contig: FFE2_scaffold008, whole genome shotgun sequence	900	900	100%	0	73.93%	FMJT01000008.1
FMJS01000065.1	Fusarium fujikuroi genome assembly, contig: FFB20_contig065, whole genome shotgun sequence	900	900	100%	0	73.93%	FMJS01000065.1
FCQH01000011.1	Fusarium mangiferae strain MRC7560 genome assembly, contig: FMAN_scaffold011, whole genome shotgun sequence	900	900	100%	0	74.28%	FCQH01000011.1
ANFV01000015.1	Fusarium fujikuroi B14 contig5_2, whole genome shotgun sequence	900	900	100%	0	73.93%	ANFV01000015.1
MBPO01001686.1	Fusarium fujikuroi isolate F250 F250_Contig2613, whole genome shotgun sequence	895	895	100%	0	73.85%	MBPO01001686.1
PDNT01000009.1	Fusarium fracticaudum strain CBS 137234 scaffold9, whole genome shotgun sequence	891	891	100%	0	73.63%	PDNT01000009.1
JRVG01000091.1	Fusarium fujikuroi strain KSU X-10626 seq_91, whole genome shotgun sequence	891	891	100%	0	73.65%	JRVG01000091.1
NFZR01000008.1	Fusarium pininemorale strain CMW 25243 contig0000008_size1474616, whole genome shotgun sequence	874	874	100%	0	73.06%	NFZR01000008.1
LUFC01001544.1	Fusarium agapanthi strain NRRL 31653 contig1544, whole genome shotgun sequence	850	850	100%	0	72.62%	LUFC01001544.1
NJCS01000014.1	Fusarium sp. Na10 Fusarium.sp._Na10_contig_14, whole genome shotgun sequence	838	838	100%	0	72.16%	NJCS01000014.1
PVPY01000445.1	Fusarium algeriense strain NRRL 66648 Fusarium_sp_1248(KOD)_contig_0000445, whole genome shotgun sequence	592	592	100%	5.00E -166	70.76%	PVPY01000445.1

PVQA01000721.1	Fusarium burgessii strain NRRL 66654 Fspec_KOD1446b_contig_0000721, whole genome shotgun sequence	588	588	100%	2.00E -164	70.50%	PVQA01000721.1
RXHO01000540.1	Fusarium xylarioides strain K1 scaffold_540, whole genome shotgun sequence	1120	1120	99%	0	79.20%	RXHO01000540.1
MPIL01001527.1	Fusarium oxysporum f. sp. melongenae strain 14004 scaffold_001527, whole genome shotgun sequence	1004	1004	99%	0	78.29%	MPIL01001527.1
QJUS01000069.1	Fusarium verticillioides strain BRIP53263 BRIP53263_contig_71, whole genome shotgun sequence	664	664	99%	0	71.12%	QJUS01000069.1
QFXM01000537.1	Fusarium verticillioides strain BRIP14953 BRIP14953_contig_542, whole genome shotgun sequence	633	633	99%	6.00E -178	70.18%	QFXM01000537.1
AAIM02000084.1	Fusarium verticillioides 7600 chromosome 7 cont3.84, whole genome shotgun sequence	563	563	99%	8.00E -157	68.59%	AAIM02000084.1
NJCK01000203.1	Fusarium oxysporum f. sp. gladioli strain G76 Fogla_G76_contig_203, whole genome shotgun sequence	2021	2021	98%	0	97.97%	NJCK01000203.1
PVPZ01001865.1	Fusarium algeriense strain NRRL 66647 Fusarium_sp_1247(KOD)_contig_0001865, whole genome shotgun sequence	605	605	97%	8.00E -170	71.23%	PVPZ01001865.1
LJGR01000011.1	Fusarium temperatum strain CMWF389 Scaffold11, whole genome shotgun sequence	577	910	97%	4.00E -161	72.57%	LJGR01000011.1
AFML01000096.1	Fusarium sp. FOSC 3-a cont1.96, whole genome shotgun sequence	1574	1798	90%	0	96.82%	AFML01000096.1
PVQB01000194.1	Fusarium beomiforme strain NRRL 25174 F_beomiforme_25174_contig_0000194, whole genome shotgun sequence	359	590	83%	2.00E -95	74.04%	PVQB01000194.1
QKXB01000137.1	Fusarium verticillioides strain BRIP53590 BRIP53590_contig_156, whole genome shotgun sequence	553	596	82%	4.00E -154	71.17%	QKXB01000137.1
JRVE01000037.1	Fusarium circinatum strain GL1327 Scaffold37, whole genome shotgun sequence	371	687	68%	3.00E -99	76.15%	JRVE01000037.1
AYJV02000016.1	Fusarium circinatum strain FSP 34 contig16, whole genome shotgun sequence	371	728	68%	3.00E -99	76.15%	AYJV02000016.1
NJCR01001805.1	Fusarium hostae strain Hy9 F.hostae_Hy9_contig_1805, whole genome shotgun sequence	627	848	67%	2.00E -176	83.69%	NJCR01001805.1
NJCQ01001525.1	Fusarium hostae strain Hy14 F.hostae_Hy14_contig_1525, whole genome shotgun sequence	627	848	67%	2.00E -176	83.69%	NJCQ01001525.1
LQBB01000040.1	Fusarium circinatum isolate KS17 contig0000040, whole genome shotgun sequence	376	642	64%	7.00E -101	76.35%	LQBB01000040.1
LTDI01000562.1	Fusarium agapanthi strain NRRL 54464 contig0562, whole genome shotgun sequence	503	503	56%	7.00E -139	74.60%	LTDI01000562.1
LTDI01000272.1	Fusarium agapanthi strain NRRL 54464 contig0272, whole genome shotgun sequence	410	410	42%	3.00E -111	72.94%	LTDI01000272.1

Table S5. Primers used in this work. Sizes of the expected PCR products (base pairs) are indicated on the right column.

Species and purpose	Primer #	Forward	Reverse	Size
**A. Determination of *carP* orientation**				
F. oxysporum				
cDNA *carP* (F)	Set 1	AGTAGCGTCGATGTGCCAGTTG		
		TGCGGACCGTCCAAGAGT		
		AAAACAGCAAAAGCAGGGAACT		
		GGGCAGTGCAGTTGGTGTT		
cDNA *carP* (R)	Set 2		CGCCGATCTTGCTCTTGTTT	
			CGAGGGACGGAGCCTAAAC	
			TCATGACGTCGAGGCAAAAC	
			TTTAGACGCCTCCAGTCTAAGC	
PCR *carP*	Set 3	AGTAGCGTCGATGTGCCAGTTG	TTTAGACGCCTCCAGTCTAAGC	1099
cDNA β-tub (F)	Primer 4	AGGTCATTAGCATCAACGTTG		
cDNA β-tub (R)	Primer 5		AGAAAGTGAAGACCATGCCTC	
PCR β-tub	Set 6	AGGTCATTAGCATCAACGTTG	AGAAAGTGAAGACCATGCCTC	1169
F. fujikuroi				
cDNA *carP* (F)	Set 7	GCCGAGAGTCCCTTTTACGA		
		CCATTGAGCTGGGATGTGTTTT		
		CTCGGCGTCATGAGTCCAT		
cDNA *carP* (R)	Set 8		GCGCAGTCGAAAAAATGCA	
			TGCGCTGTGCTGTAAACCA	
			CACAACAGCAGCATCAACTTCTG	
PCR *carP*	Set 9	GCCGAGAGTCCCTTTTACGA	CACAACAGCAGCATCAACTTCTG	910
cDNA β-tub (F)	Primer 10	ACCATGGACGCCGTCCGTGC		
cDNA β-tub (R)	Primer 11	GTTCTTGGGGTCGAACATCTG		
PCR β-tub	Set 12	ACCATGGACGCCGTCCGTGC	GTTCTTGGGGTCGAACATCTG	693
**B. Independence of *carP* and *carS* transcripts**				
F. oxysporum				
PCR *carP*	Set 13	CGTCGATGCGCCAGTTGATT	AGCAAGCGCCTAGTGGCC	1172

PCR *carP*/*carS*	Set 14	CTGTTGTGTGACTCGAGAG TTC	GCAGGGGAGGCTGATGGG CT	1922
PCR *carS*	Set 15	TTCTCTAGAGTACTATACG CACGCAA	CAGGTCGACGGGCAAGCA TTGTCAATCAGA	2036
F. fujikuroi				
PCR *carP*	Set 16	CGTCGATGCGCCAGTTGAT T	AGCAAGCGCCTAGTGGCC	1245
PCR *carP*/*carS*	Set 17	CTGTTGTGTGACTCGAGAG TTC	GCAGGGGAGGCTGATGGG CT	2002
PCR *carS*	Set 18	TTCTCTAGAGTACTATACG CACGCAA	CAGGTCGACGGGCAAGCA TTGTCAATCAGA	2029
**C. Plasmid constructions**				
hyg^R cassette	Set 19	GTCGGAGACAGAAGATGA TATTGAAGGAGC	GTTGGAGATTTCAGTAACG TTAAGTGGAT	1447
pDul14 *F. oxysporum*				
5' *carP* segment	Set 20	GTAACGCCAGGGTTTTCCC AGTCACGACGTGAATAAC GGAATTGCCTAGA	ATCCACTTAACGTTACTGA AATCTCCAACTAGTTGAGA GGGAAACCTGCC	1457
3' *carP* segment	Set 21	CTCCTTCAATATCATCTTCT GTCTCCGACAGAGTAACG ACCACCTCCCAC	GCGGATAACAATTTCACA CAGGAAACAGCGGGGCAG TGAGCAGATCTGAC	1539
Replacement cassette	Set 22	GTAACGCCAGGGTTTTCCC AGTCACGACGTGAATAAC GGAATTGCCTAGA	GCGGATAACAATTTCACA CAGGAAACAGCGGGGCAG TGAGCAGATCTGAC	4437
pDul8 *F. oxysporum*				
5' *carP** segment	Set 23	GTAACGCCAGGGTTTTCCC AGTCACGACGGATGACAA GTGCAAGGCTTGA	ATCCACTTAACGTTACTGA AATCTCCAACCCCTCACTC ATCTCGATAATCG	1193
3' *carP** segment	Set 24	CTCCTTCAATATCATCTTCT GTCTCCGACGTAGAGTAAC GACCACCTCC	GCGGATAACAATTTCACA CAGGAAACAGCAAAAGGG GTTGTATAAGGTAGG	790
Replacement cassette	Set 25	GTAACGCCAGGGTTTTCCC AGTCACGACGGATGACAA GTGCAAGGCTTGA	GCGGATAACAATTTCACA CAGGAAACAGCAAAAGGG GTTGTATAAGGTAGG	3424
F. fujikuroi				
5' *carP* segment	Set 26	GTAACGCCAGGGTTTTCCC AGTCACGACGGGAGATGG TCTAGAACAATTG	ATCCACTTAACGTTACTGA AATCTCCAACATCGACGCT ACTTAATAGTCGAG	1233
3' *carP* segment	Set 27	CTCCTTCAATATCATCTTCT GTCTCCGACGGCGCTTGCT AGGAACTGCC	GCGGATAACAATTTCACA CAGGAAACAGCGCCTTGA CCGTTATATGATGTG	1315
Replacement cassette	Set 28	GGAGATGGTCTAGAACAA TTG	GCCTTGACCGTTATATGAT GTG	3879
**D. Molecular characterization of transformants**				
F. oxysporum Δ*Fo-carP*				
PCR test	Set 29	GTAACGCCAGGGTTTTCCC AGTCACGACGTGAATAAC GGAATTGCCTAGA	GCGGATAACAATTTCACA CAGGAAACAGCGGGGCAG TGAGCAGATCTGAC	4436
Southern probe	Set 30	CACGCTATCAACGGCTACG CC	TTGAGTCGTTACTCTTCGT TGTGTC	256

F. oxysporum Δ*Fo-carP**				
PCR test	Set 31	GAGTCGAGAGAGAAGCGAGA	GTGTAGAGATTGGTGGGGTT	2760
Southern probe	Set 32	GCCTCTAATAATCATCGGGA	GCCAATCCATTTCCTCAACCT	344
F. fujikuroi Δ*Ff-carP*				
PCR test 5’*carP* - Hyg[R]	Set 33	GATGAGTGTGTGAGTGTGTGATGTTG	GTCGGAGACAGAAGATGATATTGAAGGAGC	3705
PCR test 3’*carP* - Hyg[R]	Set 34	CCTTCAAATATGTACCGCGTAGACTATG	GTTGGAGATTTCAGTAACGTTAAGTGGAT	2223
PCR test 5’*carP*	Set 35	GATGAGTGTGTGAGTGTGTGATGTTG	AGCAAGCGCCTAGTGGCC	3496
PCR test 3’*carP*	Set 36	CGTCGATGCGCCAGTTGATT	AGCAAGCGCCTAGTGGCC	1245
Southern probe	Set 37	CCATTTCTGTTCCCTTCCCTG	CCGTCATACACCAGAGAGAC	412
**E. RT-qPCR**				
F. oxysporum genes				
carP	Set 38	TGCGGACCGTCCAAGAGT	CGCCGATCTTGCTCTTGTTT	60
*carP**	Set 39	GGGCAGTGCAGTTGGTGTT	TCATGACGTCGAGGCAAAAC	86
carS FOXG_09307	Set 40	TCCTCGAGGCTAACATCGTC	CCTTGACGGATACATCGTCG	181
carRA FOXG_12144	Set 41	CATCCAGCGCGAAGTTGTC	CGCGCGATATTGACGTATTG	69
carB FOXG_12143	Set 42	GCGCAAGAATAGGCGTTGAT	CCATGTGAAGGGAGTGTTGATG	68
carO FOXG_12142	Set 43	GCTCGTCTGCACGCTAAACA	CGCACATGACAAAGGTTCGA	56
carX FOXG_12145	Set 44	GTTCCCGCGGCATCAA	ACAGCATGGCGCTGTATGAA	55
carD FOXG_05463	Set 45	GATGAGCCTGTCCCCAATGT	GCGAGTGCGGTGCTTCA	57
carT FOXG_10608	Set 46	TGCGCTTTTGGCGAATGT	TCGACACCTCCGTCTTCTCAT	56
cryD FOXG_03570	Set 47	CACCTCAGGAACCTCCGTTTC	ATAGGCTGGAGGGTCTTGCA	101
vvdA FOXG_03254	Set 48	GTACGGACAAGGTCGCTTCAC	GAAGGGCTGGCCATATTTCTT	101
wc1 FOXG_03727	Set 49	AGTAACGGCACCGAAAACCA	TTGTCCTCTGAGGCCAAAGC	101
con-10 ortholog FOXG_01269	Set 50	CTCTTTTGAGCCTGGTAGTGAGAAG	CATCGATCCAAAGTCATGTCTCAT	101
β-tub FOXG_06228	Set 51	CCGGTGCTGGAAACAACTG	CGAGGACCTGGTCGACAAGT	69
F. fujikuroi genes				
carP (1)	Set 52	GCCGAGAGTCCCTTTTACGA	GCGCAGTCGAAAAAATGCA	67
carP (2)	Set 53	CCATTGAGCTGGGATGTGTTTT	TGCGCTGTGCTGTAAACCA	69
carS FFUJ_08714	Set 54	GATACCCGGCGGAAAGGTTA	CTGACAGTCCATTTCAGCGC	181

carRA FFUJ_11802	Set 55	CAGAAGCTGTTCCCGAAGACA	TGCGATGCCCATTTCTTGA	65
carB FFUJ_11803	Set 56	TCGGTGTCGAGTACCGTCTCT	TGCCTTGCCGGTTGCTT	68
cryD FFUJ_05732	Set 57	CGGGACTACATGCGATTGTG	CTTGAAAAGACGTGAGCCAAACT	51
vvdA FFUJ_06055	Set 58	GCACCACCAGGGCATGA	GCGGTGTGAAGCGACCTT	63
wcoA FFUJ_13691	Set 59	TGAGATTGTCGGCCAGAATTG	GAGCCCGCTTCGACTTTG	63
β-tub FFUJ_04397	Set 60	CCGGTGCTGGAAACAACTG	CGAGGACCTGGTCGACAAGT	69
gpdh FFUJ_13490	Set 61	GTGACCTCAAGGGCGTTCTG	CGAAGATGGAGTTTGTGTT	94
